# Tetrasubstituted Peropyrenes Formed by Reductive Aromatization: Synthesis, Functionalization and Characterization

**DOI:** 10.1002/chem.202101101

**Published:** 2021-06-15

**Authors:** Simon Werner, Tobias Vollgraff, Jörg Sundermeyer

**Affiliations:** ^1^ Fachbereich Chemie and Material Science Center Philipps-Universität Marburg Hans-Meerwein-Straße 4 35032 Marburg Germany

**Keywords:** cyclic voltammetry, crystallography, fluorescence, peropyrenes, reductive aromatization

## Abstract

The chromophore class of 1,3,8,10‐tetrasubstituted peropyrenes was effectively synthesized from peropyrenequinone via a Zn‐mediated reductive aromatization approach. In one step, a symmetric functionalization of the peropyrene backbone introducing silylethers (**2**,**3**), pivaloyl (**4**), triflyl (**5**) and also phosphinite (**6**) groups was established. Furthermore, the potential of using **4** and **5** in transition metal catalysed cross couplings was explored leading to 1,3,8,10‐tetraaryl (**8**‐**11**) and tetraalkynyl (**7**) peropyrenes. The influence of various substituents on the optoelectronic properties of these π‐system extended peropyrenes was investigated in solid state by means of X‐ray crystallography, in solution by means of UV‐Vis and fluorescence spectroscopy and by their redox properties studied via cyclic voltammetry. By comparison with DFT and TD‐DFT calculations, it could be elucidated that introduction of a broad variety of substituents in such versatile one or two step procedures leads to peropyrenes with easily tunable HOMO and LUMO energies ranging in a gap window of 0.8 eV. The frontier molecular orbital energies identify the target molecules as promising candidates for hole transporting semiconductors.

## Graphical abstract

**1,3,8,10‐Tetrasubstituted peropyrenes** were synthesized via a reductive aromatization and functionalization strategy. Finetuning of their optoelectronic properties was monitored by UV‐Vis, PL, XRD, CV, and DFT methods.

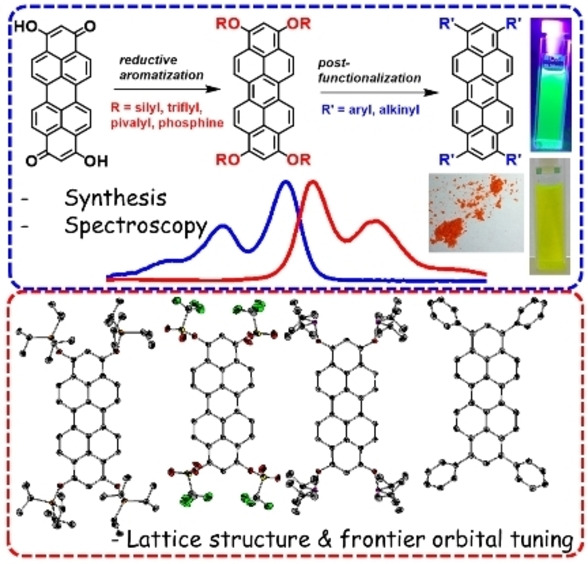

## Introduction

**Scheme 1:**
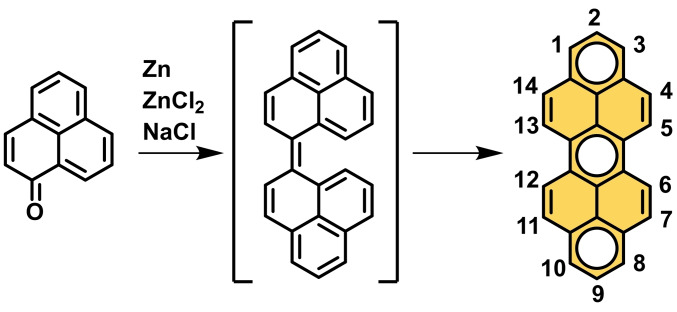
Clar‐synthesis of peropyrene and the in situ formed biphenalenylidene intermediate. The C−H positions of peropyrene are numbered and the π‐sextets according to Clar are highlighted.[^7^, ^8^]

Polyaromatic Hydrocarbons (PAHs) with atomically precise defined structures have received broad research interest in the past decade because of their manifold applications, for example their use in organic light‐emitting diodes (OLED) or organic field effect transistors (OFET) and organic solar cells (OSC).[^1^, ^2^, ^3^, ^4^] One of the first versatile synthetic approaches to PAHs were described at the beginning of the past century by Scholl et al.[^5^, ^6^] In 1943, Clar reported the synthesis of dibenzoperylene, named peropyrene because of its structural consistence of both PAH systems perylene and pyrene.^[7]^ Peropyrene served as model for studying the aromaticity of different benzene rings in PAH and let him define the aromatic π‐sextet rule with respect to the stability of aromatic systems (see Scheme 1).[^8^, ^9^, ^10^] Since then, research on peropyrene has been neglected for some decades. This changed in the context of systematic investigations of the relationship between structure and (optical) properties of PAHs.^[11]^ For example in polyacenes such as pentacene,[^12^, ^13^] large energetical separations between the triplet (T_1_) and the singlet (S_1_) state made it possible to consider these compounds as singlet fission materials potentially utilizable for solar cell applications.[^14^, ^15^, ^16^] This photophysical process was also observed in perylene[^17^, ^18^] and perylene diimides.^[19]^ Earlier work reported on photoconductivity^[20]^ and fluorescence quenching properties^[21]^ of peropyrene in solution suggesting that peropyrene could also be a singlet fission material in solution. In 2013, Bardeen and co‐workers reported on the photophysical properties of peropyrene in solution and solid state, showing that the S_1_ energy is significantly lowered in crystalline state.^[22]^

However, the use of peropyrenes as a crystalline singlet fission material requires the development of synthesis strategies for substituted peropyrenes with tunable properties both in solution and film as well as in the crystalline state. The original synthesis by Clar yielded peropyrene via reductive coupling of 1‐phenalenone using a salt melt consisting of Zn, ZnCl_2_ and NaCl under harsh conditions at 300 °C (Scheme 1).^[7]^

Pogodin, Agranat and co‐workers modified the reductive dimerization strategy using milder conditions such as low valent titanium reagents related to McMurry reaction conditions with TiCl_4_/LiAlH_4_ in tetrahydrofuran.^[23]^ Alternatively, P_2_S_5_ in benzene^[24]^ was used in order to dimerize two phenalenone radicals to biphenalenylidene, which in situ forms peropyrene via electrocyclization. Experimental evidence for this mechanism was reported by Uchida et al. who isolated dihydroperopyrene, the electrocyclization product of biphenalenylidene, which forms peropyrene after oxidation.^[25]^ Beer et al. used the dimerization of phenalenyl radical cations in the synthesis of functionalized peropyrenes.^[26]^ Peropyrene was also synthesized via intramolecular dehydrogenative C−C‐bond coupling of metacyclophane and subsequent oxidation using DDQ.^[27]^

**Scheme 2:**
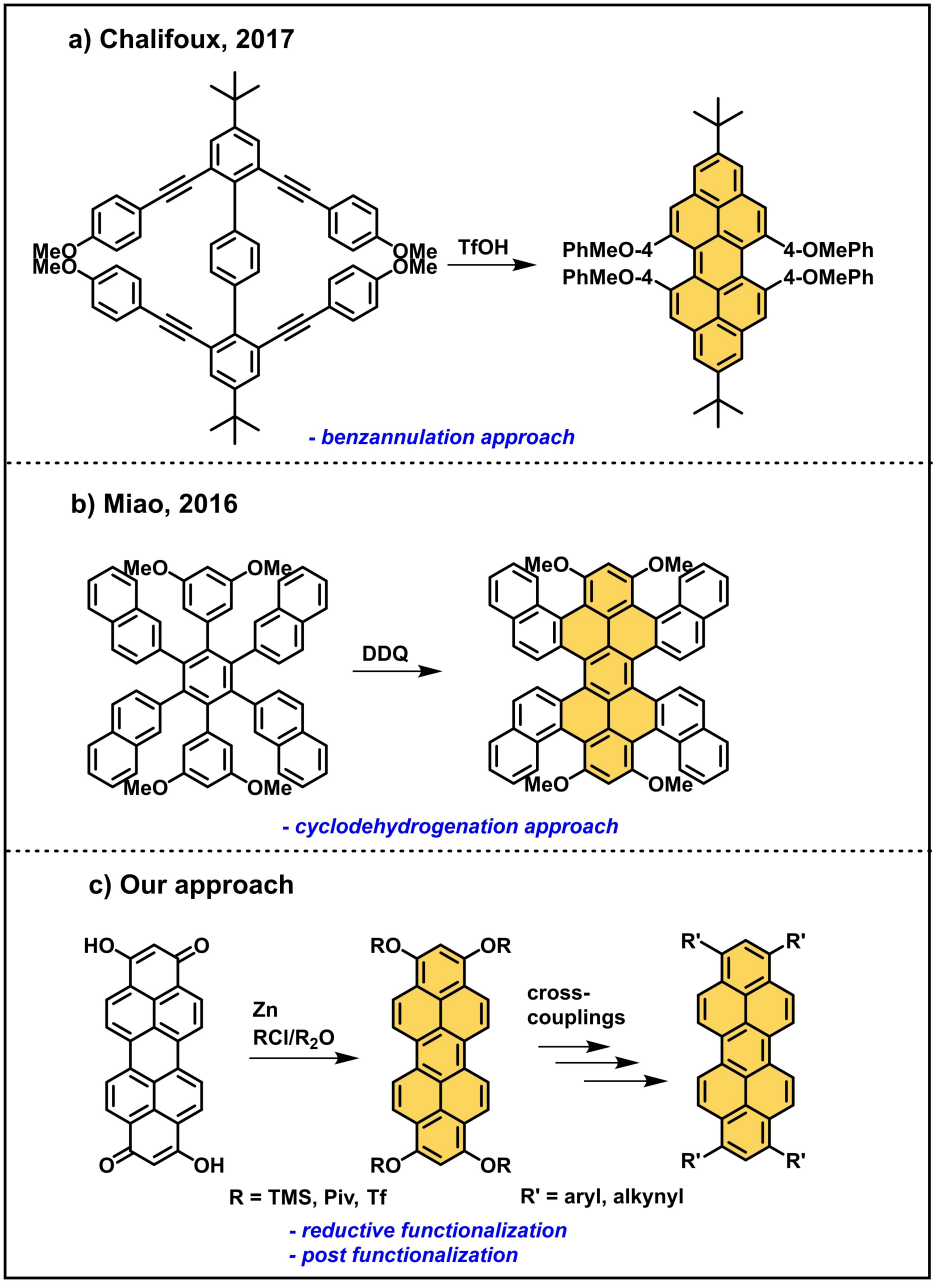
a) TfOH‐mediated benzannulation approach for the synthesis of bay‐substituted peropyrens by Chalifoux,^[30]^ b) Miao's synthesis of highly twisted peropyrenes via Scholl reaction,^[32]^ and c) our approach for the synthesis of 1,3,8,10‐tetrasubstituted peropyrenes via reductive aromatization and post‐functionalization.

So far, approaches for the synthesis of functionalized peropyrenes in 2‐ and 9‐position require multistep synthesis protocols.^[28]^ A more convenient protocol was developed by Chalifoux and co‐workers who built up the peropyrene scaffold by acid mediated intramolecular benzannulation of internal alkynes (Scheme 2a).[^29^, ^30^, ^31^] This approach enables the functionalization of the bay positions of peropyrene and leads therefore to twisted peropyrens with up to 18° out of plane twist angle and axial chirality.^[30]^ The most twisted representative is a tetranaphthyl annulated peropyrene built up via oxidative Scholl dehydrogenation of a tetranaphthyl‐diphenylbenzene precursor reported by the group of Miao (Scheme 2b)^[32]^ and later extended with pyrene functionalities by Feng.^[33]^ Recently, these authors also accessed a dicyclopenta‐fused peropyrene via alkyne annulation approach.^[34]^

Since many peropyrene syntheses require low‐yield aromatization steps or multistep synthesis approaches, we aimed to establish a higher yield synthetic approach with only a short number of steps. Thereby we were able to obtain peropyrenes with a yet undescribed 1,3,8,10‐substitution pattern. Recently, the group of Mateo‐Alonso described a reductive mono‐ and dialkylation of a non‐hydroxylated peropyrenequinone.[^35^, ^36^] But they could not separate the two formed 1,8‐ and 1,9‐isomers.^[36]^ We used bis‐hydroxylated peropyrenequinones^[37]^ as precursors, allowing an new approach to symmetric fourfold peropyrene substitution. Recently, we demonstrated the potential of a reductive O‐silylation strategy in the synthesis of peropyrene's higher homologues, terropyrene and quarterropyrene.^[38]^ As a key step, we modified and applied the reductive aromatization step recently reported for the reductive functionalization of perylene diimide (PTCDI)[^39^, ^40^] by using Zn instead of Na as bench stable and less powerful reducing agent. In the following we add proof, that this protocol is perfectly suitable for the introduction of different functional groups via post‐functionalization of reactive triflyl and pivaloyl peropyrene key intermediates (Scheme 2c).

## Results and Discussion

### Synthesis

**Scheme 3:**
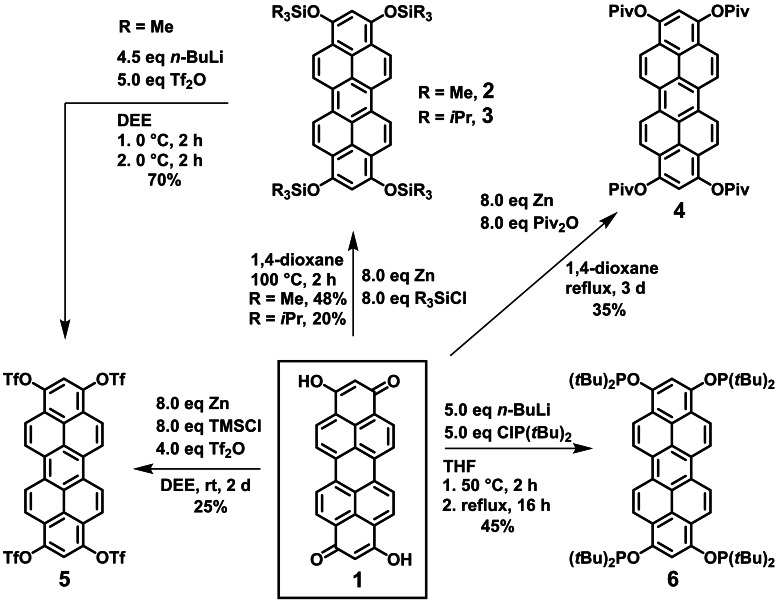
Reductive aromatization strategies to convert peropyrenequinone **1** to the corresponding peropyrene silylethers **2** and **3**, pivaloate **4**, triflate **5** and phosphinite **6** under mild conditions using Zn or *n*‐butyl lithium as reducing agent.

Starting point for our reductive aromatization strategy was peropyrenequinone (**1**), synthesized according to a protocol of Bock.^[37]^ **1** is a purple pigment, which is almost insoluble in common organic solvents. However, when suspended together with an excess (8 eq) of zinc dust and trimethylsilyl chloride in 1,4‐dioxane at 60–80 °C, a green, fluorescent solution appears which yields the corresponding reduced 1,3,8,10‐tetrasiloxy peropyrene **2** in a yield of 48 % (Scheme 3). Reductive trimethylsilylations are already known for anthracene‐9,10‐dione. They are privileged due to the activation of zinc by trimethylsilyl chloride.^[41]^

The analogous reaction conditions using Mg turnings instead of Zn dust yielded only traces of product **2**, whereas the reaction failed completely when Na or C_8_K were used as reducing agent instead. This can be explained with the higher reduction potential of Na and C_8_K compared to Zn, which might lead to undesirable overreduction to product radical anions or activation of the relatively acidic protons of peropyrenequinone in 2‐ and 9‐position. The moderately air sensitive orange solid **2** could be easily purified from zinc dichloride and excess zinc by dissolving the crude product in dichloromethane, filtration and removing the solvent, since the polymeric zinc chloride dioxane adduct is insoluble in chlorinated solvents.^[38]^ An air stable product can be obtained, when **1** is reacted with triisopropylsilyl chloride (TIPSCl) instead of TMSCl. Notable, a moderate yield of 20 % of peropyrene **3** could only be achieved by using imidazole as equimolar activation reagent for triisopropylsilyl chloride. Chemically more robust **3** could be purified by column chromatography on silica. Its lower reactivity compared to **2** is due to the higher steric demand of TIPSCl versus TMSCl. In order to synthesize peropyrenes with carboxyl ester groups in 1,3,8,10‐position which provide a possibility for further functionalization, i. e. in cross coupling reactions, a direct reduction of **1** to corresponding tetrapivaloyl‐substituted peropyrene **4** was accomplished in moderate yield (35 %) by refluxing **1** with 8 eq of zinc dust and pivalic anhydride for 3 days. Recently, this reductive pivalation strategy was also used Miyake et al. for their functionalization of naphthalene diimide (NTCDI) to tetrapivaloxy‐2,7‐diazapyrene^[42]^ and of perylene diimide (PTCDI) to tetrapivaloxy‐2,7‐diazaperopyrene.^[43]^ The corresponding pivaloates were subjected to Ni‐catalysed cross couplings. In contrast to trimethylsilyl ether **2**, pivalate **4** is air stable and moisture insensitive, thus a purification by column chromatography on silica was possible.

**Scheme 4:**
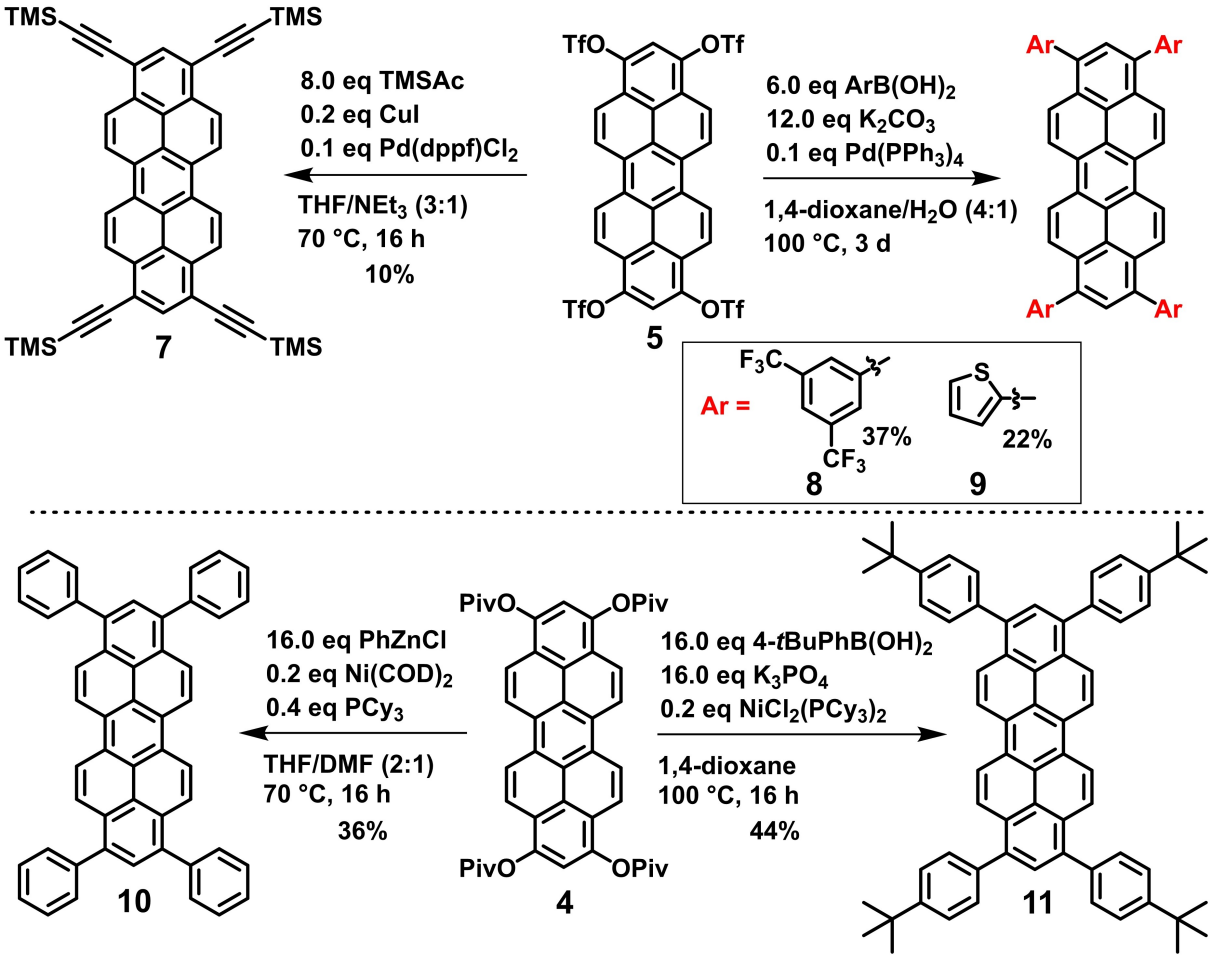
Post‐functionalization of triflate **5** and pivalate **4** in Ni and Pd catalysed cross‐coupling reactions for the formation of alkenyl (**7**)‐ and aryl (**8**‐**11**)‐functionalized peropyrenes.

We discovered, that silylether **2** can easily be deprotected and activated via stoichiometric reaction with 4 eq. of *n*‐butyl lithium at 0 °C in diethyl ether: Desilylation is observed, formation of silane *n*‐BuSiMe_3_ and a purple suspension of 1,3,8,10‐tetralithoxy peropyrene. Slow addition of trifluoromethanesulfonic acid anhydride (Tf_2_O) to this in situ prepared suspension of an activated form of **2** allowed to isolate tetratriflate **5** in good yield of 70 %, whereas lithium triflate remains in solution. Due to the high reactivity of the triflate groups, **5** is air sensitive. Direct treatment of **1** with zinc, TMSCl and Tf_2_O also yields **5** in a one‐pot approach, however in lower yields (25 %) and with a larger amount of undesired side products difficult to separate. **1** could be directly reduced and converted to 1,3,8,10‐tetralithoxy peropyrene by applying 6 eq. of *n*‐butyl lithium as base and reducing agent in tetrahydrofurane (THF). By treating this tetra‐lithoxy intermediate with di‐*tert*‐butylchlorophosphine in THF, air sensitive peropyrene 1,3,8,10‐tetraphosphinite **6** was obtained in good yield (45 %). After an *ortho‐*directed CH‐activation, **6** might serve as a PCP pincer ligand[^44^, ^45^, ^46^, ^47^, ^48^, ^49^] for binuclear transition metal complexes. This fourfold O‐phosphination can also be accomplished starting from trimethylsilyl ether **2**, its desilylation with *n*‐BuLi and quenching in situ formed ArO−Li intermediates by *t*‐Bu_2_PCl. Contrastingly, the in situ Zn reduction method failed, since chlorophosphines are prone to form P−P bonds^[50]^ under reductive conditions needed to reduce peropyrenequinone **1**. After discovering the reductive functionalization of **1** with activatable groups, we explored the scope of possible post functionalization reactions. Therefore, pivaloate **4** and triflate **5** were subjected to transition metal catalysed cross coupling reactions (Scheme 4).

Aryl triflates are known to display similar reactivity in Pd catalysed cross coupling reactions as aryl bromides.^[51]^ Therefore, triflate **5** was first reacted with trimethylsilyl acetylene under Sonogashira cross coupling conditions at 2.5 mol % ratio per triflate functional group (10 mol % per **5**).^[52]^ Under optimized conditions, using CuI and [Pd(dppf)Cl_2_] as catalyst in a mixture of triethylamine and tetrahydrofuran, 10 % of the corresponding peropyrene‐tetraalkyne **7** was isolated as a dark red solid after preparative TLC. We suppose that the rather low yield results from a partial product decomposition during the necessary chromatographic purification. So far, other alkyne substituents (e. g. phenylacetylene) only led to complex inseparable product mixtures under these reaction conditions.

Next, we reacted **5** under typical Suzuki‐Miyaura cross coupling conditions using [Pd(PPh_3_)_4_] as catalyst and potassium carbonate as mild base in dioxane/water and 2.5 mol % catalyst ratio per triflate functional group (10 mol % per **5**). Two representative reactions using 2‐thienylboronic acid and 3,5‐bis(trifluoromethyl)phenyl boronic acid were chosen in order to synthesize tetraaryl‐peropyrenes with four electron‐donating 1‐thienyl groups or four moderately electron‐withdrawing 1,3‐trifluromethylphenyl groups. Both tetraaryl‐peropyrenes **8** and **9** were isolated in 37 % (**8**) and 22 % (**9**) yield as air stable orange‐reddish solids after column chromatography. Interestingly, no impurities from side‐products bearing less than four aryl groups were detected under this protocol. Nevertheless, the moderate yields show that triflate **5** tends to decompose to a certain extent, probably due to its limited stability towards water/K_2_CO_3_.

Aryl pivaloates had been developed to be reactive substrates in Ni catalysed cross‐coupling reactions. They might serve as economically and ecologically friendlier alternative to Pd‐catalysed Ar−Ar’ couplings of aryl halides and triflates.[^53^, ^54^, ^55^, ^56^, ^57^, ^58^] Therefore, we explored the reactivity of tetrapivaloate **4** in two representative Ni‐catalysed cross‐couplings. The first uses [Ni(COD)_2_] as Ni(0) precatalyst and tricyclohexylphosphine in 5 mol % ratio per pivaloate functional group (20 mol % per **4**) and aryl zinc reagents in a Negishi‐type cross‐coupling protocol.^[54]^ Thus, an excess of PhZnCl prepared in situ from PhLi and ZnCl_2_ in THF converted **4** into tetraphenyl peropyrene **10** in 34 % yield after column chromatography. Improved Ni‐catalysed cross coupling protocols use the cheaper and bench stable precatalyst [NiCl_2_(PCy_3_)_2_] and an excess of an aryl boronic acid as coupling reagent and reducing agent for the Ni(II) precatalyst.^[56]^ Under such water‐free Suzuki‐Miyaura cross‐coupling conditions in dioxane with K_3_PO_4_ as base,^[56]^ it was possible to synthesize 4‐*tert*‐butylphenyl substituted peropyrene **11**. The yield 44 % seems to be moderate, nevertheless it is 81 % (av.) per coupling step, slightly higher than in the Pd‐catalysed cross‐couplings of triflate **5**. Comparable yields were obtained in Ar−Ar’ couplings with tetrapivaloxy‐2,7‐diazapyrenes.^[42]^ However, the scope of achievable peropyrene substituents compatible with the Ni catalysed cross coupling protocol is limited. Other examples of aryl derivatives such as **8** or **9** could not be synthesized under nickel mediated conditions. In conclusion, pivaloate **4** turned out to be a more stable but less reactive alternative to triflate **5** for the synthesis of tetraaryl peropyrenes. All synthesized tetraaryl‐peropyrenes **8**–**11** show an excellent stability towards water and air which makes them to promising candidates for further investigations applying them in organic semiconductor or OLED devices.

### Crystallography

**Figure 1:**
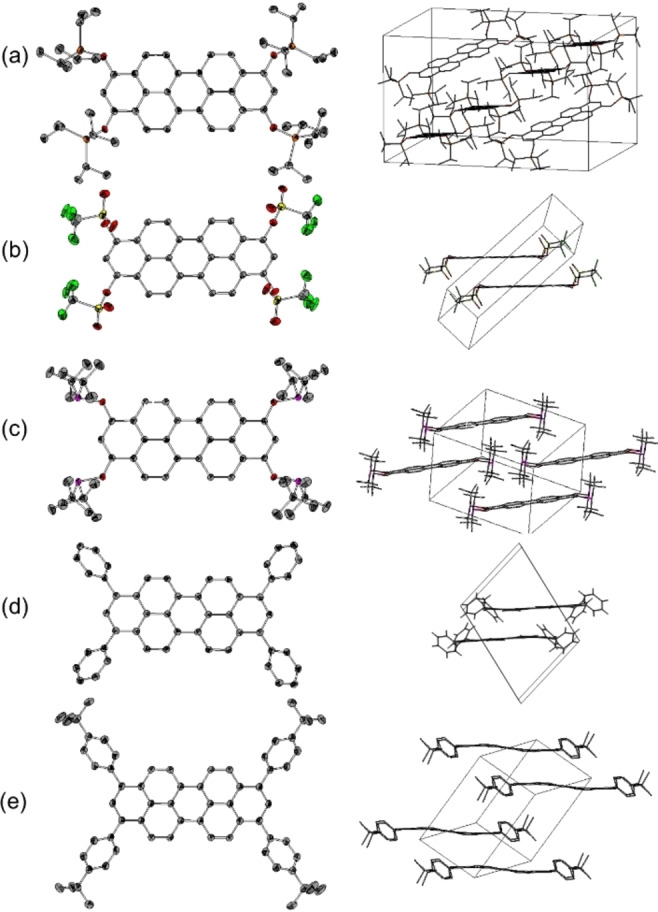
Molecular and lattice structures of (a) **3**, (b) **5**, (c) **6**, (d) **10** and (e) **11**. Ellipsoids are shown at 50 % level; all hydrogen atoms and solvent molecules are omitted for clarity.^[59]^

In order to study the molecular and lattice structure of 1,3,8,10‐tetrasubstituted peropyrenes with respect to their functional groups, single crystals were grown from a saturated *n*‐pentane solution at −20 °C (**6**) and via slow diffusion of *n*‐pentane vapor into saturated dichloromethane or chloroform solutions at room temperature (**3**, **5**, **10** and **11**). Figure 1 displays their molecular and crystal lattice structures in form of their unit cells.^[59]^ Silyl ether **3** crystallizes in monoclinic space group *P*2_1_/c with pairs of molecules arranged in a herringbone arrangement with 90° angles between different slipped stacks. All other compounds **5**, **6**, **10** and **11** crystallize in triclinic space group $P\bar{1}$ with a molecular and crystallographic inversion centre at the central peropyrene backbone defining symmetry related substituents in 1,3,8,10‐positions. Steric demand of triisopropyl silyl groups in **3** and bis‐*tert*‐butylphosphanyl substituents in **6** prevent their peropyrene backbones from any π‐stacking. Molecules rather pack in a staggered manner. In contrast, triflate‐substituted peropyrene **5** forms π‐stacked dimers with an intermolecular distance of 3.37 Å. Such dimers are also observed in phenyl substituted peropyrene **10** and *tert*‐butylphenyl substituted congener **11**, both with slightly larger intermolecular distances of 3.67 Å and 3.68 Å, respectively. The latter are induced by an out of plane twist of the phenyl groups with respect to the peropyrene plane: the dihedral angels are 53.4° and 50.6° for **10** and 55.5° and 49.5° for **11**.

The different substituents on the peropyrene backbone do not only influence the capability of π‐stacking but also the planarity of the peropyrene backbone measured as torsion angle of neighbouring naphthalene units. The twist is not at similar level as observed for substituents in bay positions.^[30]^

Little distortion is observed for triflate **5** (0.3°) and phosphinite **6** (0.7°). In triisopropyl silyloxy‐substituted **5** (2.5°) or phenyl‐substituted **10** (2.3°) the twist angle gets higher and reaches a maximum at *p*‐*tert‐*butylphenyl‐substituted **11** (4.9°). Only silyl ether **3** crystallizes in the same monoclinic space group *P*2_1_/c as unsubstituted peropyrene.^[22]^ The herringbone structural motif associated with parent peropyrene was identified to be responsible for a low energy gap between T_1_ and S_1_ states in crystalline state, thus for poor ability for singlet fission.[^15^, ^22^] In contrast, **5**, **6**, **10** and **11** crystallize in space group *P* $\bar{1}$ . Therefore, this particular substituent pattern provides a handle for peropyrene crystal engineering, a topic previously demonstrated for other substitution patterns of peropyrene.[^26^, ^28^]

### Optical Spectroscopy

**Figure 2:**
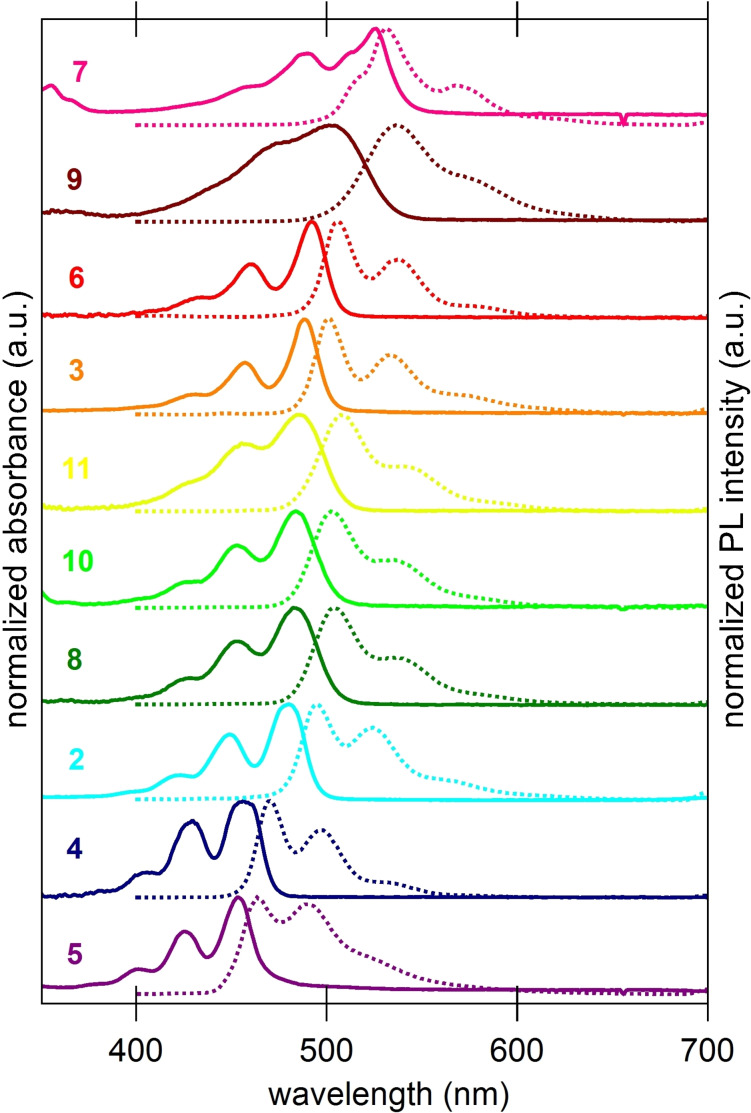
Normalized absorption and corresponding photoluminescence (PL) spectra (dashed) of 2–11 in dichloromethane.

The influence of different substituents in 1,3,8,10‐position on optical properties of 2–11 was investigated by UV/Vis and photoluminescence (PL) spectroscopy (Figure 2 and Table 1). All spectra were recorded in dichloromethane. A dependency of the energy of absorption and emission maxima from concentration in solution was not observed. Aggregation in solution tends to be small.

**Table 1:** Absorption and emission data of **2**–**11** ordered in decreasing HOMO‐LUMO gap.

Compound substituent R)	λ_max,abs._ (nm) [ϵ (10^−4^ L mol^−1^ cm^−1^)]	λ_max,em._ (nm) [Φ_PL_]	Stokes shift (eV)	E_g,opt_ (eV)^[a]^
**5** (OTf)	453 [3.42]	464 [0.02]	0.07	2.70
**4** (OPiv)	456 [5.55]	470 [0.02]	0.08	2.67
**2** (OTMS)	479 [4.29]	495 [0.30]	0.08	2.54
**8** ((CF_3_)_2_C_6_H_3_)	482 [1.11]	502 [0.52]	0.10	2.51
**10** (Ph)	483 [6.17]	500 [0.51]	0.09	2.52
**11** (*t*BuC_6_H_4_)	485 [9.19]	506 [0.66]	0.11	2.49
**3** (OTIPS)	488 [8.27]	500 [0.84]	0.06	2.51
**6** (OP(*t*Bu_3_)_2_)	492 [7.49]	506 [0.72]	0.07	2.48
**9** (2‐thienyl)	505 [7.12]	537 [0.25]	0.14	2.38
**7** (TMSA)	526 [4.85]	531 [0.37]	0.02	2.35

[a] Determined from the intersection wavelength of normalized absorption and emission spectra.

Compounds **2**–**11** also show pronounced vibronic progression fine structures with spacings of ∼30 nm, typical for the π*←π transition of other perylene derivatives[^60^, ^61^] and parent peropyrene.^[22]^ Only alkynyl substituted 7 and 2‐thienyl substituted 9 display a less distinct vibronic progression, probably due to aggregation effects. The emission bands are mirror images of the corresponding absorption bands, indicating similar molecular conformation of the electronic ground state and excited state.^[62]^ Triflate 5 is the only derivative displaying only weak fluorescence due to an overlay of the first vibronic progression mode with the fluorescence maximum. All other compounds show a qualitatively strong fluorescence. It seems that triflate groups trigger fluorescence quenching effects as it was discussed by Gade et al. for tetrabrominated tetraaza‐peropyrenes.^[63]^ Congeners **2**–**11** do show neither shifts of the UV‐Vis maxima in their concentration‐dependent UV‐Vis spectra (see Figures S1–S10) nor solvatochromic effects. Aryl substituted compounds **8**, **9** and **11**, in particular, display high molar attenuation coefficients (ϵ), whereas acceptor‐substituted **4** and **5** show lower values of ϵ. In general, the absorption and emission maxima are ranging over 72 nm and reflect the electron donating or withdrawing effects of the substituents: The electron‐poor triflate **5** (454 nm) displays most hypsochromically shifted, the electron‐rich trimethylsilyl alkynyl substituted 7 (526 nm) most bathochromically shifted maxima. The determined fluorescence quantum yields (Φ_PL_, determined by dilution method^[62]^ in DCM (see Supporting Information and Table 1) are high (up to 84 %) for electron‐rich compounds **3** and **6** and slightly lower for aryl‐substituted congeners (up to 66 %) owed supposedly due to higher reorganization energy losses.^[62]^ The quantum yields are as high as those of comparable diazaperopyrenes^[43]^ or twisted peropyrenes.^[31]^ Remarkably, fluorescence is nearly deactivated (Φ_PL_=2 %) for electron‐deficient substituents (**4** and **5**).

Our findings show that a precise fine tuning of absorption and emission maxima of peropyrenes is possible by substituent variation within the 1,3,8,10substitution pattern introduced here. Next to triflate **5**, pivaloate **4** is more hypsochromically shifted, whereas next to alkynyl derivative **7**, silylethers **2** and **3** and especially phosphinite **6** are more (∼25‐30 nm) bathochromically shifted.

**Figure 3:**
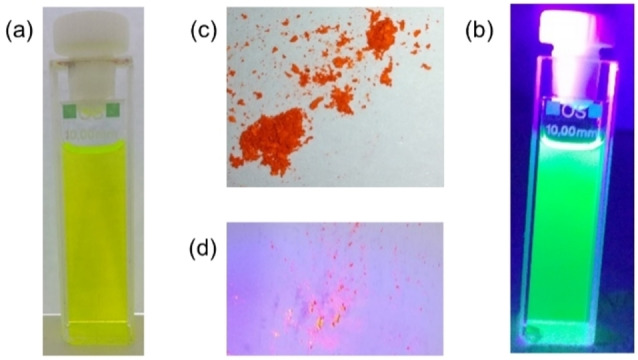
Photographs of peropyrene **8** in solution (∼10^−5^ M) under (a) ambient light; (b) under UV‐light (366 nm) and as powder under (c) ambient light and (d) under UV‐light (366 nm).

**Figure 4:**
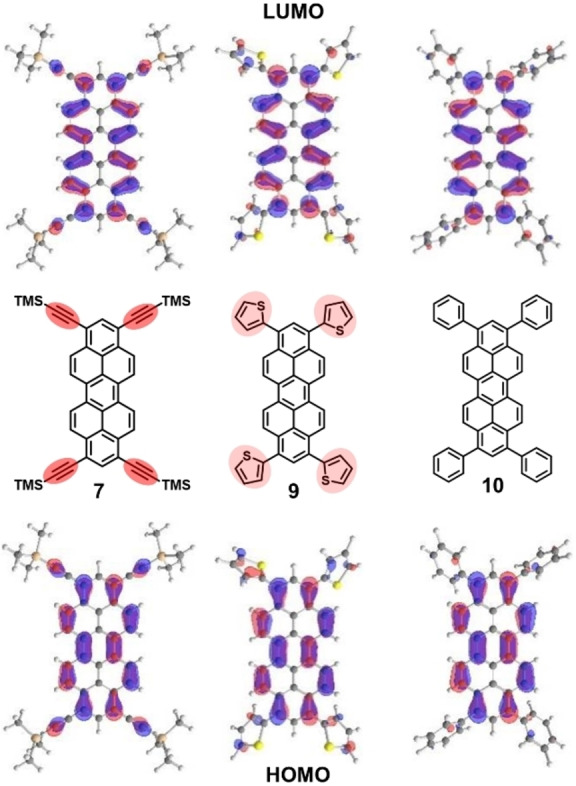
Molecular orbitals of **7** (left), **9** (centre) and **10** (right) (def2‐TZVPP/B3LYP level of theory, isoval. 0.03 a.u.) in comparison.

It is evident, that all three aryl substituted derivatives **8**, **10** and **11** display very similar absorption maxima around 483 nm, which leads to the conclusion that the nature of +I or ‐I substituents attached to the benzene ring, *p*‐*t*Bu (**11**) vs. 3,5‐CF_3_ (**8**) vs. H / none (**10**) does not influence the HOMO‐LUMO gap significantly (Figure 4). They are orange‐coloured solids and form yellow solutions with green fluorescence (Figure 3).

In contrast aryl substituents with +M effect such as 2‐thienyl groups in **9** lead to a significant bathochromic shift to 505 nm in the absorption. Not unexpectedly, conjugation to an electron‐rich π‐system such as thiophene in **7** (or alkyne in **9**) is triggering the HOMO‐LUMO gap more efficiently. In order to rationalize the individual influence of substituents in this respect, we calculated the ground state geometries of **2**–**11** at def2‐TZVPP/B3LYP level of theory (see Supporting Information, Tables S2–S11 for cartesian coordinates of the optimized geometries). The frontier Kohn‐Sham orbitals support our experimental observation that substituents in 1,3,8,10‐position influence the electronic properties. The HOMO and LUMO are delocalized all over the peropyrene core. However, the participation of substituent orbitals on both HOMO and LUMO coefficients particular at 1,3,8,10‐positions is increasing in the order Ph (**10**) <2‐thienyl (**7**) < alkynyl (**9**) (Figure 4) whereas no significant changes are observed for differently substituted phenyl substituents (**8**, **10** and **11**).

The trend in the absorption spectra is also reflected by TD‐DFT calculations at def2‐TZVPP/PBE level of theory assigning the absorption maxima mostly to HOMO‐LUMO (S_0_ to S_1_) transitions (see Figure S21 and Table S1 for details). The mirrored photoluminescence spectra are following the same trend with only small Stokes shifts (10‐20 nm) typically observed for rigid chromophores such as well‐studied perylene diimides.[^60^, ^61^] Thienyl substituted **8** displays a larger Stokes shift and broader absorption and emission bands indicating geometry changes between ground state and excited state, in other words a large reorganization energy. Contrastingly, alkyne derivative **7** displays a very small Stokes shift of 5 nm indicating similar molecular geometries in ground state and excited state with almost no energy loss by molecular reorganization between absorption and fluorescence. Similar observations were made for other alkynyl substituted aromatic systems.[^40^, ^63^] Finally, we approximated the magnitude of the optical energy gaps as the energy of the intersection wavelength of the normalized absorption and emission spectra (Table 1).^[64]^ Not surprisingly, the trend of the optical gap narrowing in the range 2.70 eV for **5** to 2.38 eV for **7** follows previously discussed trend in bathochromically shifted absorption and emission spectra. This trend is in accord with peropyrenes functionalized only in 1‐ and 8‐position.[^35^, ^36^]

### Electrochemistry and theoretical modelling of the frontier orbital energies

**Figure 5:**
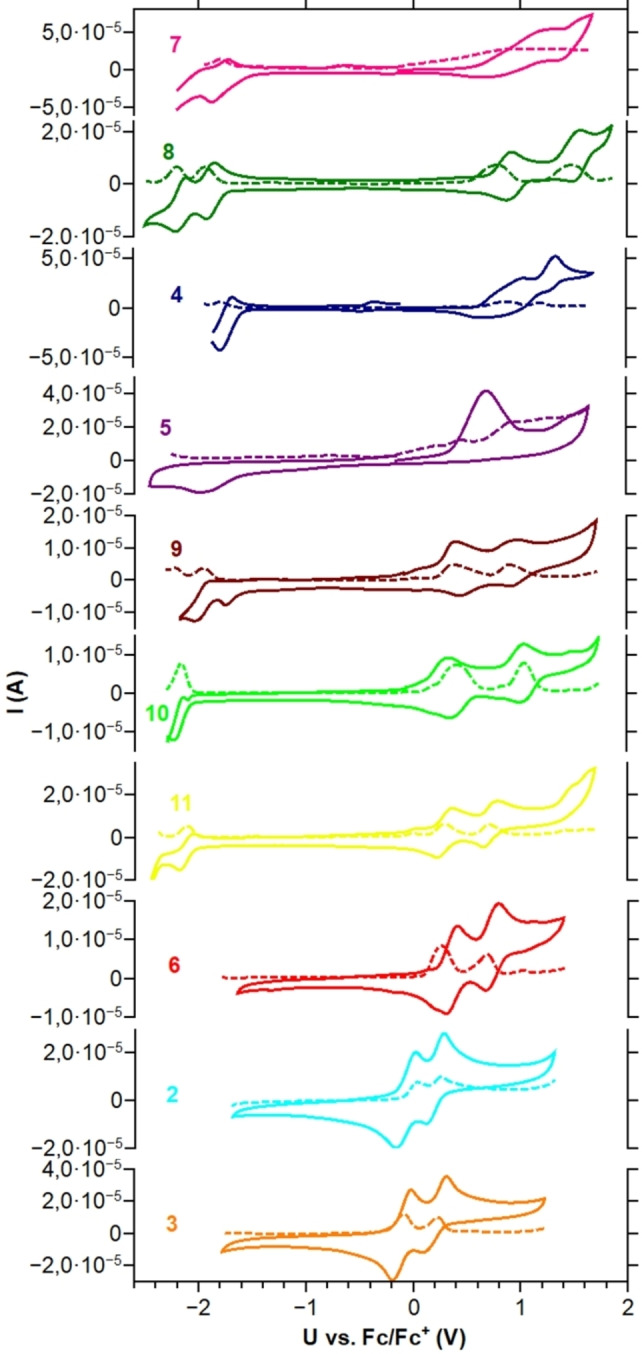
Cyclic voltammograms of **2**–**11** (measured in CH_2_Cl_2_, 0.1 M *n*‐Bu_4_NPF_6_, 50 mV s^−1^ scan rate, glassy carbon electrode) and their corresponding differential pulse voltammograms (DPV, dashed lines, 10 mV s^−1^ scan rate).

Electrochemical properties of these 1,3,8,10‐substituted peropyrenes were investigated by cyclic voltammetry (CV) and differential pulse voltammetry (DPV) vs. ferrocene as internal standard (Figure 5 and Table 2, Figures S13–S22). Only triflate **5** and pivaloate **4** showed irreversible redox behaviour and decomposition during CV data acquisition under the defined parameters. All aryl substituted derivatives **8**–**11** display two almost reversible oxidation waves and one reversible reduction wave underlining their electron rich PAH character. In addition, **8** and **9** display a second quasi reversible reduction wave. As expected, 3,5‐CF_3_‐substituted aryl derivative **8** is the easiest to be reduced and the most difficult to be oxidized, whereas phenylogue *tert*‐butyl derivative **11** is easiest to be oxidized and most difficult to be reduced. We were surprized to find, that thienyl substituted heteroaryl congener **9** tends to be in the middle range, less easily oxidized and less easily reduced than just mentioned phenyl derivatives tuned to only one extreme (Figure 2). The accessibility of first oxidation and reduction potentials in this arene substituent series observable as metastable radical anion and radical cation species allows to discuss a narrowing of the electrochemical window within the aryl substituted species **8** (2.77 eV) up to **9** (2.37 eV) as it is also the case for the optically determined energy gaps.

**Table 2:** Electrochemical Data of **2**–**11**.

Compound	E_1/2,Red1_ (V)	E_1/2,Ox1_ (V)	E_1/2,Ox2_ (V)	E_HOMO,exp_ (eV)^[a]^	E_LUMO,exp_ (eV)^[b]^
**3** (OTIPS)	–	−0.11	0.21	−4.69	−2.18
**2** (OTMS)	–	−0.07	0.19	−4.73	−2.19
**6** (OP(*t*Bu_3_)_2_)	–	0.23	0.61	−5.03	−2.55
**11** (*t*BuC_6_H_4_)	−2.12	0.29	0.73	−5.09	−2.60
**10** (Ph)	−2.18	0.34	1.00	−5.14	−2.62
**9** (2‐thienyl)	−1.94^[c]^	0.43	0.94	−5.23	−2.85
**5** (OTf)	≈−1.9	≈−0.7	–	−5.50	−2.80
**4** (OPiv)	−1.73	0.83	1.30	−5.63	−2.93
**8** ((CF_3_)_2_C_6_H_3_)	−1.88^[d]^	0.90	1.53	−5.70	−3.19
**7** (TMSA)	−1.81	0.99	–	−5.79	−3.44

[a] Determined using literature methods by referencing on Fc/Fc^+^ as internal standard (E_HOMO_ (fc)=‐4.8 eV) added after data acquisition: E_HOMO,exp_=−4.8 eV−E_1/2,ox1._ [b] Determined with help of the optical gap (E_g,opt_, see Table 1): E_LUMO,exp_=E_HOMO,exp_+E_g,opt_. [c] E_1/2,Red2_=−2.20 V. [d] E_1/2,Red2_=−2.17 V.

Beyond the limits of these aryl derivatives first oxidation potentials are ranging from −0.11 V for TIPS‐substituted derivative **3**, the easiest to be oxidized in this series and 0.99 V for alkynyl derivative **7**, the most difficult to be oxidized. All representatives with −OSiR_3_ (**2**, **3**) and −OPR_2_ (**6**) functional groups do not display a reversible reduction wave within the electrochemical window of dichloromethane, but two pronounced oxidation waves. This reflects their low electron affinities and also their oxidation (air) sensitivity. In contrast to that, electron‐poor tetrapivaloate **4** and tetratriflate **5** show one reduction potential due to the electron affinity of their electron‐withdrawing pivaloate or triflate groups. Alkynyl congener **7** represents not only a species most difficult to be oxidized (E_1/2,Ox1_=0.99 V) but also nearly the easiest to be reduced (E_1/2,Red1_=−1.81 V). **7** shows also the narrowest optical energy gap according to UV‐Vis and PL measurements.

In order to relate experiment and theory, experimental HOMO energies were registered from the first oxidation potential determined by differential pulse voltammetry (DPV) vs. Fc/Fc^+^ assuming an ionization potential of 4.8 eV for ferrocene^[65]^ (Table 2).

**Figure 6:**
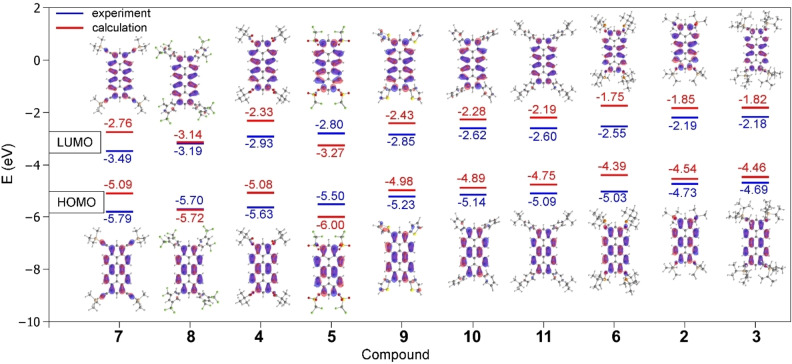
Energy diagram of experimental HOMO and LUMO energies of **2**–**11** determined via HOMO from first oxidation potential vs. Fc/Fc^+^ from CV/DPV and HOMO‐LUMO gap from UV‐Vis measurements in comparison to calculated HOMO and LUMO energies of **2**–**11** (def2‐TZVPP/B3LYP). The corresponding Kohn‐Sham orbitals of HOMO and LUMO of **2**–**11** are shown as insets (isoval. 0.03 a.u.).

The experimental LUMO energy was determined with help of the optical gap energies determined from the UV‐Vis spectra (Table 1) and the HOMO energies. Both were compared to the calculated orbital energies. Def2‐TZVPP/B3LYP reproduced the experimentally determined frontier molecular energies best. The experimental HOMO levels range from −5.70 eV for alkyne **7** to −4.69 eV for electron‐rich silyl ether **3**. But also other peropyrenes with electron‐deficient substituents such as **4**, **5** or **8** show HOMO energies below −5.5 eV corresponding to high first oxidation potentials. The LUMO energies vary from −3.44 eV for **7** to −2.18 eV for **3**, respectively. The high lying HOMOs of **2** and **6** go also in hand with their air sensitivity. We conclude that relatively large orbital coefficients at peropyrene 1,3,8,10‐positions allow to tune absolute LUMO and HOMO energies as well as their energy gap by the electronic character of introduced substituents (Figure 6). Namely, electron‐withdrawing substituents such as pivaloate (**4**) or 3,5‐bis(trifluoromethyl)phenyl (**8**) lead to low‐lying frontier orbitals, whereas electron‐donating substituents such as TIPS−O (**3**), TMS−O (**2**) and 2‐thienyl (**9**) lead to energetically high‐lying frontier orbitals. However, as observed for highly reactive triflate **5**, low lying frontier molecular orbitals do not automatically guarantee any stability of electrochemically generated radical anions (or cations) under ambient conditions.

The experimental HOMO and LUMO values are in accord with values of alkoxy‐substituted peropyrenes which were proven to be hole‐transporting semiconductors.[^35^, ^36^] The calculated HOMO and LUMO energies and calculated HOMO‐LUMO energy gap also follow this trend and are compared with experimental ones in Figure 5. Especially the calculated energy gap is in good agreement with the experimental values. Calculated absolute HOMO and LUMO energy values lie generally higher than experimental ones. Best agreement between experimental and calculated frontier MO energies is obtained for aryl peropyrenes **8**, **9** and **10** as well as for silyl ethers **2** and **3**.

### Effect of additional 2‐ and 9‐substituents

**Scheme 5:**
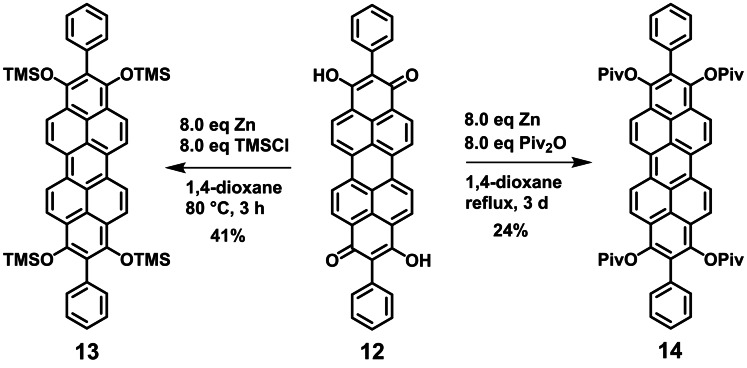
Synthesis of peropyrenes **13** and **14** substituted and reductively functionalized in 1,2,3,8,9,10‐positions.

In order to study the influence of substituents in terminal 2,9‐positions, we introduced phenyl groups by simply following the reductive functionalization and aromatization strategy at 2,9‐bisphenyl peropyrene hydroxyquinone **12** synthesized analogously to terminal unsubstituted peropyrene hydroxyquinone **2**. Two model compounds, tetrasilyl ether **13** and tetrapivaloate **14**, were synthesized applying our protocol with zinc as reducing agent (Scheme 5). The syntheses proceeded with slightly lower yields than in case of their congeners **2** and **4** without phenyl substituents. Again, pivaloate **14** turned out to be air stable whereas silyl ether **13** hydrolyses slowly on air. The solubility of the sterically more crowded and rigid compounds **13** and **14** is significantly lower than in the case of **2** and **4**.

**Figure 7:**
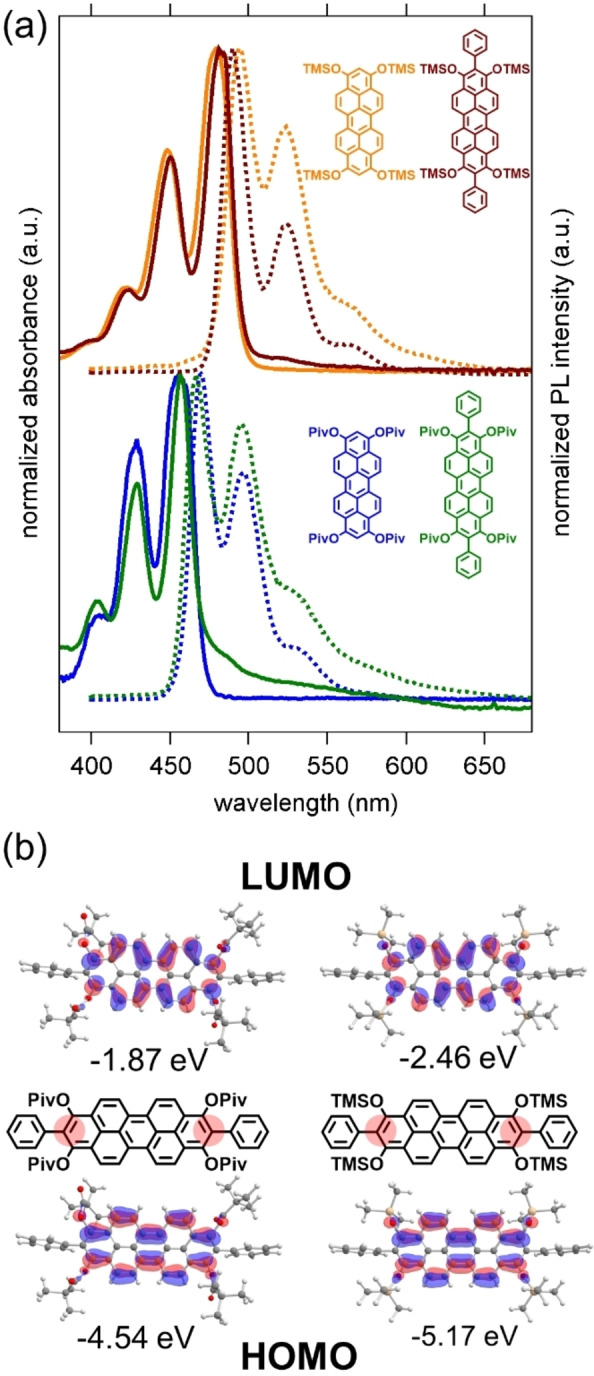
(a) Comparison of absorption and photoluminescence spectra (dashed lines) of **2** and **13** and **4** and **14** recorded in DCM; (b) Kohn‐Sham orbitals of HOMO and LUMO of **13** and **14** and their energies calculated by DFT (def2‐TZVPP/B3LYP level of theory).

A comparison of absorption and emission spectra of silyl ethers **2** and **13** or pivaloates **4** and **14** reveals, that absorption and emission maxima, thus the HOMO‐LUMO gap are essentially the same. Additional phenyl substituents at 2‐ and 9‐positions do not drastically change optical properties (Figure 7 (a)). Only the Stokes shift is slightly smaller in **13** and **14** than in unsubstituted pendants **2** and **4**. This is indicating a higher rigidity of the peropyrenes **13** and **14** substituted in 2,9‐position. The nearly identical absorption and emission spectra can be rationalized by DFT calculations at def2‐TZVPP/B3LYP level of theory (Tables S12 and S13). The optimized geometries of **13** and **14** show that the phenyl groups in 2,9‐position are oriented nearly perpendicular with respect to the molecular plane of the peropyrene backbone, conjugative effects are not expected. Furthermore, the orbital coefficients at ring positions 2 and 9 are small, so that substituents at these positions do not affect and contribute significantly to the HOMO and LUMO (Figure 7 (b)), thus to the HOMO→LUMO transition. The calculated HOMO and LUMO energies of **13** and **14** also differ only slightly from those of **2** and **4**. These experimental and computational findings let us conclude that variation of the terminal 2‐ and 9‐ position is much less effective for fine tuning optical properties than variations at 1,3,8,10‐positions.

## Conclusions

We presented a versatile access to 1,3,8,10‐ and 1,2,3,8,9,10‐functionalized peropyrenes. Four siloxy groups (**2**, **3**), pivaloyl (**4**) and triflyl groups (**5**) were introduced via Zn mediated reductive aromatization and functionalization of readily available quinoid precursors. Another strategy uses *n*‐butyl lithium as base and reducing agent in order to successfully introduce four phosphinite groups (**6**). Triflyl and pivaloyl substituted peropyrenes **4** and **5** were applied in post‐functionalization via Pd‐ and Ni‐catalysed cross‐coupling reactions leading to uncommon tetraaryl and tetraalkynyl peropyrenes (**8**‐**11** and **7**). The electron‐donating or withdrawing functional groups did not only influence the π‐stacking in the lattice of XRD structurally characterized representatives **3**, **5**, **6**, and **10** but also the absolute energy of frontier orbitals and HOMO‐LUMO gap extracted from experimental UV‐Vis, photoluminescence and cyclic voltammetry measurements: Absorption maxima were ranging from 426 nm to 526 nm, while HOMO and LUMO energies were varying from −5.66 eV to −4.66 eV and −2.92 eV to −2.12 eV, respectively. DFT calculations support these experimental trends. Our synthetic strategy allows to tailor the optoelectronic properties of peropyrenes for applications such as in hole‐conducting semiconductors or fluorescence emitters.

## Conflict of interest

The authors declare no conflict of interest.

## Supporting information

As a service to our authors and readers, this journal provides supporting information supplied by the authors. Such materials are peer reviewed and may be re‐organized for online delivery, but are not copy‐edited or typeset. Technical support issues arising from supporting information (other than missing files) should be addressed to the authors.

Supporting Information
