# Supplementary material for: Tetrasubstituted Peropyrenes Formed by Reductive Aromatization: Synthesis, Functionalization and Characterization
Source: Chemistry. 2021 Jun 15;27(43):11065–75. doi: 10.1002/chem.202101101 (PMC8453513; doi:10.1002/chem.202101101)
Supplement: Supplementary file 1 — Supporting Information [file CHEM-27-11065-s001.pdf]

Chemistry–A European Journal

Supporting Information

Tetrasubstituted Peropyrenes Formed by Reductive Aromatization: Synthesis, Functionalization and Characterization

Simon Werner, Tobias Vollgraff, and Jörg Sundermeyer*

Contents

Experimental Section **1**

Methods and devices 1

Synthesis 1

Concentration-dependent UV-Vis spectroscopy **5**

Determination of fluorescence quantum yields **8**

Additional CV spectra **9**

TD-DFT results **13**

Optimized geometries (DFT) **15**

NMR Spectra **35**

*1,3,8,10-tetrakis((trimethylsilyl)oxydibenzo[cd,lm]-perylene (**2**)* 35

*1,3,8,10-tetrakis((triisopropylsilyl)oxy)dibenzo[cd,lm]-perylene (**3**)* 36

*Dibenzo[cd,lm]perylene-1,3,8,10-tetrayltetrakis(2,2-dimethyl-propanoate) (**4**)* 37

*Dibenzo[cd,lm]perylene-1,3,8,10-tetrayl tetrakis-(trifluoromethanesulfonate) (**5**)* 38

*Dibenzo[cd,lm]perylene-1,3,8,10-tetrayl tetrakis-(trifluoromethanesulfonate) (**6**)* 39

*1,3,8,10-tetrakis((trimethylsilyl)ethynyl)dibenzo[cd,lm]-perylene (**7**)* 41

*1,3,8,10-tetrakis(3,5-bis(trifluoromethyl)phenyl)dibenzo-[cd,lm]perylene (**8**)* 42

*1,3,8,10-tetra(thiophen-2-yl)dibenzo[cd,lm]perylene (**9**)* 43

*1,3,8,10-tetraphenyldibenzo[cd,lm]perylene (**10**)* 44

*1,3,8,10-tetraphenyldibenzo[cd,lm]perylene (**11**)* 45

*((2,9-diphenyldibenzo[cd,lm]perylene-1,3,8,10-tetrayl)tetrakis(oxy))tetrakis-(trimethylsilane) (**13**)* 46

*2,9-diphenyldibenzo[cd,lm]perylene-1,3,8,10-tetrayl tetrakis(2,2-dimethyl-propanoate) (**14**)* 47

Single crystal X-ray structures **48**

Crystal Data *48*

Molecular structures of the title compounds *50*

References **64**

Experimental Section

Methods and devices

All preparative operations were conducted by using standard Schlenk techniques and solvents were dried according to common procedures[1] and passed through columns of aluminium oxide, 3 Å molecular sieves and R3-11G-catalyst (BASF) or stored over molecular sieves (3 Å or 4 Å). Peropyrenequinone (**1**)[2,3] and 3-hydroxy-2-phenyl-1*H*-phenalen-1-one[4] were synthesized according to literature method.

The XRD data collection was performed on a Stoe Stadivari diffractometer or a Bruker D8 Quest diffractometer by the XRD analytic department of Fachbereich Chemie, Universität Marburg. Information concerning the used hardware, and software used for data collection, cell refinement and data reduction as well as structure refinement can be reviewed in the electronic supplement tables and CCDC 2072185–2072189. After solution (SHELXT)[5] and refinement process (SHELXL 2017/1)[6] the data were validated by using Platon.[7] All graphic representations were created with Diamond 4.[8]

^{1}H and proton decoupled ^{13}C NMR spectra were recorded in automation or by the NMR service of Fachbereich Chemie with a Bruker Avance II 300 spectrometer, a Bruker Avance II HD 300, DRX 400 or Avance III 500 spectrometer. All spectra were recorded at ambient temperature. ^{1}H and ^{13}C NMR spectra were calibrated using residual proton signals of the solvent (CD_2Cl_2: δ_H = 5.32 ppm, δ_C = 53.84 ppm, $CDCl_3$: δ_H = 7.26 ppm, δ_C = 77.16 ppm). Multiplicity is abbreviated as follows: s (singlet), d (doublet), t (triplet), q (quartet), qt (quintet), m (multiplet), br (broad). HR-APCI mass spectra were acquired with a LTQ-FT Ultra mass spectrometer (Thermo Fischer Scientific). The resolution was set to 100.000. HR-EI mass spectra were acquired with an AccuTOF GCv 4G (JEOL) Time of Flight (TOF) mass spectrometer. An internal or external standard was used for drift time correction. The LIFDI ion source and FD-emitters were purchased from Linden ChroMasSpec GmbH (Bremen, Germany). IR spectra were recorded in a glovebox on a Bruker Alpha ATR-FT-IR spectrometer.

Absorption spectra were recorded with a Varian Cary-5000 UV/Vis/NIR spectrophotometer in 10 mm cuvettes in dichloromethane with concentrations of 10 μM with a scan rate of 600 nm/min. Emission spectra were recorded with a Varian Cary Eclipse Spectrophotometer in 10 nm cuvettes in dichloromethane with a scan rate of 600 nm/min. Cyclic voltammetry (CV) and differential pulse voltammetry (DPV) measurements were carried out on a rhd instruments TSC 1600 closed electrochemical workstation under nitrogen atmosphere in a glovebox (Labmaster 130, MBraun); working electrode: glassy carbon; counter electrode: platinum crucible; reference electrode: platinum wire pseudo reference electrode. The samples were measured in dichloromethane and calibrated using ferrocene as internal standard added in the final sweep after CV data collection. Dichloromethane was filtered through an aluminum oxide pad prior to use. Tetrabutylammonium hexafluorophosphate ($TBAPF_6$; >99.0 %) was used as electrolyte for electrochemical analysis. The measurements were carried out at a concentration of 100 mmol/L of electrolyte.

Density functional theory (DFT) calculations using the B3LYP[9–11] functional were performed. The def2-TZVPP[12–14] basis set was used with RIJDX auxiliary base set[15,16], employing the resolution-of-identity approximation.[17,18] Further D3-dispersion correction[19] was considered by applying Becke−Johnson damping.[20–23] Structural optimizations and TD-DFT calculations were performed using Orca 3.0.3.[24] The atomic coordinates for geometry optimization were taken from XRD structures, if possible. The structurally optimized molecules were used for TD-DFT calculations using the PBE[25,26] functional (PBE0-D3/def2-TZVPP), employing the resolution-of-identity approximation for both Coulomb integrals and HF exchange integrals.[18]

Synthesis

Synthesis of 1,3,8,10-tetrakis((trimethylsilyl)oxydibenzo[*cd,lm*]-perylene (2). Peropyrenequinone (**1**, 778 mg, 2.0 mmol, 1.0 eq), Zn dust (1.05 g, 16.0 mmol, 8.0 eq) and trimethylsilyl chloride (2.0 mL, 16.0 mmol, 8.0 eq) were stirred for 3 h under argon atmosphere at 100 °C in 40 mL 1,4-dioxane. The greenish solution was cooled to room temperature and all volatile components were removed in vacuo. The residue was taken up in 40 mL of dichloromethane and filtered. After removing the solvent under vacuum, the crude product was washed with *n*-pentane. 647 mg (0.95 mmol, 48%) of **2** were obtained as an orange solid. ^{1}H NMR (300.1 MHz, $CDCl_3$): δ_H = 0.47 (s, 36H, Si(C*H*$_3$)$_3$), 7.42 (s, 2H, *H*2, *H*9), 8.47 (d, $^{3}J_{H,H}$ = 9.4 Hz, 4H, *H*4, *H*7, *H*11, *H*14), 8.99 (d, $^{3}J_{H,H}$ = 9.6 Hz, 4H, *H*5, *H*6, *H*12, *H*13) ppm. ^{13}C NMR (75.5 MHz, $CDCl_3$): δ_C = 0.7, 108.2, 118.1, 119.5, 120.8, 123.4, 124.9, 127.3, 149.4 ppm. HR-MS (EI+) m/z clcd. for $[C_{36}H_{46}O_4Si_4]^+$: 678.24409 (found: 678.24731). IR (ATR), ṽ = 3072 (m), 2954 (w), 2864 (w), 1624 (w), 1596 (w), 1554 (m), 1504 (m), 1482 (w), 1423 (w),1393 (w), 1352 (w), 1292 (w), 1251 (m), 1190 (m), 1167 (s), 1147 (w), 1089 (w), 915 (w), 873 (m), 838 (m), 785 (w), 749 (w), 681 (w), 645 (w), 559 (w) cm^{-1}. λ_{max} (abs.) = 479 nm (DCM, ε = 4.29·10^{-4} L·mol^{-1}·cm^{-1}). λ_{max} (em.) = 495 nm (DCM). CV: $E_{1/2}(Ox_2)$ = 0.19 V (vs. Fc/Fc$^+$), $E_{1/2}(Ox_1)$ = - 0.07 V (vs. Fc/Fc$^+$).

Synthesis of 1,3,8,10-tetrakis((triisopropylsilyl)oxy)dibenzo[*cd*,*lm*]-perylene (3). Peropyrenequinone (**1**, 389 mg, 1.0 mmol, 1.0 eq), Zn dust (503 mg, 8.0 mmol, 8.0 eq), imidazole (545 mg, 8.0 mmol, 8.0 eq) and triisopropyl chloride (1.7 mL, 8.0 mmol, 8.0 eq) were refluxed for 18 h under argon atmosphere in 20 mL 1,4-dioxane. The brownish green solution was cooled to room temperature, filtered over a pad of neutral aluminium oxide and washed with 50 mL dichloromethane, subsequently. After removing the solvent under vacuum, the crude product was washed with 20 mL methanol and 20 mL *n*-pentane and dried in vacuo. 203 mg (0.20 mmol, 20%) of an orange solid were obtained after drying in vacuo. X-ray suited single crystals could be obtained by layering a saturated solution of **3** in dichloromethane with *n*-pentane at –18 °C. ^{1}H NMR (300.1 MHz, CD_2Cl_2): δ_H = 1.25 (d, $^{3}J_{H,H}$ = 7.4 Hz, 72H, $(CH_3)_2$C*H*), 1.54 (hept, $^{3}J_{H,H}$ = 7.6 Hz, 12H, (C*H*$_3$)$_2$CH), 7.24 (s, 2H, *H*2, *H*9), 8.60 (d, $^{3}J_{H,H}$ = 8.1 Hz, 4H, *H*4, *H*7, *H*11, *H*14), 8.97 (d, $^{3}J_{H,H}$ = 9.5 Hz, 4H, *H*5, *H*6, *H*12, *H*13) ppm. ^{13}C NMR (75.5 MHz, CD_2Cl_2): δ_C = 13.8, 18.4, 107.9, 118.3, 120.1, 121.9, 125.8, 128.3, 151.5 ppm. HR-MS (LIFDI+) m/z clcd. for $[C_{52}H_{94}O_4Si_4]^+$: 1014.62291 (found: 1014.62294). IR (ATR), ṽ = 2940 (m), 2862 (m), 1592 (w), 1499 (w), 1461 (m), 1351 (w), 1287 (vs), 1189 (m), 1086 (m), 1002 (w), 971 (vs), 824 (s), 781 (vs), 718 (w), 633 (vs), 582 (w) cm^{-1}. λ_{max} (abs.) = 488 nm (DCM, ε = 8.27·10^{-4} L·mol^{-1}·cm^{-1}). λ_{max} (em.) = 500 nm (DCM). CV: $E_{1/2}(Ox_2)$ = 0.21 V (vs. Fc/Fc$^+$), $E_{1/2}(Ox_1)$ = - 0.11 V (vs. Fc/Fc$^+$).

Synthesis of dibenzo[*cd*,*lm*]perylene-1,3,8,10-tetrayltetrakis(2,2-dimethyl-propanoate) (4). Peropyrenequinone (**1**, 3.80 g, 9.8 mmol, 1.0 eq), Zn dust (5.18 g, 78.0 mmol, 8.0 eq) and pivalic anhydride (15.9 mL, 78.0 mmol, 8.0 eq) were refluxed for 3 d under argon atmosphere in 200 mL 1,4-dioxane. The brownish green solution was cooled to room temperature, filtered over a pad of neutral aluminium oxide and washed with 200 mL dichloromethane, subsequently. After removing the solvent in fine vacuum, the crude product was suspended in 200 mL *n*-pentane and filtered. 2.43 g (3.35 mmol, 34%) of the brown solid **4** were obtained after drying in vacuo. ^{1}H NMR (300.1 MHz, CD_2Cl_2): δ_H = 1.63 (s, 36H, C(C*H*$_3$)$_3$), 7.69 (s, 2H, *H*2, *H*9), 8.13 (d, $^{3}J_{H,H}$ = 9.4 Hz, 4H, *H*4, *H*7, *H*11, *H*14), 8.87 (d, $^{3}J_{H,H}$ = 9.6 Hz, 4H, *H*5, *H*6, *H*12, *H*13) ppm. ^{13}C NMR (75.5 MHz, $CDCl_3$): δ_C = 27.7, 39.9, 114.7, 120.5, 121.9, 123.0, 125.7, 126.5, 144.6, 177.2 ppm. HR-MS (APCI+) m/z clcd. for $[C_{46}H_{47}O_4]^+$: 727.3265 (found: 727.3272). IR (ATR), ṽ = 2964 (m), 2873 (w), 1751 (s), 1603 (w), 1480 (w), 1422 (w), 1395 (w), 1296 (m), 1173 (w), 1112 (vs), 1029 (w), 941 (w), 897 (w), 787 (w), 759 (w) cm^{-1}. λ_{max} (abs.) = 456 nm (DCM, ε = 5.55·10^{-4} L·mol^{-1}·cm^{-1}). λ_{max} (em.) = 470 nm (DCM). CV: $E_{1/2}(Ox_2)$ = 1.30 V, $E_{1/2}(Ox_1)$ = 0.82 V, $E_{1/2}(Red_1)$ = -1.73 V.

Synthesis of dibenzo[*cd*,*lm*]perylene-1,3,8,10-tetrayltetrakis-(trifluoromethanesulfonate) (5). Route A: Peropyrenequinone (**1**, 184 mg, 0.5 mmol, 1.0 eq) and Zn dust (262 mg, 4.0 mmol, 8.0 eq) were suspended in 20 mL diethyl ether and trimethylsilyl chloride (0.5 mL, 4.0 mmol, 8.0 eq) were added dropwise. After cooling to 0 °C, trifluoromethanesulfonic anhydride (1.2 mL, 2.0 mmol, 4.0 eq) was added dropwise over 30 min. The reaction solution was stirred at room temperature for 2 d. After removing the solvent under vacuum, the crude product was dissolved in 20 mL dichloromethane, filtered and the solvent removed under reduced pressure. The residue was washed with *n*-pentane and dried. 110 mg (0.12 mmol, 23%) of **5** were obtained as brown solid.

Route B: 475 mg (0.7 mmol, 1.0 eq) of **2** were suspended in 100 mL diethyl ether and cooled to 0 °C. Over a period of 30 min, 1.3 mL (3.1 mmol, 4.4 eq) *n*-butyl lithium (2.43 M, *n*-hexane) were added dropwise. The purple suspension was stirred for 2 h at room temperature, cooled down to 0 °C and trifluoromethanesulfonic anhydride (1.9 mL, 3.1 mmol, 4.4 eq) was added dropwise over 30 min. After stirring at room temperature for 3 h, **5** was precipitated from the brownish reaction mixture at –18 °C, filtered and dried in vacuo after washing with *n*-pentane and diethyl ether. 440 mg (0.49 mmol, 70%) of **5** were obtained. X-ray suited single crystals were obtained by slow diffusion of *n*-pentane into a saturated solution of **5** in dichloromethane. Note: due to the instability of **5**, decomposition products could not be removed completely and therefore it was used for the next steps directly without further purification. ^{1}H NMR (300.1 MHz, $CDCl_3$): δ_H = 8.21 (s, 2H, *H*2, *H*9), 8.71 (d, $^{3}J_{H,H}$ = 9.5 Hz, 4H, *H*4, *H*7, *H*11, *H*14), 9.50 (d, $^{3}J_{H,H}$ = 9.6 Hz, 4H, *H*5, *H*6, *H*12, *H*13) ppm. ^{13}C NMR (125.8 MHz, $CDCl_3$): δ_C = 113.8, 117.7, 120.3, 120.7, 124.2, 125.8, 126.2, 126.5, 141.9 ppm. ^{19}F NMR (235.3 MHz, $CDCl_3$): δ_H = -72.6 (s) ppm. HR-MS (LIFDI+) m/z clcd. for $[C_{30}H_{10}F_{12}O_{12}S_4]^+$: 917.88635 (found: 917.88694). IR (ATR), ṽ = 2932 (w), 2232 (w), 2027 (w), 1600 (w), 1423 (m), 1302 (m), 1284 (m), 1236 (vs), 1180 (m), 1136 (m), 1092 (m), 1025 (m), 942 (w), 900 (w), 791 (w), 723 (w), 699 (m), 619 (w), 523 (w) cm^{-1}. λ_{max} (abs.) = 454 nm (DCM, ε = 3.42·10^{-4} L·mol^{-1}·cm^{-1}). λ_{max} (em.) = 494 nm (DCM), CV: $E_{1/2}(Ox_1)$ ≈ -0.7 V (vs. Fc/Fc$^+$), $E_{1/2}(Red_1)$ ≈ -1.9 V.

Synthesis of 1,3,8,10-tetrakis((di-*tert*-butylphosphaneyl)oxy)dibenzo-[*cd*,*lm*]perylene (6). Peropyrenequinone (**1**, 776 mg, 2.0 mmol, 1.0 eq) was suspended in THF (40 mL). At room temperature, 4.1 mL (10.0 mmol, 5.0 eq) *n*-butyl lithium (2.43 M, *n*-hexane) were added dropwise. The purple reaction mixture was stirred for 2 h at 50 °C, until the gas evolution ceased. After cooling to room temperature, 2.45 mL (12.8 mmol, 6.5 eq) di-*tert*-butylchlorophosphine were added and the mixture was refluxed for 18 h. After cooling to room temperature, the solvent was removed in fine vacuum. The crude product was dissolved in 60 mL dichloromethane and filtered. After evaporating the solvent under reduced pressure, the brown residue was washed with *n*-pentane. 463 mg (0.48 mmol, 24%) of the brown solid **6** were obtained after drying in vacuo. Single crystals suitable for XRD measurements were obtained from a saturated solution of **6** in *n*-pentane at -18 °C. ^{1}H NMR (300.1 MHz, $CDCl_3$): δ_H = 1.33 (d, 72H, $^{3}J_{P,H}$ = 11.8 Hz, P(C(C*H*$_3$)$_3$)$_2$), 8.52 (t, 2H, $^{4}J_{P,H}$ = 5.2 Hz, *H*2, *H*9), 8.67 (d, $^{3}J_{H,H}$ = 9.4 Hz, 4H, *H*4, *H*7, *H*11, *H*14), 8.98 (d, $^{3}J_{H,H}$ = 9.6 Hz, 4H, *H*5, *H*6, *H*12, *H*13) ppm. ^{31}P NMR (101 MHz, $CDCl_3$): δ_P = -151.7 (s) ppm. ^{13}C NMR (75.5 MHz, $CDCl_3$): δ_C = 27.7 (d, $^{2}J_{P,C}$ = 15.6 Hz), 36.3 (d, $^{1}J_{P,C}$ = 26.7 Hz), 103.6, 116.5, 120.0, 121.0, 125.6, 128.0, 154.3 (d, $^{3}J_{P,C}$ = 10.8 Hz) ppm. HR-MS (LIFDI+) m/z clcd. for $[C_{58}H_{82}O_4P_4]^+$: 966.51635 (found: 966.51486). IR (ATR), ṽ = 2941 (m), 2896 (w), 2863 (w), 1624 (w), 1595 (m), 1559 (w), 1497 (m), 1473 (w), 1389 (w), 1275 (vs), 1179 (m), 1130 (m), 1075 (m), 1011 (w), 958 (m), 853 (w), 772 (s), 707 (w), 679 (w), 635 (w), 612 (w), 557 (w) cm^{-1}. λ_{max} (abs.) = 492 nm (DCM, ε = 7.49·10^{-4} L·mol^{-1}·cm^{-1}). λ_{max} (em.) = 506 nm (DCM). CV: $E_{1/2}(Ox_2)$ = 0.60 V, $E_{1/2}(Ox_1)$ = 0.23 V (vs. Fc/Fc$^+$).

Synthesis of 1,3,8,10-tetrakis((trimethylsilyl)ethynyl)dibenzo[*cd*,*lm*]-perylene (7). Triflate **5** (194 mg, 0.2 mmol, 1.0 eq) and trimethylsilyl acetylene (0.25 mL, 1.6 mmol, 8.0 eq) were dissolved in THF (6 mL) and triethylamine (2 mL), copper iodide (8 mg, 0.04 mmol, 20 mol%) and [Pd(dppf)Cl_2] (15 mg, 0.02 mmol, 10 mol%) were added and the reaction mixture was stirred for 16 h at 70 °C, until complete conversion of **5** could be detected via TLC. After removing all volatiles in fine vacuum, the residue was taken up in dichloromethane (20 mL) and filtered over neutral aluminium oxide. Further purification could be achieved via preparative TLC (*n*-pentane/DCM 3:1). The red fraction at the solvent front was collected and the product precipitated using n-pentane at -18 °C. 15 mg (0.02 mmol, 10%) of the dark red solid **7** could be isolated after filtration and drying in vacuo. ^{1}H NMR (300.1 MHz, $CDCl_3$): δ_H = 0.45 (s, 36H, Si(C*H*$_3$)$_3$), 8.43 (s, 2H, *H*2, *H*9), 8.76 (d, $^{3}J_{H,H}$ = 9.3 Hz, 4H, *H*4, *H*7, *H*11, *H*14), 9.22 (d, $^{3}J_{H,H}$ = 9.5 Hz, 4H, *H*5, *H*6, *H*12, *H*13) ppm. ^{13}C NMR (75.5 MHz, $CDCl_3$): δ_C = 0.2, 101.3, 103.3, 117.9, 118.3, 122.1, 124.0, 124.3, 125.4, 125.8, 126.0, 131.9, 132.0, 132.2, 133.1, 134.4 ppm. HR-MS (EI+) m/z clcd. for $[C_{46}H_{46}Si_4]^+$: 710.26766 (found: 710.26695). IR (ATR), ṽ = 3357 (w), 3177 (w), 2922 (w), 2851 (m), 2776 (w), 2206 (w), 2119 (w), 1954 (w), 1895 (w), 1820 (w), 1633 (m), 1605 (m), 1465 (m), 1414 (w), 1061 (w), 1036 (w), 940 (w), 855 (s), 754 (s), 658 (w), 563 (w) cm^{-1}. λ_{max} (abs.) = 526 nm (DCM, ε = 4.85·10^{-4} L·mol^{-1}·cm^{-1}). λ_{max} (em.) = 531 nm (DCM), CV: $E_{1/2}(Ox_1)$ = 0.99 V, $E_{1/2}(Red_1)$ = -1.81 V (vs. Fc/Fc$^+$).

Synthesis of 1,3,8,10-tetrakis(3,5-bis(trifluoromethyl)phenyl)dibenzo-[*cd*,*lm*]perylene (8). 184 mg (184 mg, 0.2 mmol, 1.0 eq) of triflate **5**, 310 mg (3.2 mmol, 12.0 eq) potassium carbonate, 310 mg (1.2 mmol, 6.0 eq) (3,5-bis(trifluoromethyl)phenyl)boronic acid and 23 mg (0.02 mmol, 10 mol%) [$Pd(PPh_3)_4$] were stirred in 3 mL 1,4-dioxane and 1 mL water under argon atmosphere for 3 d at 110 °C. After complete conversion of **5** was detected via TLC, the reaction mixture was cooled down to room temperature and 10 mL water and 10 mL dichloromethane were added. The aqueous phase was separated and extracted with 10 mL dichloromethane. The combined organic layers were dried over $MgSO_4$. The crude product was purified by column chromatography (*n*-pentane/dichloromethane (1:1). 86 mg (0.07 mmol, 37%) of **8** were obtained as an orange solid. ^{1}H NMR (300.1 MHz, CD_2Cl_2): δ_H = 8.10 (s, 4 H, *p*-Ph*H*), 8.11 (s, 2H, *H*2, *H*9), 8.24 (s, 8 H, o-Ph*H*), 8.41 (d, $^3J_{H,H}$ = 9.5 Hz, 4H, *H*4, *H*7, *H*11, *H*14), 9.40 (d, $^3J_{H,H}$ = 9.7 Hz, 4H, *H*5, *H*6, *H*12, *H*13) ppm. ^{13}C NMR (125.8 MHz, $CDCl_3$): δ_C = 120.2, 121.9 (m), 122.4, 123.6, 124.5, 124.9, 125.0, 126.0, 126.2, 126.7, 129.2, 129.3, 130.9, 130.9, 132.3 (q, $^1J_{C,F}$ = 33.5 Hz), 134.7, 142.7 ppm. ^{19}F NMR (235.3 MHz, $CDCl_3$): δ_H = -62.6 (s) ppm. HR-MS (EI+) m/z clcd. for $[C_{58}H_{22}F_{24}]^+$: 1174.13383 (found: 1174.13295). IR (ATR), ṽ = 2961 (w), 1617 (w), 1464 (m), 1408 (m), 1335 (s), 1278 (m), 1182 (vs), 1078 (m), 1020 (w), 897 (w), 868 (w), 848 (m), 794 (w), 707 (w) cm^{-1}. λ_{max} (abs.) = 482 nm (DCM, ε = $1.11\cdot10^{-5}$ $L\cdot mol^{-1}\cdot cm^{-1}$). λ_{max} (em.) = 502 nm (DCM). CV: $E_{1/2}(Ox_2)$ = 1.53 V, $E_{1/2}(Ox_1)$ = 0.90 V, $E_{1/2}(Red_1)$ = -1.88 V (vs. Fc/Fc^+), $E_{1/2}(Red_2)$ = -2.17 V (vs. Fc/Fc^+).

Synthesis of 1,3,8,10-tetra(thiophen-2-yl)dibenzo[*cd*,*lm*]perylene (9). 184 mg (184 mg, 0.2 mmol, 1.0 eq) of triflate **5**, 310 mg (3.2 mmol, 12.0 eq) potassium carbonate, 154 mg (0.12 mmol, 6.0 eq) 2-thienylboronic acid and 23 mg (0.02 mmol, 10 mol%) [$Pd(PPh_3)_4$] were stirred in 3 mL 1,4-dioxane and 1 mL water under argon atmosphere for 3 d at 110 °C. After complete conversion of **5** was detected via TLC, the reaction mixture was cooled down to room temperature and 10 mL water and 10 mL dichloromethane were added. The aqueous phase was separated and extracted with 10 mL dichloromethane. The combined organic layers were dried over $MgSO_4$. The crude product was purified by column chromatography (*n*-pentane/dichloromethane (3:1). 28 mg (0.04 mmol, 22%) of **9** were obtained as a red solid. ^{1}H NMR (300.1 MHz, CD_2Cl_2): δ_H = 7.35 (dd, $^3J_{H,H}$ = 5.1 Hz, $^4J_{H,H}$ = 3.6 Hz, 4H), 7.53 (dd, $^3J_{H,H}$ = 3.5 Hz, $^4J_{H,H}$ = 1.1 Hz, 4H), 7.62 (d, $^3J_{H,H}$ = 4.1 Hz, 4H) 8.36 (s, 2H, *H*2, *H*9), 8.85 (d, $^3J_{H,H}$ = 9.5 Hz, 4H, *H*4, *H*7, *H*11, *H*14), 9.32 (d, $^3J_{H,H}$ = 9.7 Hz, 4H, *H*5, *H*6, *H*12, *H*13) ppm. ^{13}C NMR (75.5 MHz, $CDCl_3$): δ_C = 123.7, 125.6, 125.8, 126.2, 126.8, 127.8, 128.6, 128.7, 129.3, 129.8,131.2, 132.4, 132.4, 142.2 ppm. HR-MS (EI+) m/z clcd. for $[C_{42}H_{22}S_{24}]^+$: 654.06043 (found: 654.06152). IR (ATR), ṽ = 2958 (m), 2923 (m), 2853 (w), 2188 (w), 2160 (m), 2031 (m), 2012 (w), 1908 (w), 1259 (w), 1090 (m), 1018 (m), 793 (s), 751 (m), 687 (m) cm^{-1}. λ_{max} (abs.) = 505 nm (DCM, ε = $7.12\cdot10^{-4}$ $L\cdot mol^{-1}\cdot cm^{-1}$). λ_{max} (em.) = 537 nm (DCM). CV: $E_{1/2}(Ox_2)$ = 0.94 V, $E_{1/2}(Ox_1)$ = 0.41 V, $E_{1/2}(Red_1)$ = -1.94 V (vs. Fc/Fc^+), $E_{1/2}(Red_2)$ = -2.20 V (vs. Fc/Fc^+).

Synthesis of 1,3,8,10-tetraphenyldibenzo[cd,lm]perylene (10). To a solution of 450 mg (3.3 mmol, 16.5 eq) dried zinc chloride dissolved in 4 mL THF were added dropwise 2.2 mL (3.2 mmol, 16 eq) phenyl lithium (1.43 M in dibutyl ether) at 0 °C. After stirring for 1 h at room temperature, the solution of the zinc organyl was added to a solution of 146 mg (0.2 mmol, 1.0 eq) pivalate **4**, 12 mg (0.04 mmol, 20 mol%) [$Ni(COD)_2$] and 22 mg (0.08 mmol, 40 mol%) PCy_3 in 2 mL DMF. The reaction mixture was then stirred for 16 h under exclusion of light at 70 °C. After complete conversion of **4** was detected via TLC, the reaction mixture was cooled down to room temperature and 10 mL water and 10 mL dichloromethane were added. The aqueous phase was separated and extracted with 10 mL dichloromethane. The combined organic layers were dried over $MgSO_4$. The crude product was purified by column chromatography (*n*-pentane/dichloromethane (3:1). The solvent was evaporated and the product washed with *n*-pentane. 45 mg (0.07 mmol, 36%) of **10** were obtained as an orange solid. X-ray suited single crystals could be obtained by layering a saturated solution of **10** in dichloromethane with *n*-pentane at -18 °C. ^{1}H NMR (300.1 MHz, CD_2Cl_2): δ_H = 7.53-7.57 (m, 4H, *p*-Ph*H*), 7.59-7.66 (m, 8H, *m*-Ph*H*), 7.74-7.79 (m, 8H, *o*-Ph*H*), 8.14 (s, 2H, *H*2, *H*9), 8.54 (d, $^3J_{H,H}$ = 9.5 Hz, 4H, *H*4, *H*7, *H*11, *H*14), 9.23 (d, $^3J_{H,H}$ = 9.7 Hz, 4H, *H*5, *H*6, *H*12, *H*13) ppm. ^{13}C NMR (75.5 MHz, $CDCl_3$): δ_C = 123.1, 123.1, 125.5, 125.8, 126.1, 127.8, 128.3, 128.6, 129.7, 130.9, 137.7, 141.2 ppm. HR-MS (EI+) m/z clcd. for $[C_{50}H_{30}]^+$: 630.23475 (found: 630.23280). IR (ATR), ṽ = 3057 (m), 3027 (m), 2926 (w), 2247 (w), 1956 (w), 1897 (w), 1598 (m), 1484 (m), 1439 (w), 1351 (w), 1255 (w), 1185 (w), 954 (w), 904 (s), 835 (w), 790 (vs), 698 (vs), 648 (w), 540 (w) cm^{-1}. λ_{max} (abs.) = 483 nm (DCM, ε = $6.17\cdot10^{-4}$ $L\cdot mol^{-1}\cdot cm^{-1}$). λ_{max} (em.) = 500 nm (DCM). CV: $E_{1/2}(Ox_2)$ = 1.00 V, $E_{1/2}(Ox_1)$ = 0.34 V, $E_{1/2}(Red_1)$ = -2.18 V (vs. Fc/Fc^+).

Synthesis of 1,3,8,10-tetrakis(4-(tert-butyl)phenyl)dibenzo[cd,lm]-perylene (11). 146 mg (0.2 mmol, 1.0 eq) of pivalate **4**, 570 mg (3.2 mmol, 16.0 eq) (4-(tert-butyl)phenyl)boronic acid, 679 mg (3.2 mmol, 16.0 eq) dried potassium phosphate and 28 mg (0.04 mmol, 20 mol%) [$NiCl_2(PCy_3)_2$] were dissolved in 3 mL 1,4-dioxane and stirred for 18 h at 110 °C under argon atmosphere. After removal of all volatile components in vacuo, the residue was taken up in 20 mL dichloromethane and filtered over neutral aluminium oxide. The crude product was crystalized from dichloromethane/n-pentane (1:1) at -18 °C. 75 mg (0.08 mmol, 44%) of **11** could be isolated as brownish orange needles. ^{1}H NMR (300.1 MHz, CD_2Cl_2): δ_H = 1.33 (s, 36H, C(C*H*$_3$)$_3$), 7.63 (d, 8H, $^3J_{H,H}$ = 8.3 Hz, *o*-Ph*H*), 7.69 (d, 8H, $^3J_{H,H}$ = 8.3 Hz, *m*-Ph*H*), 8.12 (s, 2H, *H*2, *H*9), 8.57 (d, $^3J_{H,H}$ = 9.4 Hz, 4H, *H*4, *H*7, *H*11, *H*14), 9.22 (d, $^3J_{H,H}$ = 9.7 Hz, 4H, *H*5, *H*6, *H*12, *H*13) ppm. ^{13}C NMR (125.8 MHz, $CDCl_3$): δ_C = 31.6, 34.9, 122.8, 123.9, 125.5, 125.9, 126.2, 128.2, 129.9, 130.6, 137.6, 138.3, 150.4 ppm. HR-MS (EI+) m/z clcd. for $[C_{66}H_{62}]^+$: 854.48515 (found: 854.48231). IR (ATR), ṽ = 2958 (s), 2904 (w), 2865 (w), 1928 (w), 1881 (w), 1602 (w), 1497 (m), 1462 (m), 1394 (w), 1270 (w), 1113 (m), 1050 (w), 1014 (w), 903 (s), 837 (m), 791 (w), 725 (w), 597 (w) cm^{-1}. λ_{max} (abs.) = 485 nm (DCM, ε = $9.19\cdot10^{-4}$ $L\cdot mol^{-1}\cdot cm^{-1}$). λ_{max} (em.) = 506 nm (DCM). CV: $E_{1/2}(Ox_2)$ = 0.73 V, $E_{1/2}(Ox_1)$ = 0.29 V, $E_{1/2}(Red_1)$ = -2.12 V (vs. Fc/Fc^+).

Synthesis of 3,10-dihydroxy-2,9-diphenyldibenzo[*cd*,*lm*]perylene-1,8-dione (12). 4.0 g (14.7 mmol, 1.0 eq) 3-hydroxy-2-phenyl-1*H*-phenalen-1-one and 20 g potassium hydroxide were heated in a Ni crucible for 3 h at 285 °C on air. During the reaction, the melt turned black. After cooling to room temperature, the solid was taken up in 1 l water, filtered and precipitated with 200 mL of 2 M acetic acid. The purple precipitate was filtered and washed with water and hot methanol. The solid was dried

at 120 °C overnight. 880 mg (3.3 mmol, 22%) of the purple solid **12** were obtained and directly used for further stages, since a characterization was not possible due to the insolubility in organic solvents. HR-MS (APCI+) m/z clcd. for $[C_{38}H_{19}O_4]^+$: 539.1289 (found: 539.1309). IR (ATR), $\tilde{\nu}$ = 3466 (w), 3050 (w), 1608 (m), 1549 (vs), 1404 (m), 1374 (m), 1232 (vs), 1178 (s), 1093 (s), 951 (w), 856 (m), 810 (w), 776 (m), 738 (m), 636 (w), 499 (w) cm^{-1}.

Synthesis of ((2,9-diphenyldibenzo[cd,lm]perylene-1,3,8,10-tetrayl)-tetrakis(oxy))tetrakis(trimethylsilane) (13). Peropyrenequinone **12** (590 mg, 1.06 mmol, 1.0 eq), Zn dust (556 g, 8.5 mmol, 8.0 eq) and trimethylsilyl chloride (1.1 mL, 8.5 mmol, 8.0 eq) were stirred for 3 h under argon atmosphere at 100 °C in 40 mL 1,4-dioxane. The greenish solution was cooled to room temperature and all volatile components were removed in vacuo. The residue was taken up in 40 mL dichloromethane and filtered. After removing the solvent under vacuum, the crude product was washed with *n*-pentane. 360 mg (0.43 mmol, 41%) of **13** were obtained as an orange solid. ^1H NMR (300.1 MHz, $CDCl_3$): δ_H = -0.04 (s, 36H, $Si(CH_3)_3$), 7.42 (t, $^3J_{H,H}$ = 6.8 Hz, 2H, *p*-Ph*H*), 7.52 (t, $^3J_{H,H}$ = 7.4 Hz, 4H, *m*-Ph*H*), 7.67 (d, $^3J_{H,H}$ = 7.1 Hz, 4H, *o*-Ph*H*), 8.49 (d, $^3J_{H,H}$ = 9.4 Hz, 4H, *H*4, *H*7, *H*11, *H*14), 9.07 (d, $^3J_{H,H}$ = 9.6 Hz, 4H, *H*5, *H*6, *H*12, *H*13) ppm. ^{13}C NMR (75.5 MHz, $CDCl_3$): δ_C =0.5, 119.7, 120.6, 122.3, 125.5, 126.7, 127.5, 128.2, 132.9, 136.4, 148.8 ppm. HR-MS (FD+) m/z clcd. for $[C_{50}H_{54}O_4Si_4]^+$: 830.30991 (found: 830.31051). IR (ATR), $\tilde{\nu}$ = 2952 (w), 2922 (w), 1583 (w), 1471 (w), 1398 (m), 1355 (w), 1308 (m), 1250 (s), 1144 (m), 1092 (m), 1029 (m), 845 (vs), 789 (m), 723 (m), 617 (w), 582 (w) cm^{-1}.

Synthesis of 2,9-diphenyldibenzo[cd,lm]perylene-1,3,8,10-tetrayl tetrakis(2,2-dimethylpropanoate) (14). Peropyrenequinone **12** (900 mg, 1.67 mmol, 1.0 eq), Zn dust (872 mg, 13.3 mmol, 8.0 eq) and pivalic anhydride (2.7 mL, 13.3 mmol, 8.0 eq) were refluxed for 3 d under argon atmosphere in 200 mL 1,4-dioxane. The brownish green solution was cooled to room temperature, filtered over a pad of neutral aluminium oxide and washed with 100 mL dichloromethane subsequently. After removing the solvent in fine vacuum, the crude product was suspended in 50 mL *n*-pentane and filtered. 250 mg (0.34 mmol, 22%) of the brown solid **14** were obtained after drying in vacuo. ^1H NMR (300.1 MHz, CD_2Cl_2): δ_H = 1.19 (s, 36H, $C(CH_3)_3$), 7.32-7.48 (m, 10H, Ph*H*), 8.27 (d, $^3J_{H,H}$ = 9.4 Hz, 4H, *H*4, *H*7, *H*11, *H*14), 9.23 (d, $^3J_{H,H}$ = 9.5 Hz, 4H, *H*5, *H*6, *H*12, *H*13) ppm. ^{13}C NMR: not recorded due to insolubility. HR-MS (APCI+) m/z clcd. for $[C_{58}H_{55}O_4]^+$: 879.3891 (found: 879.3903). IR (ATR), $\tilde{\nu}$ = 2959 (w), 2925 (w), 2854 (w), 1751 (vs), 1633 (m),1598 (m), 1477 (w), 1395 (w), 1367 (w), 1263 (m), 1217 (m), 1100 (s), 1027 (m), 789 (m), 756 (m), 731 (m), 699 (w) cm^{-1}.

Concentration-dependent UV-Vis spectroscopy

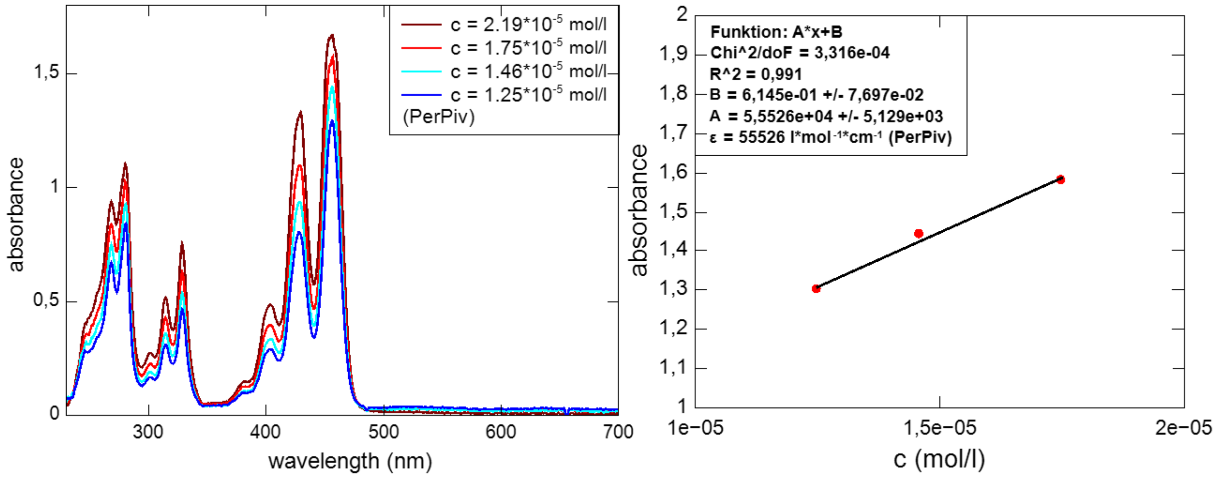

Figure S1. Left: UV-Vis spectra of **4** at four different concentration, recorded in CH_2Cl_2. Center: plot of the absorbance at λ_{max} versus the corresponding concentration to determine the molecular decadic attenuation coefficient ε from the slope (optical pass length = 1 cm).

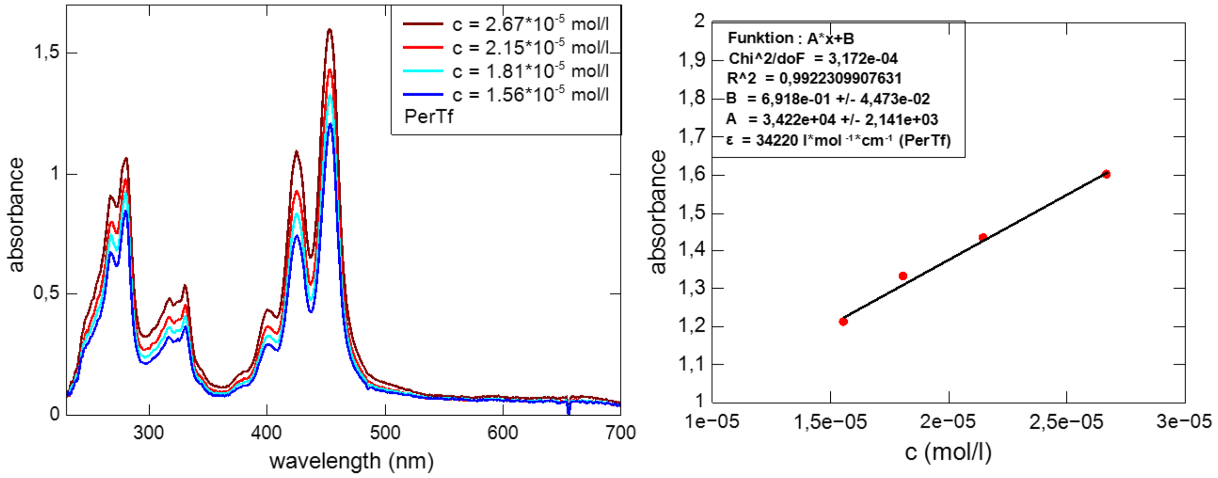

Figure S2. Left: UV-Vis spectra of **5** at four different concentration, recorded in CH_2Cl_2. Center: plot of the absorbance at λ_{max} versus the corresponding concentration to determine the molecular decadic attenuation coefficient ε from the slope (optical pass length = 1 cm).

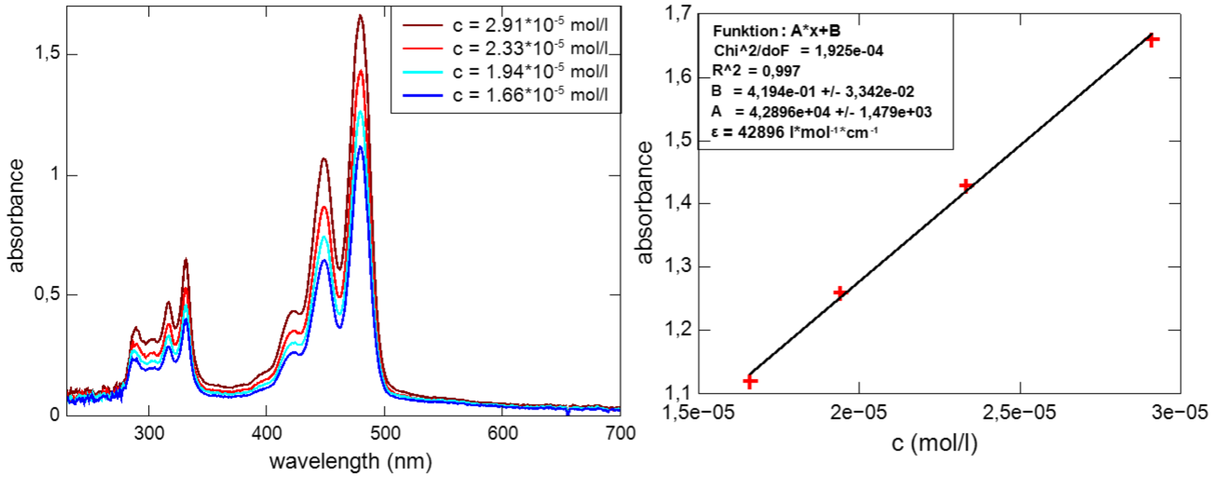

Figure S3. Left: UV-Vis spectra of **2** at four different concentration, recorded in CH_2Cl_2. Center: plot of the absorbance at λ_{max} versus the corresponding concentration to determine the molecular decadic attenuation coefficient ε from the slope (optical pass length = 1 cm).

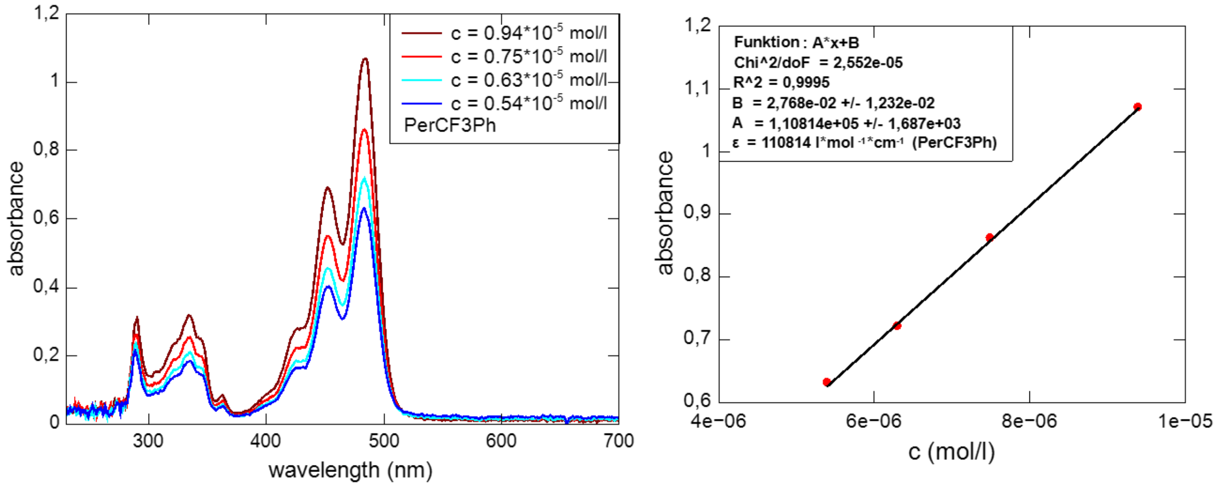

Figure S4. Left: UV-Vis spectra of **8** at four different concentration, recorded in CH_2Cl_2. Center: plot of the absorbance at λ_{max} versus the corresponding concentration to determine the molecular decadic attenuation coefficient ε from the slope (optical pass length = 1 cm).

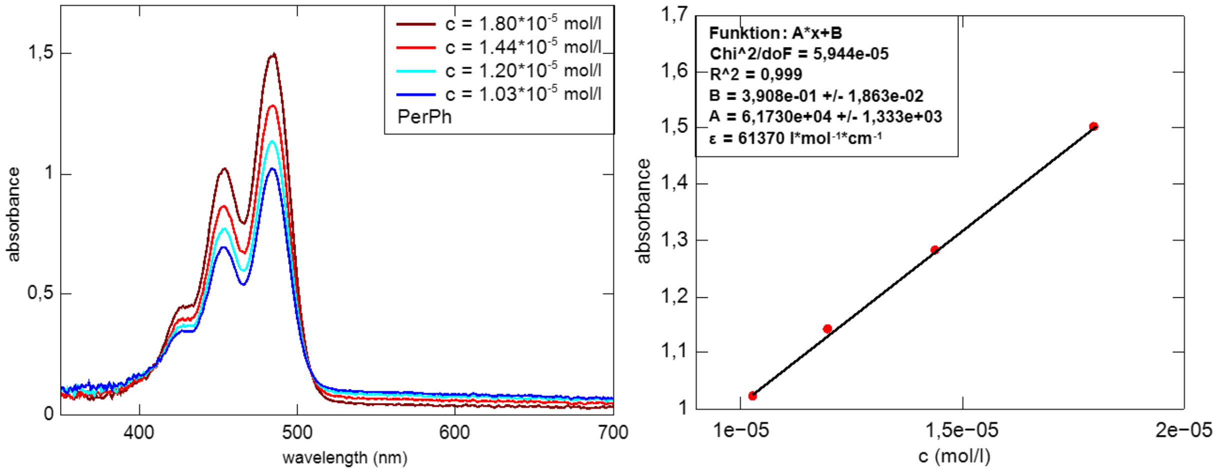

Figure S5. Left: UV-Vis spectra of **10** at four different concentration, recorded in CH_2Cl_2. Center: plot of the absorbance at λ_{max} versus the corresponding concentration to determine the molecular decadic attenuation coefficient ε from the slope (optical pass length = 1 cm).

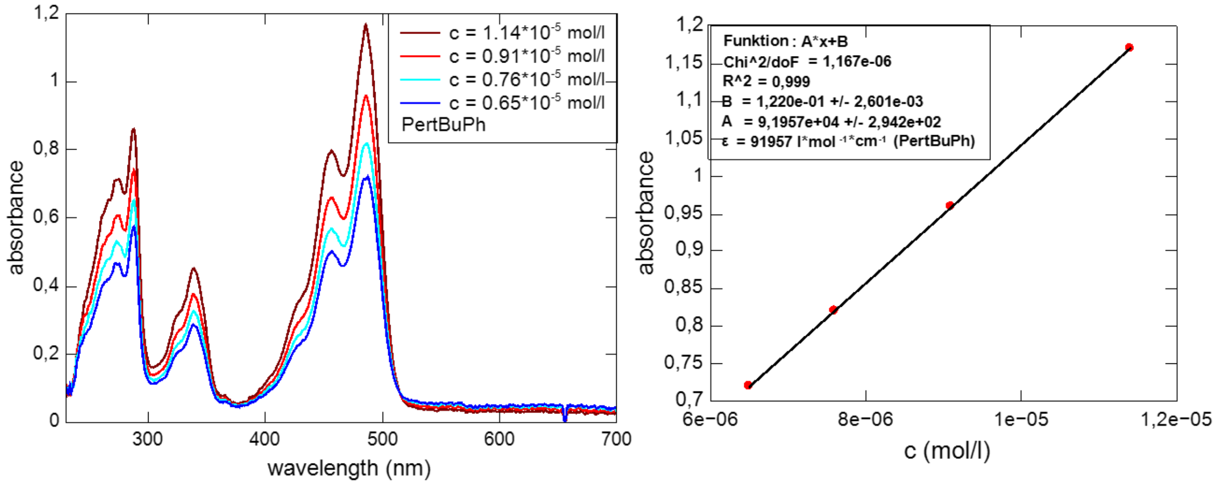

Figure S6. Left: UV-Vis spectra of **11** at four different concentration, recorded in CH_2Cl_2. Center: plot of the absorbance at λ_{max} versus the corresponding concentration to determine the molecular decadic attenuation coefficient ε from the slope (optical pass length = 1 cm).

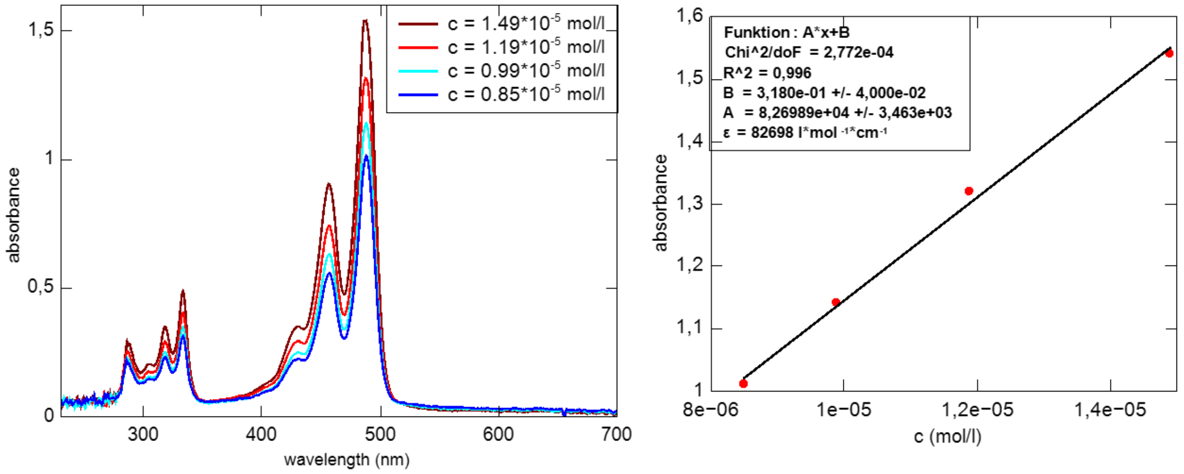

Figure S7. Left: UV-Vis spectra of **3** at four different concentration, recorded in CH_2Cl_2. Center: plot of the absorbance at λ_{max} versus the corresponding concentration to determine the molecular decadic attenuation coefficient ε from the slope (optical pass length = 1 cm).

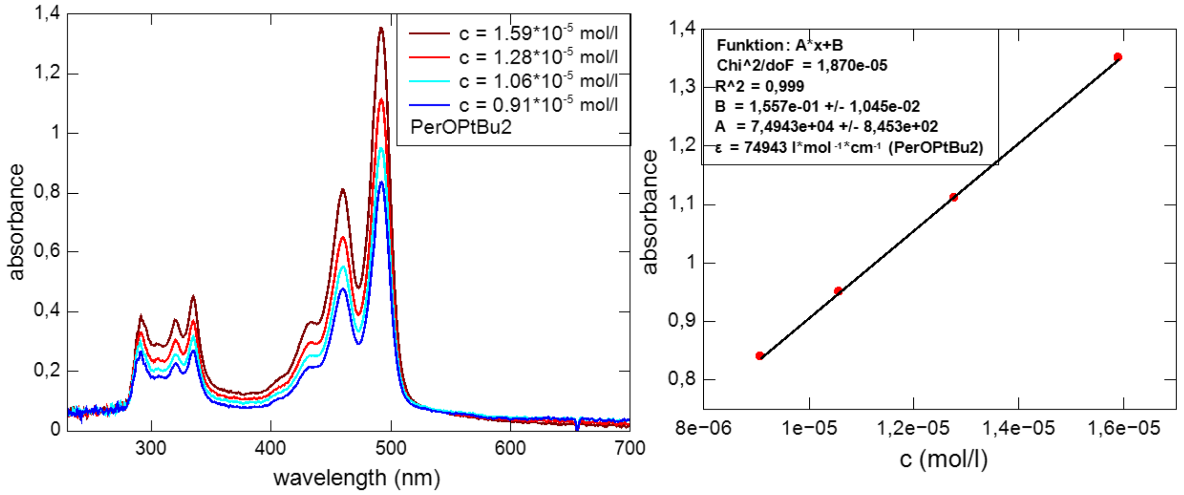

Figure S8. Left: UV-Vis spectra of **6** at four different concentration, recorded in CH_2Cl_2. Center: plot of the absorbance at λ_{max} versus the corresponding concentration to determine the molecular decadic attenuation coefficient ε from the slope (optical pass length = 1 cm).

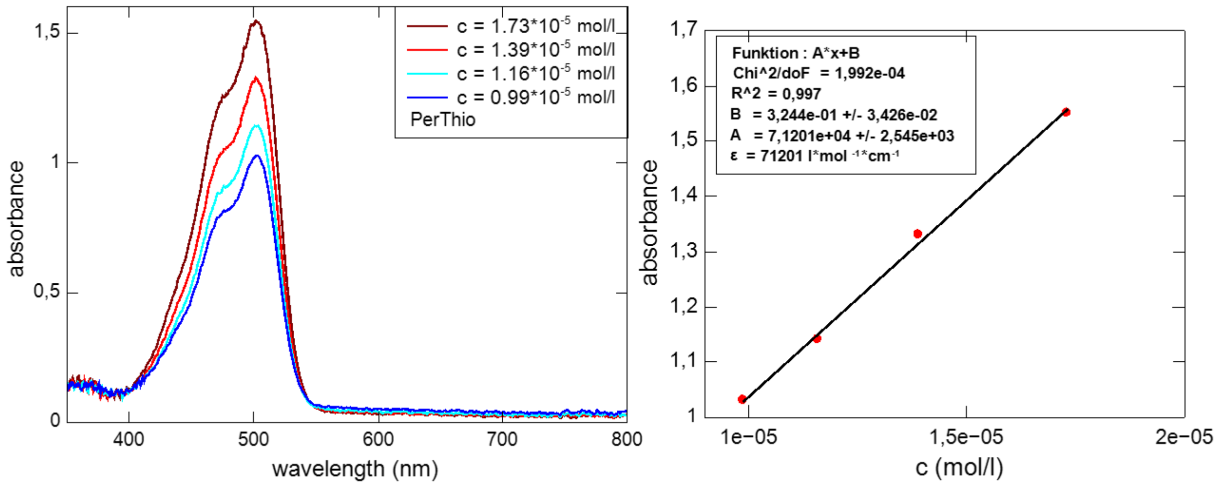

Figure S9. Left: UV-Vis spectra of **9** at four different concentration, recorded in CH_2Cl_2. Center: plot of the absorbance at λ_{max} versus the corresponding concentration to determine the molecular decadic attenuation coefficient ε from the slope (optical pass length = 1 cm).

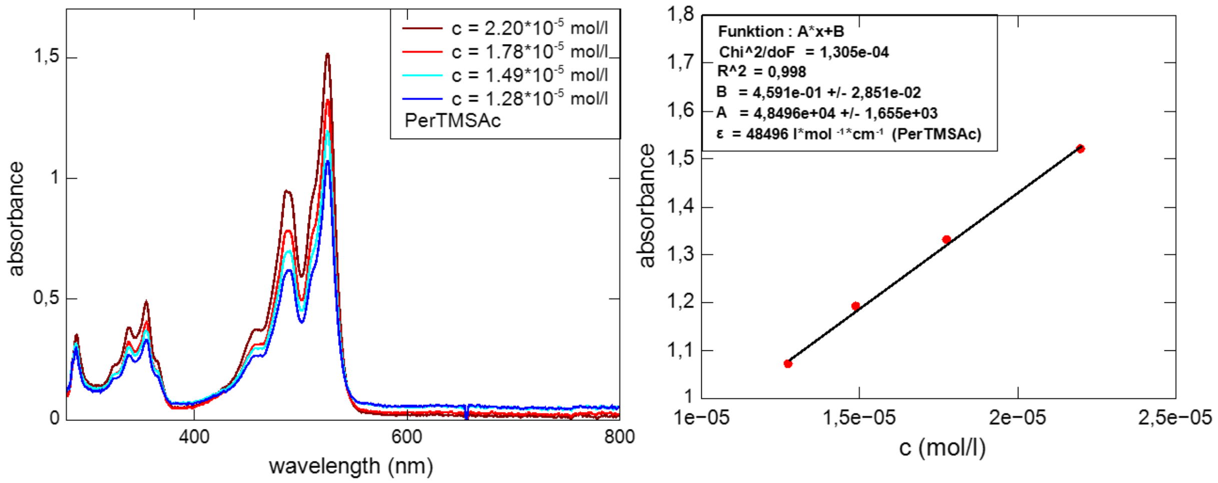

Figure S10. Left: UV-Vis spectra of **7** at four different concentration, recorded in CH_2Cl_2. Center: plot of the absorbance at λ_{max} versus the corresponding concentration to determine the molecular decadic attenuation coefficient ε from the slope (optical pass length = 1 cm).

Determination of fluorescence quantum yields

Fluorescence quantum yields (Φ_{PL}) were recorded by dilution method using a fluorescein solution (0.1 M in aqueous NaOH, Φ_{flu} = 0.95) [27] as reference. Both, the samples and the reference were measured at low concentrations in order to ensure a linear relationship between the intensity of emitted light and the concentration of the absorbing/emitting species.

The quantum yields of the samples (Φ_s) were determined by the following equation 1.[6]

$$\Phi_{flu}=\frac{grad(s)}{grad(flu)}*\frac{n^2(s)}{n^2(flu)}*\Phi_{flu}$$

In equation 1, *grad* is the slope of the "emission versus absorbance" plot of the samples (s) and the standard fluorescein (flu). n is the refractive index of the used solvents.

In the following figures S11 and S12, the "emission versus absorbance" plots and the linear regression graphs for the determination of the fluorescence quantum yields of **3**-**11** are shown. The plot of the most efficient fluorescence dye **3** was calibrated against fluorescein in order to calculate the fluorescence quantum yields. The fluorescence quantum yield of trimethylsilyl ether **2** has been determined previously.[28] Note that in the case of phenyl-substituted congeners **13** and **14**, the emission values at suitable low concentrations were too low to determine fluorescence quantum yields.

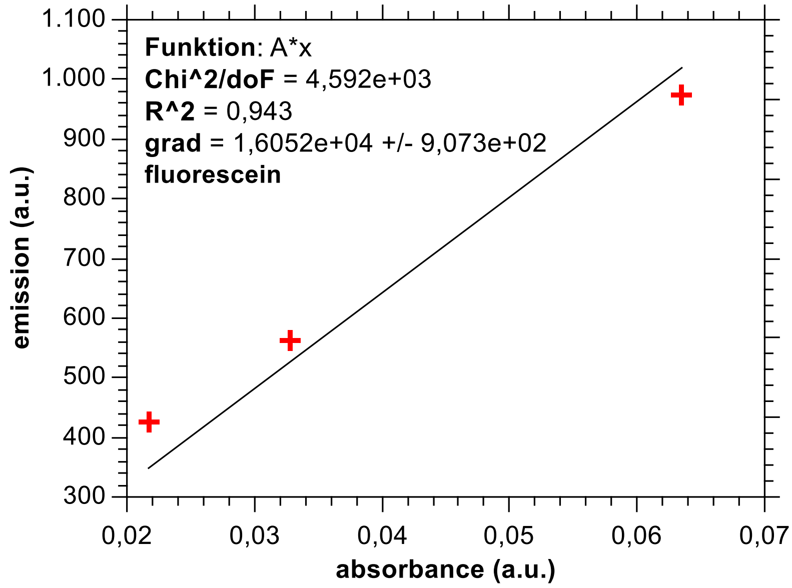

Figure S11. "emission versus absorption" plots fluorescein as 0.1 M solution in aqueous NaOH for direct referencing.

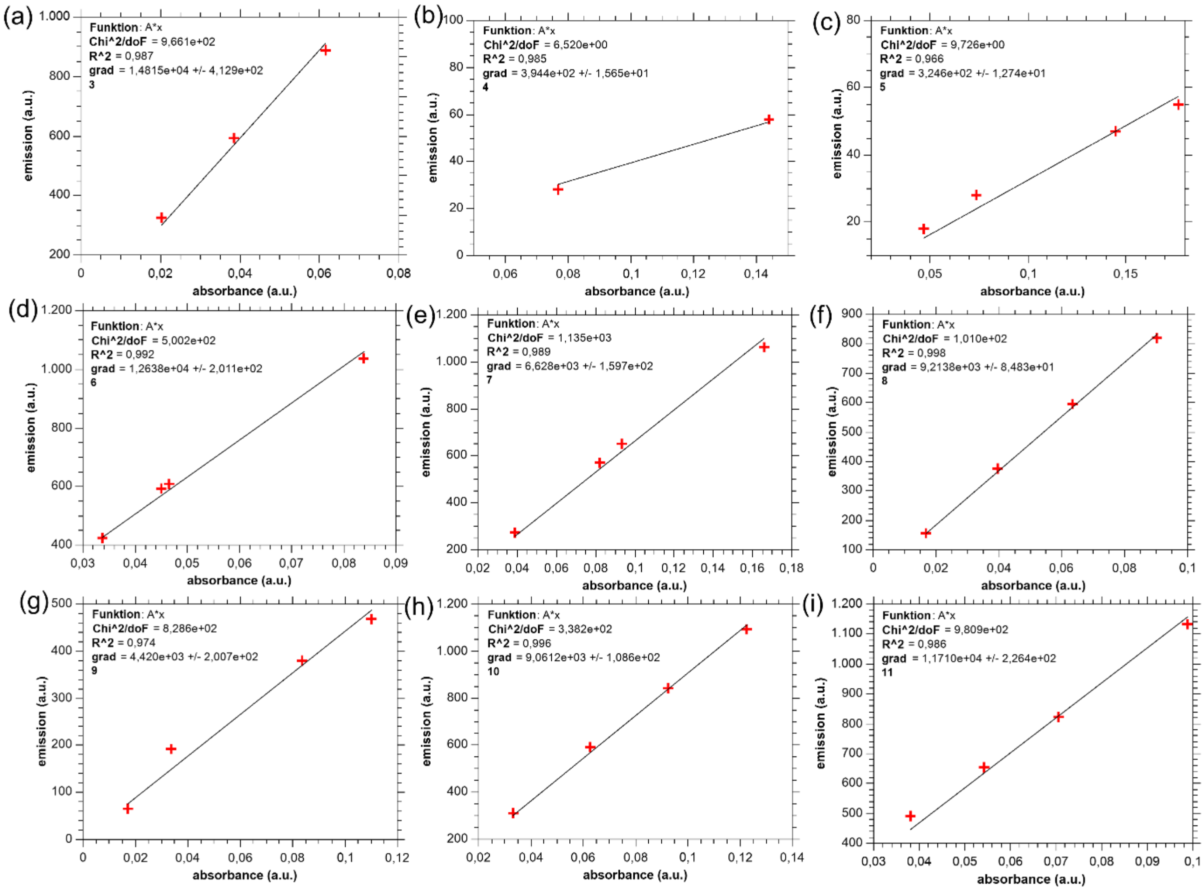

Figure S12. "emission versus absorption" plots of (a) **3**; (b) **4**; (c) **5**; (d) **6**; (e) **7**; (f) **8**; (g) **9**; (h) **10**; (i) **11** recorded in dichloromethane. The concentrations were in the low 10^{-6} M range.

Additional CV spectra

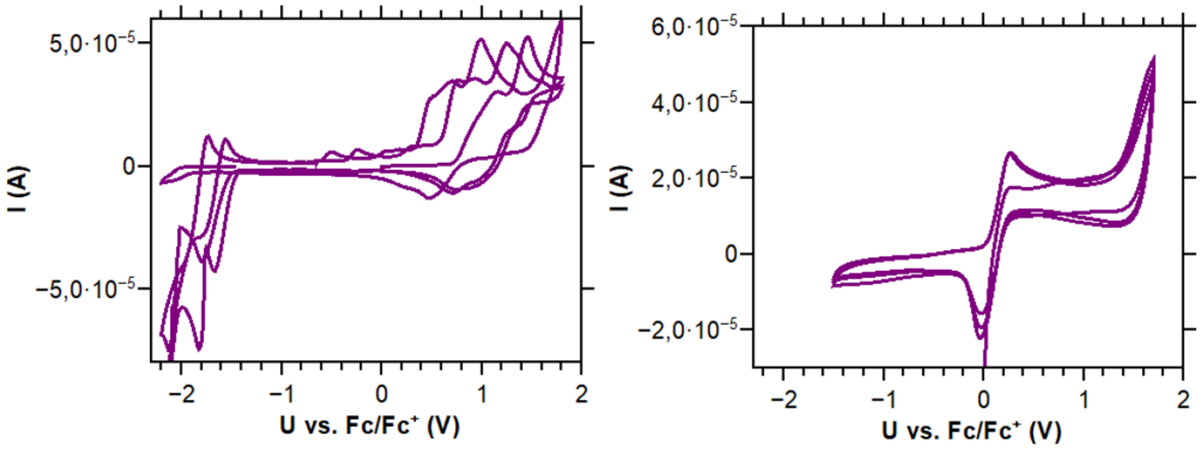

Figure S13. Left: Raw data of the cyclovoltammography of **4** (three scans were performed, only the first scan was usable for analysis, since **4** decomposes during CV measurement). Right: Cyclovoltammogramm of **4** after addition of 1 mM ferrocene (measured in CH_2Cl_2, 0.1 M *n*-Bu_4NPF_6, 100 mV s^{-1} scan rate, glassy carbon working electrode, platinum reference electrode).

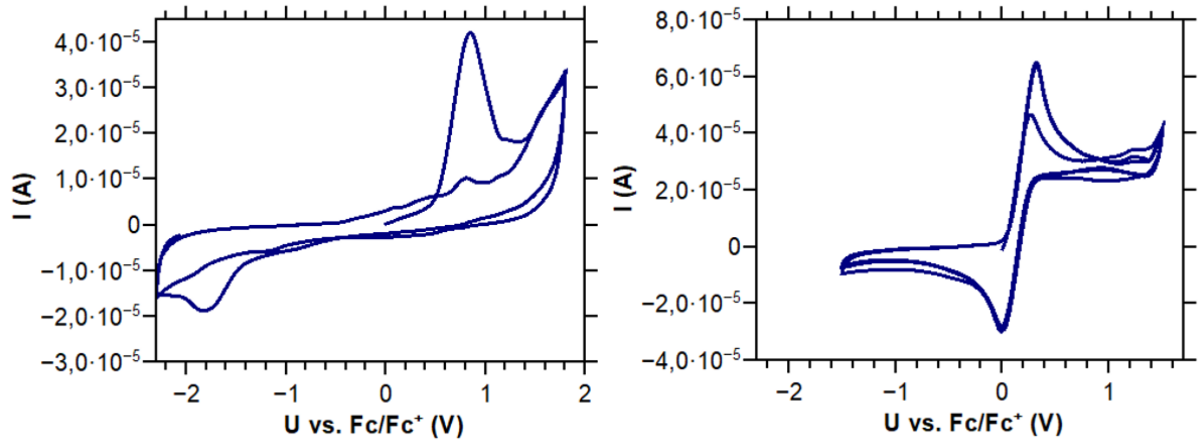

Figure S14. Left: Raw data of the cyclovoltammography of **5** (three scans were performed, only the first scan was usable for analysis, since **5** decomposes during CV measurement). Right: Cyclovoltammogramm of **5** after addition of 1 mM ferrocene (measured in CH_2Cl_2, 0.1 M *n*-Bu_4NPF_6, 100 mV s^{-1} scan rate, glassy carbon working electrode, platinum reference electrode).

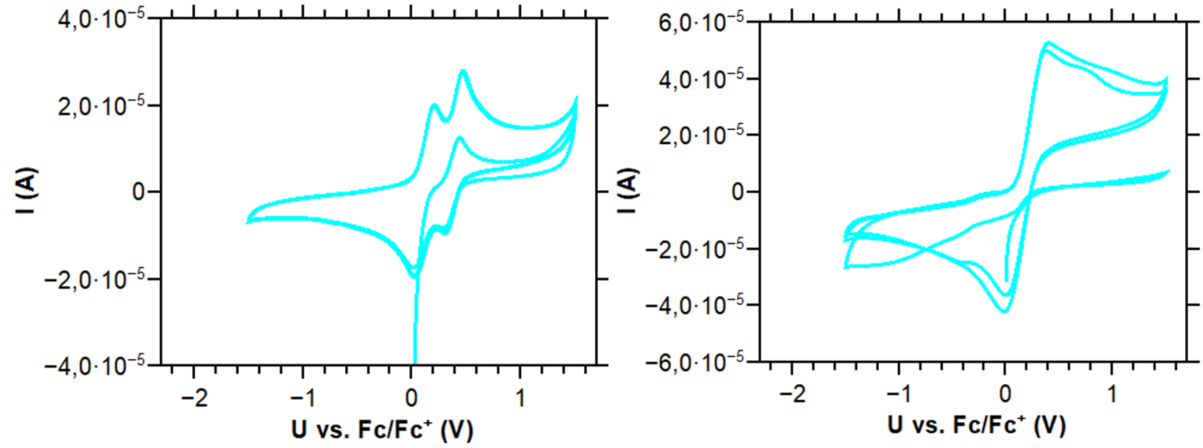

Figure S15. Left: Raw data of the cyclovoltammography of **2** (three scans were performed, the third one was used for analysis). Right: Cyclovoltammogramm of **2** after addition of 1 mM ferrocene (measured in CH_2Cl_2, 0.1 M *n*-Bu_4NPF_6, 100 mV s^{-1} scan rate, glassy carbon working electrode, platinum reference electrode).

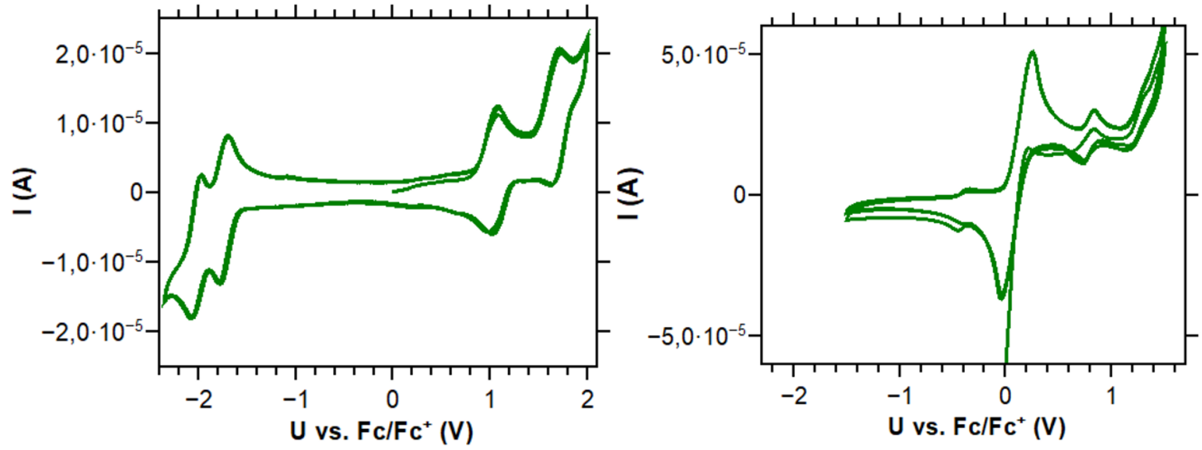

Figure S16. Left: Raw data of the cyclovoltammography of **8** (three scans were performed, the third one was used for analysis). Right: Cyclovoltammogramm of **8** after addition of 1 mM ferrocene (measured in CH_2Cl_2, 0.1 M *n*-Bu_4NPF_6, 100 mV s^{-1} scan rate, glassy carbon working electrode, platinum reference electrode).

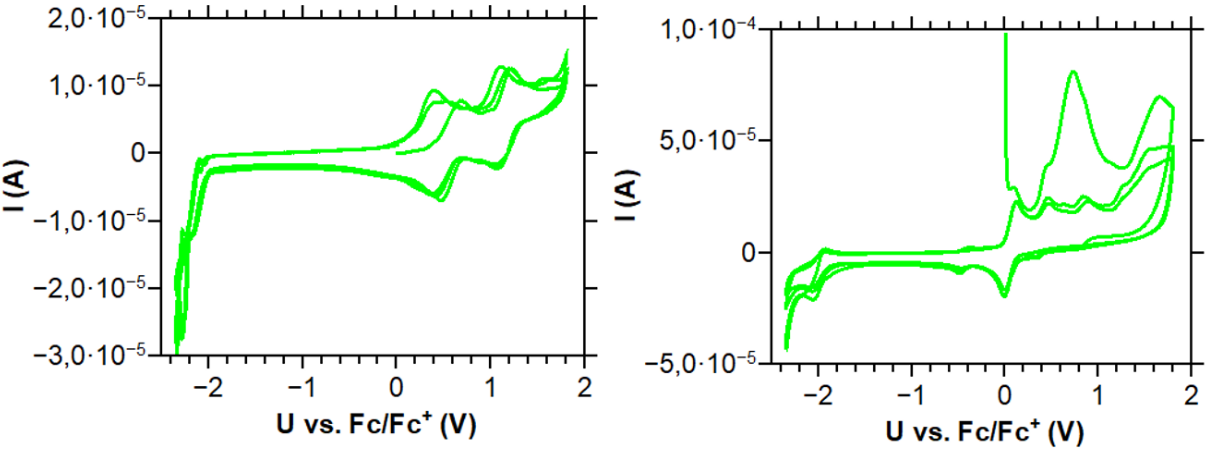

Figure S17. Left: Raw data of the cyclovoltammography of **10** (three scans were performed, the third one was used for analysis). Right: Cyclovoltammogramm of **10** after addition of 1 mM ferrocene (measured in CH_2Cl_2, 0.1 M *n*-Bu_4NPF_6, 100 mV s^{-1} scan rate, glassy carbon working electrode, platinum reference electrode).

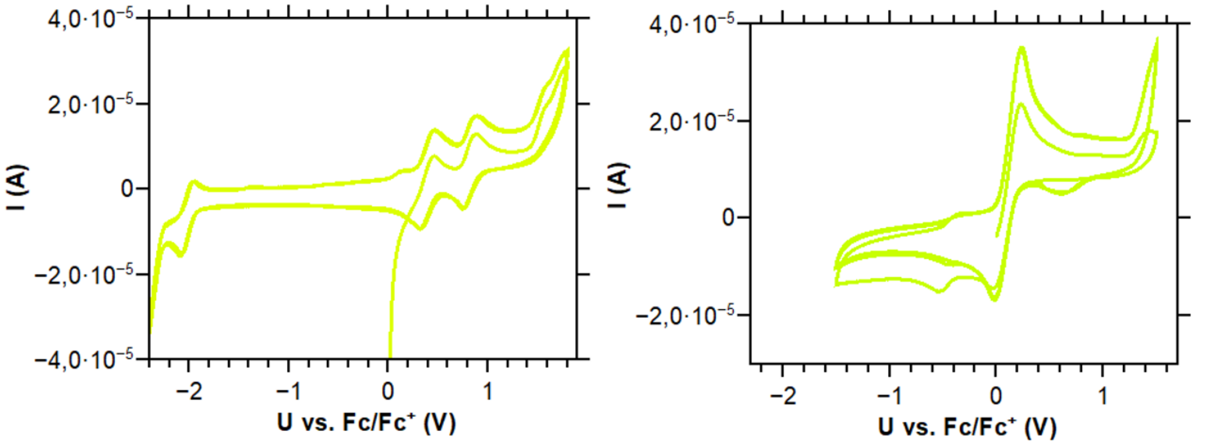

Figure S18. Left: Raw data of the cyclovoltammography of **11** (three scans were performed, the third one was used for analysis). Right: Cyclovoltammogramm of **11** after addition of 1 mM ferrocene (measured in CH_2Cl_2, 0.1 M *n*-Bu_4NPF_6, 100 mV s^{-1} scan rate, glassy carbon working electrode, platinum reference electrode).

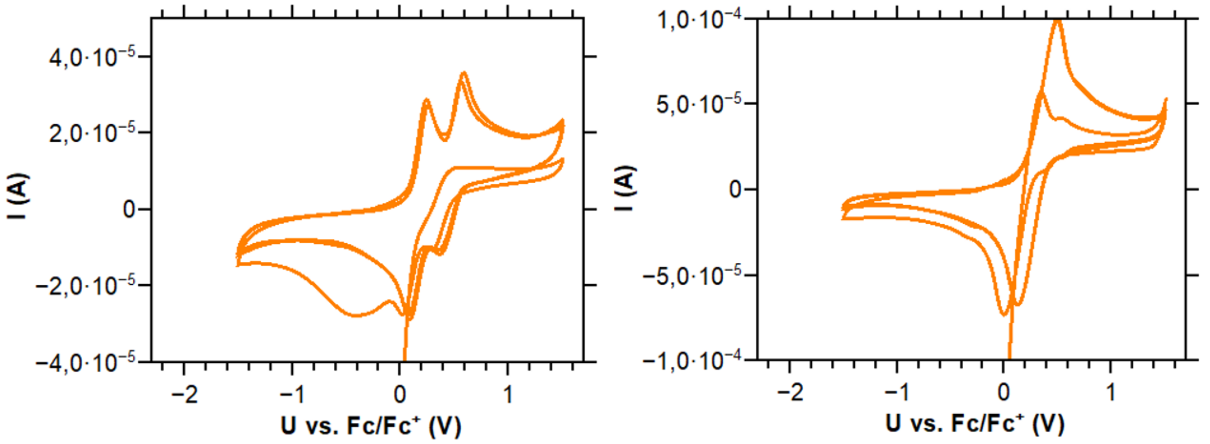

Figure S19. Left: Raw data of the cyclovoltammography of **3** (three scans were performed, the third one was used for analysis). Right: Cyclovoltammogramm of **3** after addition of 1 mM ferrocene (measured in CH_2Cl_2, 0.1 M *n*-Bu_4NPF_6, 100 mV s^{-1} scan rate, glassy carbon working electrode, platinum reference electrode).

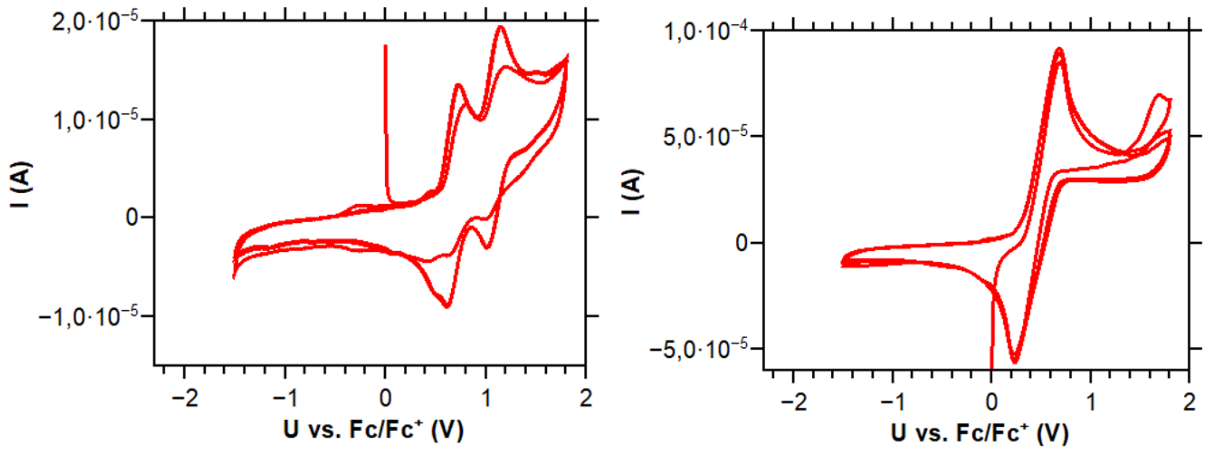

Figure S20. Left: Raw data of the cyclovoltammography of **6** (three scans were performed, the third one was used for analysis). Right: Cyclovoltammogramm of **6** after addition of 1 mM ferrocene (measured in CH_2Cl_2, 0.1 M *n*-Bu_4NPF_6, 100 mV s^{-1} scan rate, glassy carbon working electrode, platinum reference electrode).

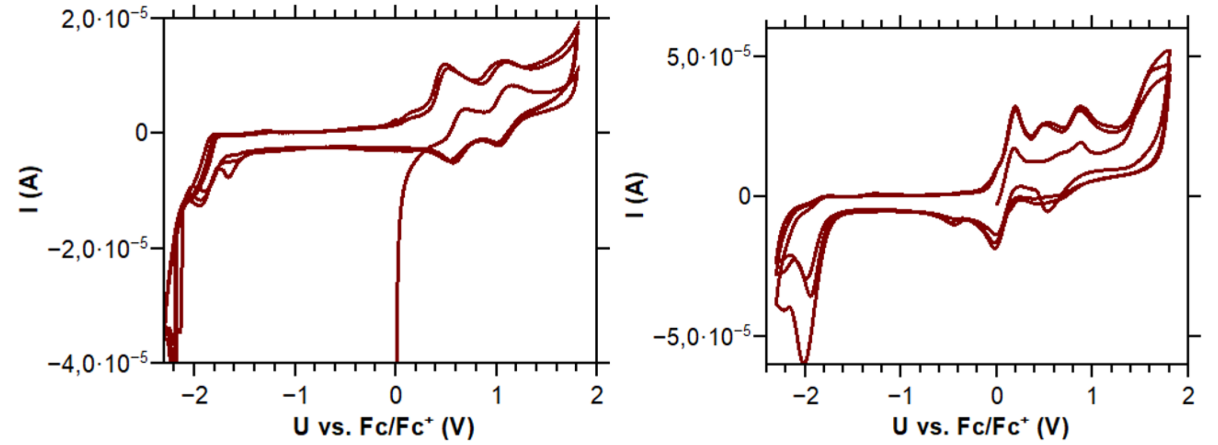

Figure S21. Left: Raw data of the cyclovoltammography of **9** (three scans were performed, the third one was used for analysis). Right: Cyclovoltammogramm of **9** after addition of 1 mM ferrocene (measured in CH_2Cl_2, 0.1 M *n*-Bu_4NPF_6, 100 mV s^{-1} scan rate, glassy carbon working electrode, platinum reference electrode).

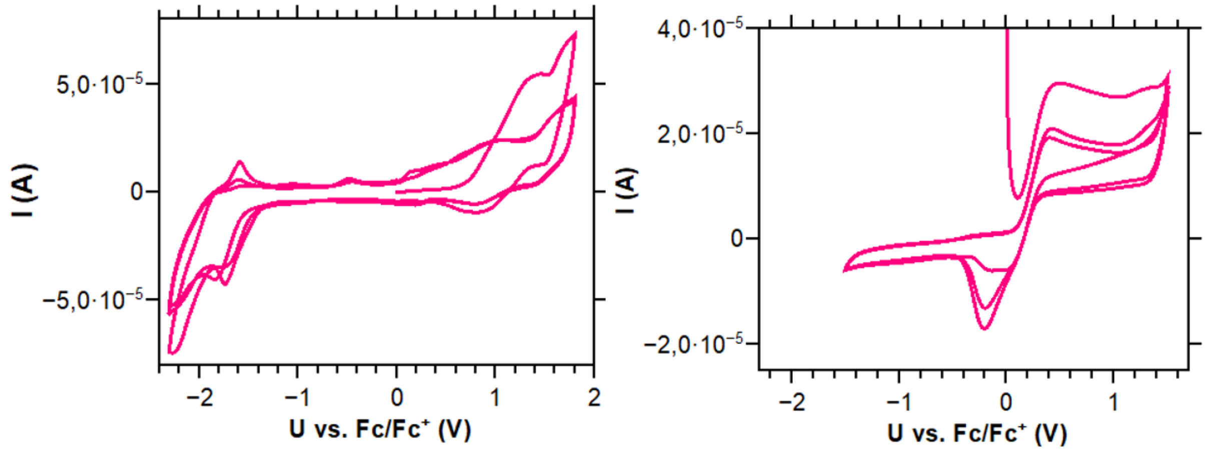

Figure S22. Left: Raw data of the cyclovoltammography of **7** (three scans were performed, the third one was used for analysis). Right: Cyclovoltammogramm of **7** after addition of 1 mM ferrocene (measured in CH_2Cl_2, 0.1 M *n*-Bu_4NPF_6, 100 mV s^{-1} scan rate, glassy carbon working electrode, platinum reference electrode).

TD-DFT results

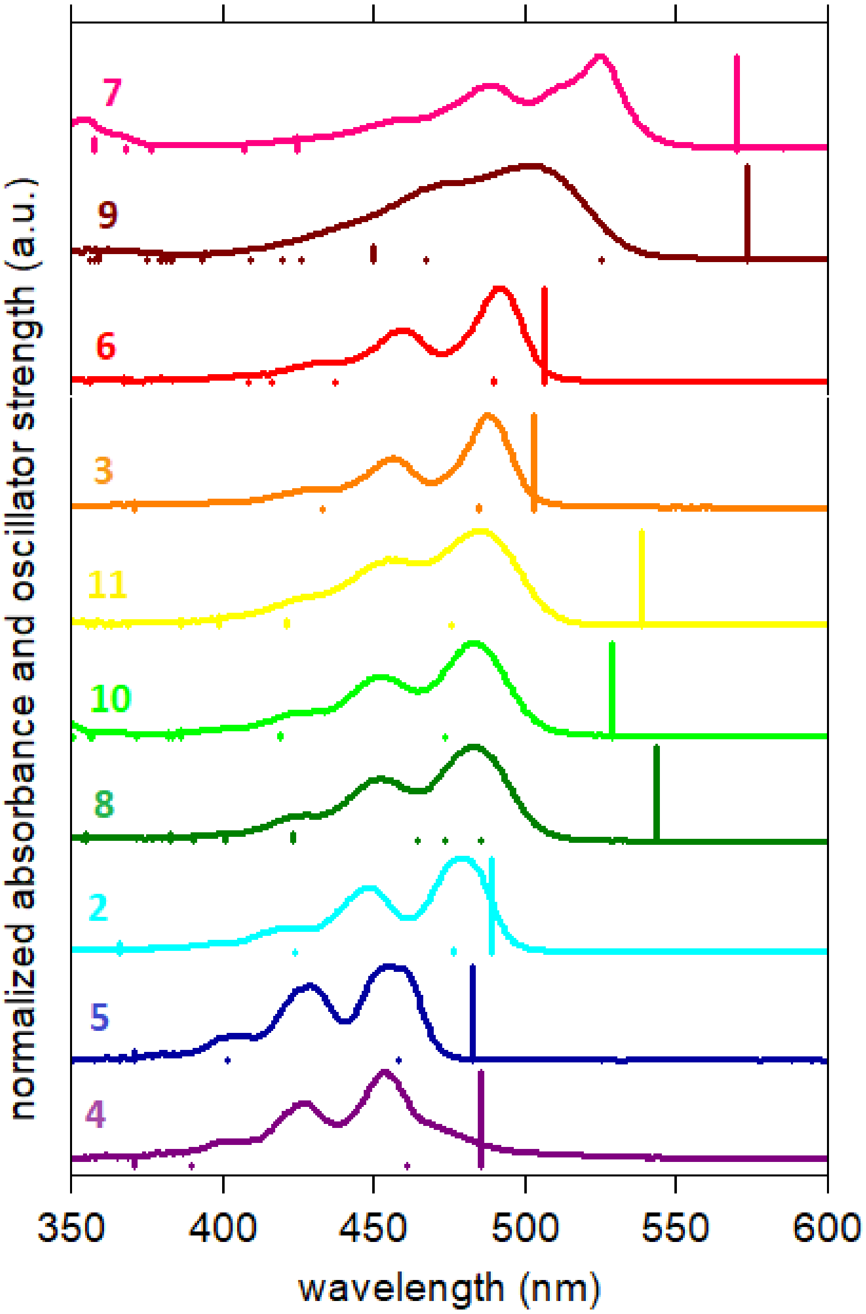

Figure S23. Comparison of normalized absorption spectra and TD-DFT results (vertical lines) of **2**-**11**.

Table S1. Characteristic main electron transition of **2**-**11**, calculated using TD-DFT (def2-TZVPP/PBE level of theory).

Compound	Transition energy (wavelength)	Oscillator strength	Description of main contributions
**4**	2.57 eV (482 nm)	1.139	HOMO→LUMO (c = -0.924), 0.853<br>HOMO-2→LUMO+2 (c = -0.268), 0.072<br>HOMO-1→LUMO+2 (c = 0.101), 0.010<br>HOMO-5→LUMO+3 (c = 0.107), 0.011
**5**	2.56 eV (485 nm)	1.163	HOMO→LUMO (c = -0.928), 0.862<br>HOMO-2→LUMO+1 (c = 0.274), 0.075<br>HOMO-3→LUMO+3 (c = 0.112), 0.012<br>HOMO-2→LUMO+2 (c = -0.112), 0.012
**2**	2.56 eV (485 nm)	1.074	HOMO→LUMO (c = -0.929); 0.862<br>HOMO-2→LUMO+1 (c = -0.267); 0.071<br>HOMO-1→LUMO+3 (c = 0.115); 0.013<br>HOMO-4→LUMO+2 (c = -0.109); 0.012
**8**	2.28 eV (543 nm)	1.478	HOMO→LUMO (c = 0.921), 0.849<br>HOMO→LUMO+9 (c = 0.272), 0.074<br>HOMO-1→LUMO+2 (c = -0.151), 0.023
**10**	2.35 eV (529 nm)	1.499	HOMO→LUMO (c = 0.933), 0.870<br>HOMO-2→LUMO+1 (c = -0.197), 0.039<br>HOMO→LUMO+5 (c = -0.135), 0.018<br>HOMO→LUMO+9 (c = 0.105), 0.011<br>HOMO-5→LUMO (c = -0.138), 0.018
**11**	2.30 eV (539 nm)	1.642	HOMO→LUMO (c = -0.933), 0.870<br>HOMO-2→LUMO+1 (c = -0.180), 0.032<br>HOMO-4→LUMO (c = -0.175), 0.031<br>HOMO→LUMO+7 (c = 0.146), 0.021
**3**	2.46 eV (503 nm)	1.642	HOMO→LUMO (c = 0.933), 0.870<br>HOMO-2→LUMO+1 (c = -0.238), 0.057<br>HOMO-1→LUMO+3 (c = -0.100), 0.010
**6**	2.45 eV (506 nm)	1.431	HOMO→LUMO (c = -0.930), 0.865<br>HOMO-2→LUMO+1 (c = 0.149), 0.022<br>HOMO-4→LUMO (c = -0.164), 0.027<br>HOMO-9→LUMO (c = -0.112), 0.013
**9**	2.16 eV (574 nm)	1.597	HOMO→LUMO (c = -0.936), 0.876<br>HOMO-4→LUMO (c = 0.191), 0.036<br>HOMO→LUMO+5 (c = 0.133), 0.018<br>HOMO-5→LUMO+2 (c = -0.108), 0.011<br>HOMO-2→LUMO+2 (c = 0.113), 0.013
**7**	2.17 eV (570 nm)	1.642	HOMO→LUMO (c = 0.947), 0.897<br>HOMO-2→LUMO+1 (c = -0.160), 0.026<br>HOMO-7→LUMO (c = -0.138), 0.019<br>HOMO-1→LUMO+1 (c = 0.103), 0.010

Optimized geometries (DFT)

Table S2. Cartesian coordinates (XYZ) of DFT optimized geometry of **2** (def2-TZVPP/B3LYP).

Si	2.608370000	4.397820000	8.486518000
C	2.979986000	6.897540000	7.511140000
O	2.915105000	5.551534000	7.309866000
O	5.288963000	9.360819000	8.899837000
Si	5.600571000	9.392234000	10.546673000
C	4.101384000	7.457623000	8.107524000
H	4.924170000	6.824877000	8.404552000
C	3.201827000	11.091554000	8.065129000
H	4.091987000	11.521472000	8.498880000
C	3.138618000	9.679322000	7.899688000
C	4.178392000	8.828790000	8.313380000
C	2.176896000	11.888044000	7.672968000
H	2.273337000	12.952934000	7.812122000
C	1.988152000	9.109780000	7.288196000
C	0.903936000	9.951667000	6.879758000
C	-0.251970000	9.374059000	6.278632000
C	-1.331072000	10.207906000	5.882354000
C	-0.279012000	7.957407000	6.089507000
H	-1.135784000	7.500308000	5.620800000
C	0.747692000	7.159261000	6.474327000
H	0.704939000	6.093522000	6.305587000
C	1.908425000	7.704481000	7.091513000
C	2.078947000	2.886142000	7.532929000
H	1.169805000	3.080487000	6.961752000
H	1.881316000	2.049735000	8.206448000
H	2.856125000	2.576383000	6.832203000
C	4.164863000	4.055047000	9.466272000
H	4.001000000	3.241236000	10.176343000
H	4.482644000	4.929545000	10.035533000
H	4.985757000	3.764215000	8.808214000
C	1.260294000	5.038894000	9.610245000
H	1.024123000	4.311739000	10.389871000
H	0.345902000	5.248146000	9.053299000
H	1.567801000	5.964728000	10.099949000
C	4.201226000	10.266958000	11.423863000
H	4.348561000	10.240601000	12.505791000
H	3.244785000	9.788127000	11.205830000
H	4.123812000	11.311248000	11.119487000
C	5.752453000	7.645169000	11.200790000
H	4.805387000	7.109951000	11.117135000
H	6.032701000	7.656348000	12.256436000
H	6.511601000	7.078535000	10.659074000
C	7.211595000	10.316868000	10.693010000
H	7.515888000	10.404893000	11.737947000
H	7.122811000	11.323972000	10.282559000
H	8.007934000	9.802753000	10.152473000
Si	-2.950968000	17.170195000	4.475170000
C	-3.322567000	14.670463000	5.450531000
O	-3.257690000	16.016468000	5.651814000
O	-5.631542000	12.207182000	4.061830000
Si	-5.943141000	12.175749000	2.414992000
C	-4.443964000	14.110380000	4.854143000
H	-5.266751000	14.743125000	4.557118000
C	-3.544405000	10.476449000	4.896535000
H	-4.434564000	10.046532000	4.462781000
C	-3.481198000	11.888681000	5.061979000
C	-4.520971000	12.739212000	4.648285000
C	-2.519475000	9.679960000	5.288697000
H	-2.615916000	8.615070000	5.149543000
C	-2.330733000	12.458223000	5.673472000
C	-1.246517000	11.616336000	6.081910000
C	-0.090611000	12.193944000	6.683037000

C	0.988492000	11.360098000	7.079314000
C	-0.063569000	13.610597000	6.872162000
H	0.793202000	14.067696000	7.340870000
C	-1.090274000	14.408742000	6.487343000
H	-1.047521000	15.474481000	6.656083000
C	-2.251006000	13.863522000	5.870156000
C	-2.421513000	18.681855000	5.428769000
H	-1.512361000	18.487493000	5.999926000
H	-2.223887000	19.518269000	4.755257000
H	-3.198674000	18.991612000	6.129514000
C	-4.507477000	17.512996000	3.495451000
H	-4.343621000	18.326819000	2.785393000
H	-4.825275000	16.638512000	2.926180000
H	-5.328356000	17.803823000	4.153530000
C	-1.602920000	16.529123000	3.351408000
H	-1.366757000	17.256286000	2.571787000
H	-0.688519000	16.319856000	3.908334000
H	-1.910445000	15.603298000	2.861699000
C	-4.543832000	11.300943000	1.537828000
H	-4.691159000	11.327286000	0.455898000
H	-3.587369000	11.779733000	1.755857000
H	-4.466468000	10.256655000	1.842224000
C	-6.094937000	13.922807000	1.760839000
H	-5.147848000	14.457984000	1.844494000
H	-6.375175000	13.911620000	0.705190000
H	-6.854066000	14.489487000	2.302534000
C	-7.554206000	11.251184000	2.268663000
H	-7.858493000	11.163147000	1.223725000
H	-7.465471000	10.244087000	2.679141000
H	-8.350527000	11.765349000	2.809181000

Table S3. Cartesian coordinates (XYZ) of DFT optimized geometry of **3** (def2-TZVPP/PBE0).

C	-5.052544000	-0.294465000	1.685597000
C	-3.662602000	-0.567911000	1.675995000
C	-2.742096000	0.523421000	1.677189000
C	-3.222898000	1.868877000	1.673486000
C	-4.620099000	2.093563000	1.653136000
C	-5.514001000	1.023601000	1.693017000
C	-3.157874000	-1.898646000	1.653893000
C	-1.812046000	-2.139288000	1.637492000
C	-0.851691000	-1.081476000	1.639229000
C	-1.329717000	0.268185000	1.656047000
C	-0.408841000	1.364464000	1.643412000
C	-0.937365000	2.691783000	1.660317000
C	-2.282469000	2.936975000	1.672107000
C	0.555062000	-1.335740000	1.618715000
C	1.476148000	-0.239456000	1.605008000
C	0.998062000	1.110291000	1.614525000
C	1.083254000	-2.663317000	1.612767000
C	2.427959000	-2.908618000	1.582748000
C	3.368149000	-1.840956000	1.555780000
C	2.888376000	-0.494988000	1.574815000
C	3.807964000	0.596292000	1.545950000
C	4.764160000	-2.065580000	1.501890000
C	5.657468000	-0.994236000	1.490511000
C	5.198212000	0.323902000	1.494080000
C	3.303412000	1.926642000	1.557364000
C	1.957978000	2.168102000	1.591939000
O	-5.100545000	3.365787000	1.631669000
O	-5.915672000	-1.347751000	1.685442000
O	5.243817000	-3.337205000	1.512398000
O	6.050377000	1.382732000	1.452890000
Si	-5.517526000	4.444976000	0.392221000

C	-7.412916000	4.346394000	0.226112000
C	-4.943475000	6.084651000	1.166498000
C	-4.567790000	4.062472000	-1.214785000
C	-4.479465000	2.578790000	-1.609815000
C	-5.119742000	4.879054000	-2.398276000
C	-5.735459000	6.394451000	2.447000000
C	-4.875309000	7.304991000	0.239151000
C	-7.893625000	3.078483000	-0.495688000
C	-8.074784000	5.592424000	-0.381878000
Si	-7.287740000	-1.539309000	0.696318000
C	-7.641353000	-3.405340000	0.787726000
C	-6.380397000	-4.257126000	0.995110000
C	-8.445491000	-3.911825000	-0.421644000
C	-8.738667000	-0.561213000	1.451883000
C	-8.684904000	-0.599260000	2.987207000
C	-10.113763000	-1.035050000	0.954705000
C	-6.782945000	-0.857257000	-1.002785000
C	-5.625840000	-1.647888000	-1.632434000
C	-7.934189000	-0.645596000	-1.994931000
Si	5.692179000	-4.481235000	0.348185000
C	7.595925000	-4.441236000	0.286457000
C	5.036034000	-6.077421000	1.147316000
C	4.833492000	-4.144679000	-1.317410000
C	4.760379000	-2.666753000	-1.732798000
C	5.454306000	-4.978567000	-2.452989000
C	8.250055000	-5.719745000	-0.259547000
C	8.160990000	-3.204083000	-0.426842000
C	5.755582000	-6.374365000	2.472300000
C	4.974205000	-7.319496000	0.248442000
Si	7.494758000	1.587168000	0.577873000
C	7.672040000	3.475412000	0.643001000
C	8.880423000	0.707479000	1.547197000
C	7.276780000	0.841292000	-1.160362000
C	5.855090000	0.995056000	-1.723547000
C	8.307850000	1.384108000	-2.165412000
C	6.568105000	4.198297000	-0.144055000
C	9.058258000	4.014221000	0.260698000
C	9.083446000	1.354515000	2.926008000
C	10.209319000	0.538504000	0.796768000
H	-6.582060000	1.231342000	1.722032000
H	-3.868164000	-2.723561000	1.651435000
H	-1.472154000	-3.172548000	1.619059000
H	-0.256518000	3.540212000	1.668794000
H	-2.653966000	3.960645000	1.697534000
H	0.402738000	-3.511602000	1.639319000
H	2.799723000	-3.932332000	1.593798000
H	6.724141000	-1.204832000	1.482259000
H	4.014633000	2.751060000	1.545891000
H	1.618061000	3.201470000	1.600570000
H	-7.739279000	4.275571000	1.280287000
H	-3.907236000	5.849048000	1.473303000
H	-3.538997000	4.412895000	-1.010634000
H	-3.896315000	2.472405000	-2.538631000
H	-3.994780000	1.964332000	-0.842806000
H	-5.474063000	2.152743000	-1.802911000
H	-4.468018000	4.762775000	-3.278463000
H	-5.200515000	5.952979000	-2.185433000
H	-6.118758000	4.523526000	-2.689285000
H	-5.299022000	7.256102000	2.976476000
H	-5.739583000	5.541600000	3.139293000
H	-6.781651000	6.649445000	2.221638000
H	-4.504984000	8.184089000	0.790468000
H	-4.197101000	7.141972000	-0.609346000
H	-5.860999000	7.571879000	-0.167828000
H	-8.985929000	2.964974000	-0.403263000
H	-7.429181000	2.166420000	-0.098130000
H	-7.663398000	3.118271000	-1.570098000

H	-9.171256000	5.483143000	-0.383531000
H	-7.837208000	6.506740000	0.177421000
H	-7.764460000	5.751016000	-1.424289000
H	-8.275473000	-3.527171000	1.685519000
H	-6.646242000	-5.323279000	1.075358000
H	-5.842968000	-3.970683000	1.907558000
H	-5.682203000	-4.155689000	0.151564000
H	-8.719631000	-4.969868000	-0.285857000
H	-9.374398000	-3.348363000	-0.582753000
H	-7.854090000	-3.847372000	-1.346279000
H	-8.610387000	0.486923000	1.126591000
H	-9.507404000	-0.008400000	3.420368000
H	-7.738704000	-0.206689000	3.382067000
H	-8.792864000	-1.628748000	3.362078000
H	-10.914300000	-0.408961000	1.379395000
H	-10.208127000	-0.995310000	-0.139030000
H	-10.313839000	-2.069956000	1.269421000
H	-6.385003000	0.142491000	-0.748400000
H	-5.256458000	-1.137482000	-2.536136000
H	-4.776486000	-1.754417000	-0.943637000
H	-5.942142000	-2.656866000	-1.935112000
H	-7.561686000	-0.176119000	-2.919608000
H	-8.715861000	0.010735000	-1.587962000
H	-8.409066000	-1.594509000	-2.282295000
H	7.863613000	-4.360286000	1.356407000
H	3.993671000	-5.803810000	1.396360000
H	3.797045000	-4.498799000	-1.164339000
H	4.260881000	-2.573176000	-2.710522000
H	4.200010000	-2.057031000	-1.015011000
H	5.760534000	-2.222869000	-1.835920000
H	4.852408000	-4.881862000	-3.370255000
H	5.526773000	-6.048138000	-2.215344000
H	6.466820000	-4.624085000	-2.694147000
H	9.347633000	-5.647792000	-0.194409000
H	7.946526000	-6.615120000	0.298373000
H	7.999432000	-5.886284000	-1.316607000
H	9.251864000	-3.134720000	-0.287165000
H	7.719876000	-2.269434000	-0.056418000
H	7.974959000	-3.246233000	-1.509802000
H	5.267691000	-7.206754000	3.003668000
H	5.753536000	-5.502443000	3.140625000
H	6.802889000	-6.666967000	2.305206000
H	4.540557000	-8.169624000	0.798939000
H	4.351895000	-7.154651000	-0.641703000
H	5.970512000	-7.632783000	-0.094562000
H	7.510922000	3.693031000	1.715165000
H	8.485281000	-0.308739000	1.721820000
H	7.471505000	-0.239141000	-1.032250000
H	5.775662000	0.490752000	-2.699748000
H	5.092716000	0.562090000	-1.064497000
H	5.597612000	2.051655000	-1.879380000
H	8.232545000	0.842796000	-3.121672000
H	9.343831000	1.291642000	-1.813134000
H	8.125141000	2.446568000	-2.382093000
H	6.592726000	5.280981000	0.057630000
H	5.565944000	3.830701000	0.114404000
H	6.701048000	4.068697000	-1.228077000
H	9.092483000	5.108291000	0.384888000
H	9.858467000	3.589241000	0.881067000
H	9.303434000	3.800553000	-0.789259000
H	9.783053000	0.762677000	3.536942000
H	8.138841000	1.435683000	3.481944000
H	9.505673000	2.366250000	2.837500000
H	10.940413000	0.006484000	1.426298000
H	10.087818000	-0.043687000	-0.127043000
H	10.658920000	1.504410000	0.526253000

Table S4. Cartesian coordinates (XYZ) of DFT optimized geometry of **4** (def2-TZVPP/B3LYP).

C	-4.905631000	-1.218815000	-0.262468000
C	-3.512537000	-1.260563000	-0.148458000
C	-2.806350000	-0.032812000	-0.027577000
C	-3.520854000	1.196086000	-0.021609000
C	-4.914514000	1.156850000	-0.131428000
C	-5.609328000	-0.030591000	-0.252809000
C	-2.778117000	-2.481231000	-0.149134000
C	-1.427199000	-2.479088000	-0.036760000
C	-0.673969000	-1.270315000	0.084323000
C	-1.381353000	-0.034017000	0.087659000
C	-0.681670000	1.201080000	0.204865000
C	-1.442348000	2.411219000	0.200852000
C	-2.793779000	2.415639000	0.094822000
C	0.740832000	-1.271266000	0.198713000
C	1.440702000	-0.036298000	0.315063000
C	0.733023000	1.199643000	0.320507000
C	1.500898000	-2.481653000	0.204101000
C	2.851777000	-2.486075000	0.310852000
C	3.580179000	-1.267173000	0.421127000
C	2.866375000	-0.037225000	0.426445000
C	3.572472000	1.191687000	0.542184000
C	2.836157000	2.410694000	0.553641000
C	1.485304000	2.408280000	0.446779000
C	4.973220000	-1.227919000	0.526531000
C	5.667175000	-0.038819000	0.633958000
C	4.965987000	1.150817000	0.642303000
O	-5.576473000	-2.419664000	-0.454772000
O	-5.603584000	2.359710000	-0.048490000
O	5.675546000	2.334230000	0.818849000
O	5.687529000	-2.420026000	0.588123000
C	-6.324141000	2.796501000	-1.131346000
C	-7.021494000	4.114578000	-0.813278000
C	6.058347000	3.030518000	-0.302194000
C	6.812451000	4.298710000	0.082646000
C	-6.441347000	-2.865309000	0.512342000
C	-7.091757000	-4.176531000	0.085365000
C	6.104260000	-2.984178000	-0.593398000
C	6.855681000	-4.285305000	-0.333686000
O	-6.637451000	-2.273073000	1.534797000
O	-6.379838000	2.192953000	-2.164316000
O	5.808465000	2.657887000	-1.411452000
O	5.881485000	-2.489573000	-1.659968000
C	5.904465000	-5.276603000	0.360318000
C	7.326515000	-4.857262000	-1.672487000
C	8.063331000	-3.996885000	0.574846000
C	5.881414000	5.195953000	0.917521000
C	8.050364000	3.915321000	0.911970000
C	7.237139000	5.025434000	-1.195116000
C	-5.977994000	5.149930000	-0.359928000
C	-7.741702000	4.607081000	-2.070204000
C	-8.036727000	3.865751000	0.317254000
C	-5.998903000	-5.209641000	-0.238076000
C	-7.975705000	-4.683249000	1.226835000
C	-7.945036000	-3.908286000	-1.167960000
H	-6.684297000	-0.030250000	-0.339474000
H	-3.316128000	-3.412261000	-0.243852000
H	-0.912961000	-3.426684000	-0.041752000
H	-0.933590000	3.358170000	0.283721000
H	-3.338176000	3.347733000	0.098583000
H	0.991991000	-3.428459000	0.120853000
H	3.391570000	-3.420843000	0.316602000
H	3.369531000	3.343925000	0.652528000
H	0.970347000	3.355388000	0.458731000
H	6.744417000	-0.039007000	0.709775000
H	6.425273000	-6.219752000	0.530455000

H	5.563679000	-4.893683000	1.320647000
H	5.032243000	-5.483976000	-0.261758000
H	7.863261000	-5.791077000	-1.501217000
H	7.992003000	-4.163819000	-2.184814000
H	6.484661000	-5.056749000	-2.333873000
H	7.748992000	-3.607082000	1.541104000
H	8.621610000	-4.919521000	0.738780000
H	8.737348000	-3.273963000	0.112804000
H	6.404942000	6.116046000	1.180337000
H	4.989146000	5.469235000	0.351862000
H	5.572451000	4.701199000	1.836576000
H	8.610742000	4.816613000	1.163947000
H	8.711077000	3.255694000	0.347407000
H	7.769358000	3.413820000	1.836092000
H	6.373344000	5.293374000	-1.801797000
H	7.775113000	5.937482000	-0.933963000
H	7.888525000	4.401302000	-1.805304000
H	-6.472467000	6.104194000	-0.174189000
H	-5.480733000	4.837414000	0.556298000
H	-5.221170000	5.308328000	-1.129791000
H	-8.254388000	5.544607000	-1.852296000
H	-8.476905000	3.880872000	-2.413318000
H	-7.039711000	4.777962000	-2.885500000
H	-7.543573000	3.518609000	1.223444000
H	-8.561879000	4.794726000	0.543253000
H	-8.779090000	3.123193000	0.021061000
H	-6.465768000	-6.159047000	-0.503174000
H	-5.381449000	-4.885997000	-1.073733000
H	-5.353597000	-5.382469000	0.624694000
H	-8.456027000	-5.615903000	0.928920000
H	-8.749167000	-3.959129000	1.477894000
H	-7.390274000	-4.867703000	2.126834000
H	-7.333498000	-3.551724000	-1.994862000
H	-8.437591000	-4.831818000	-1.475050000
H	-8.718474000	-3.166275000	-0.964360000

Table S5. Cartesian coordinates (XYZ) of DFT optimized geometry of **5** (def2-TZVPP/B3LYP).

S	2.124299000	1.804740000	1.812283000
C	0.321105000	4.549547000	5.186763000
F	1.394541000	2.402326000	-0.621337000
O	1.192641000	1.679463000	3.116137000
O	2.536141000	0.486527000	1.472925000
F	0.280115000	3.463820000	0.910997000
C	1.251919000	2.640860000	4.139243000
S	-0.415810000	7.011726000	4.668199000
C	0.297564000	3.636906000	4.152560000
H	-0.444633000	3.702568000	3.373654000
F	-0.136924000	1.370023000	0.521370000
O	3.022708000	2.902221000	1.944042000
O	-0.684919000	5.529686000	5.229216000
C	2.230085000	2.513950000	5.129018000
F	-0.958499000	5.986990000	2.297230000
O	-1.081833000	7.911002000	5.545795000
F	-1.392898000	8.104364000	2.508679000
C	3.228248000	3.387130000	7.212089000
O	0.945374000	7.157075000	4.274496000
F	-2.706035000	6.629241000	3.412276000
C	2.237211000	3.466958000	6.185930000
C	3.231486000	4.345067000	8.265171000
C	4.280600000	5.214556000	10.350341000
H	3.563104000	6.017719000	10.392678000
C	4.221502000	4.271186000	9.279555000
C	5.218247000	5.144826000	11.325989000
H	5.233015000	5.885103000	12.111705000
C	2.220438000	5.354023000	8.265537000

H	2.192518000	6.079642000	9.061997000
C	1.277442000	5.434683000	7.295897000
H	0.524888000	6.207536000	7.340374000
C	1.260819000	4.501759000	6.219821000
C	0.818012000	2.291672000	0.569800000
C	-1.445950000	6.923153000	3.113127000
S	6.300909000	4.817533000	14.638306000
C	8.104097000	2.072726000	11.263825000
F	7.030680000	4.219959000	17.071926000
O	7.232563000	4.942812000	13.334449000
O	5.889059000	6.135743000	14.977661000
F	8.145110000	3.158470000	15.539591000
C	7.173285000	3.981413000	12.311344000
S	8.841007000	-0.389454000	11.782387000
C	8.127639000	2.985368000	12.298028000
H	8.869836000	2.919706000	13.076934000
F	8.562132000	5.252273000	15.929212000
O	5.402509000	3.720044000	14.506554000
O	9.110121000	1.092586000	11.221373000
C	6.195118000	4.108323000	11.321569000
F	9.383681000	0.635276000	14.153361000
O	9.507035000	-1.288729000	10.904793000
F	9.818082000	-1.482097000	13.941910000
C	5.196955000	3.235142000	9.238499000
O	7.479820000	-0.534804000	12.176081000
F	11.131224000	-0.006972000	13.038325000
C	6.187992000	3.155314000	10.264657000
C	5.193718000	2.277205000	8.185415000
C	4.144603000	1.407716000	6.100247000
H	4.862097000	0.604552000	6.057910000
C	4.203700000	2.351086000	7.171032000
C	3.206955000	1.477446000	5.124598000
H	3.192188000	0.737170000	4.338882000
C	6.204766000	1.268250000	8.185050000
H	6.232687000	0.542632000	7.388589000
C	7.147762000	1.187590000	9.154690000
H	7.900316000	0.414738000	9.110213000
C	7.164384000	2.120514000	10.230766000
C	7.607205000	4.330615000	15.880787000
C	9.871137000	-0.300885000	13.337465000

Table S6. Cartesian coordinates (XYZ) of DFT optimized geometry of **6** (def2-TZVPP/B3LYP).

P	2.200354000	1.511765000	7.288896000
O	2.601224000	0.811697000	5.806669000
C	3.725072000	-1.303894000	-0.151452000
P	4.205975000	5.840895000	4.791086000
O	4.984172000	4.584167000	3.977852000
C	3.623246000	-0.629520000	1.094863000
C	2.982837000	-1.220401000	2.227219000
H	2.575302000	-2.215751000	2.147255000
C	2.866421000	-0.570642000	3.412878000
H	2.377959000	-1.047297000	4.249182000
C	3.381605000	0.745548000	3.582503000
C	3.248252000	1.457813000	4.789736000
C	3.769773000	2.737309000	4.926251000
H	3.644991000	3.277981000	5.851519000
C	4.600628000	2.667498000	2.640068000
C	4.433818000	3.333849000	3.864149000
C	5.287366000	3.256279000	1.541341000
H	5.725101000	4.234196000	1.672382000
C	5.400580000	2.604278000	0.357469000
H	5.934239000	3.088522000	-0.444575000
C	4.055666000	1.362864000	2.493804000
C	4.177470000	0.675033000	1.242703000
C	3.430436000	0.596817000	8.399589000

C	4.820505000	0.911349000	7.817767000
H	5.583702000	0.521807000	8.494871000
H	4.985613000	1.984480000	7.710895000
H	4.964612000	0.445672000	6.844206000
C	3.350892000	1.209338000	9.806512000
H	4.104257000	0.740446000	10.444002000
H	2.381487000	1.050301000	10.275988000
H	3.551160000	2.281807000	9.790650000
C	3.274391000	-0.920442000	8.484686000
H	4.127778000	-1.343575000	9.021615000
H	3.241938000	-1.376817000	7.496376000
H	2.377453000	-1.206896000	9.031052000
C	0.491317000	0.719671000	7.432897000
C	0.000560000	0.855676000	8.879251000
H	-1.053867000	0.571029000	8.932046000
H	0.090995000	1.878983000	9.249166000
H	0.546662000	0.197962000	9.553266000
C	0.366715000	-0.741522000	6.988761000
H	-0.690359000	-1.023278000	7.004204000
H	0.891402000	-1.423112000	7.649168000
H	0.739400000	-0.885540000	5.976631000
C	-0.389531000	1.582639000	6.508255000
H	-1.427401000	1.245899000	6.569840000
H	-0.071907000	1.494315000	5.468524000
H	-0.356087000	2.636776000	6.785797000
C	5.748456000	6.571852000	5.597931000
C	5.984710000	5.652545000	6.811610000
H	6.838743000	6.016288000	7.388104000
H	6.206132000	4.631696000	6.496188000
H	5.116141000	5.626887000	7.470854000
C	5.447554000	7.992244000	6.091904000
H	6.233922000	8.310274000	6.780165000
H	4.498690000	8.055319000	6.628655000
H	5.428249000	8.705083000	5.269017000
C	7.018415000	6.585390000	4.739336000
H	7.861271000	6.911575000	5.355737000
H	6.942238000	7.275104000	3.903870000
H	7.245114000	5.595783000	4.345143000
C	3.651888000	6.891571000	3.323838000
C	4.734978000	7.509828000	2.439220000
H	4.266652000	7.988604000	1.574359000
H	5.429619000	6.758396000	2.065343000
H	5.304769000	8.273677000	2.966068000
C	2.787779000	5.947015000	2.468059000
H	2.263995000	6.535388000	1.710791000
H	2.038600000	5.426955000	3.067776000
H	3.390405000	5.201084000	1.955482000
C	2.737369000	7.989465000	3.888780000
H	2.258941000	8.522665000	3.062887000
H	3.283284000	8.720348000	4.483536000
H	1.951080000	7.563287000	4.513722000
P	6.370907000	-1.510766000	-7.288659000
O	5.969530000	-0.811555000	-5.806280000
C	4.844564000	1.303435000	0.151141000
P	4.367574000	-5.841696000	-4.791599000
O	3.589073000	-4.585650000	-3.977660000
C	4.945928000	0.629278000	-1.095303000
C	5.585763000	1.220605000	-2.227723000
H	5.992413000	2.216317000	-2.147967000
C	5.702372000	0.570907000	-3.413352000
H	6.190201000	1.047886000	-4.249828000
C	5.188247000	-0.745707000	-3.582645000
C	5.322633000	-1.458098000	-4.789620000
C	4.802432000	-2.738114000	-4.926015000
H	4.928225000	-3.278869000	-5.851108000
C	3.970591000	-2.668415000	-2.640183000
C	4.138426000	-3.334938000	-3.864075000

C	3.283887000	-3.257334000	-1.541496000
H	2.846828000	-4.235573000	-1.672391000
C	3.169715000	-2.605042000	-0.357823000
H	2.636116000	-3.089472000	0.444165000
C	4.514527000	-1.363368000	-2.493985000
C	4.392135000	-0.675446000	-1.243012000
C	5.141294000	-0.595081000	-8.399235000
C	3.751081000	-0.909824000	-7.817896000
H	2.988006000	-0.520782000	-8.495422000
H	3.586241000	-1.982956000	-7.710597000
H	3.606523000	-0.443816000	-6.844559000
C	5.221305000	-1.206929000	-9.806428000
H	4.468179000	-0.737719000	-10.443962000
H	6.190886000	-1.047665000	-10.275472000
H	5.021026000	-2.279404000	-9.791141000
C	5.297352000	0.922210000	-8.483637000
H	4.444412000	1.345456000	-9.021193000
H	5.328857000	1.378239000	-7.495139000
H	6.194768000	1.208922000	-9.029089000
C	8.079995000	-0.718552000	-7.431405000
C	8.571233000	-0.853195000	-8.877723000
H	9.625633000	-0.568347000	-8.929989000
H	8.481094000	-1.876213000	-9.248507000
H	8.025233000	-0.195019000	-9.551368000
C	8.204273000	0.742208000	-6.985734000
H	9.261299000	1.024164000	-7.000762000
H	7.679502000	1.424409000	-7.645437000
H	7.831503000	0.885076000	-5.973479000
C	8.960641000	-1.582315000	-6.507299000
H	9.998486000	-1.245370000	-6.568173000
H	8.642652000	-1.495079000	-5.467586000
H	8.927462000	-2.636176000	-6.785899000
C	2.825317000	-6.572727000	-5.598750000
C	2.588819000	-5.653054000	-6.812091000
H	1.734551000	-6.016512000	-7.388413000
H	2.367668000	-4.632237000	-6.496376000
H	3.457192000	-5.627432000	-7.471599000
C	3.126688000	-7.992834000	-6.093280000
H	2.340656000	-8.310725000	-6.781987000
H	4.075758000	-8.055473000	-6.629723000
H	3.145887000	-8.706027000	-5.270699000
C	1.555365000	-6.586938000	-4.740139000
H	0.712602000	-6.913203000	-5.356617000
H	1.631747000	-7.276826000	-3.904834000
H	1.328368000	-5.597499000	-4.345687000
C	4.921907000	-6.892909000	-3.324792000
C	3.839025000	-7.512314000	-2.440713000
H	4.307547000	-7.991431000	-1.576147000
H	3.143955000	-6.761509000	-2.066371000
H	3.269713000	-8.276120000	-2.968133000
C	5.785259000	-5.948352000	-2.468284000
H	6.309113000	-6.536813000	-1.711135000
H	6.534357000	-5.427605000	-3.067508000
H	5.182056000	-5.202979000	-1.955551000
C	5.837096000	-7.990015000	-3.890172000
H	6.315594000	-8.523472000	-3.064484000
H	5.291646000	-8.720818000	-4.485460000
H	6.623322000	-7.563102000	-4.514694000

Table S7. Cartesian coordinates (XYZ) of DFT optimized geometry of **7** (def2-TZVPP/B3LYP).

C	-5.312621000	-1.215801000	-0.006426000
C	-3.894467000	-1.228958000	-0.007143000
C	-3.183023000	0.000774000	-0.008488000
C	-3.895220000	1.230071000	-0.009087000
C	-5.313366000	1.216046000	-0.008332000
C	-5.993519000	-0.000086000	-0.007165000

C	-3.157470000	-2.443613000	-0.006437000
C	-1.799839000	-2.442206000	-0.007170000
C	-1.042947000	-1.233644000	-0.008651000
C	-1.751216000	0.001213000	-0.009249000
C	-1.043702000	1.236502000	-0.010607000
C	-1.801335000	2.444601000	-0.011082000
C	-3.158967000	2.445177000	-0.010351000
C	0.379009000	-1.233195000	-0.009537000
C	1.086596000	0.002078000	-0.010972000
C	0.378253000	1.236920000	-0.011463000
C	1.136586000	-2.441298000	-0.009074000
C	2.494183000	-2.441781000	-0.010019000
C	3.230611000	-1.226861000	-0.011523000
C	2.518460000	0.002516000	-0.011947000
C	3.229853000	1.232329000	-0.013383000
C	2.492682000	2.446800000	-0.013765000
C	1.135086000	2.445487000	-0.012855000
C	4.648691000	-1.212877000	-0.012568000
C	5.328695000	0.003381000	-0.014043000
C	4.647941000	1.219218000	-0.014342000
C	-6.047127000	-2.429765000	-0.004821000
C	-6.048617000	2.429560000	-0.008631000
C	5.384500000	2.432063000	-0.015569000
C	5.386024000	-2.425254000	-0.012155000
C	-6.669872000	-3.471074000	-0.000809000
C	-6.672010000	3.470487000	-0.006580000
C	6.014599000	3.468981000	-0.014165000
C	6.016848000	-3.461731000	-0.010080000
Si	-7.596387000	-5.053937000	0.008110000
C	-8.686792000	-5.099664000	1.532368000
C	-6.351403000	-6.455921000	0.047571000
C	-8.639905000	-5.145510000	-1.546628000
Si	-7.599476000	5.052807000	-0.000890000
C	-6.355187000	6.455754000	0.023164000
C	-8.680608000	5.106504000	1.529708000
C	-8.652512000	5.134923000	-1.549721000
Si	6.964159000	5.038451000	-0.013214000
C	6.521816000	6.000330000	-1.560947000
C	8.792086000	4.624632000	0.004933000
C	6.494439000	6.014632000	1.517409000
Si	6.967465000	-5.030563000	-0.008355000
C	8.794723000	-4.615706000	0.035451000
C	6.545180000	-5.981849000	-1.568186000
C	6.479417000	-6.017778000	1.509409000
H	-7.073398000	-0.000416000	-0.006701000
H	-3.702987000	-3.376381000	-0.005260000
H	-1.288252000	-3.391387000	-0.006572000
H	-1.290332000	3.394096000	-0.012044000
H	-3.705055000	3.377610000	-0.010707000
H	0.625483000	-3.390735000	-0.007958000
H	3.040415000	-3.374087000	-0.009624000
H	3.038342000	3.379439000	-0.014802000
H	0.623400000	3.394609000	-0.013211000
H	6.408587000	0.003713000	-0.014884000
H	-9.396505000	-4.270964000	1.530250000
H	-9.255379000	-6.031390000	1.572481000
H	-8.090355000	-5.027718000	2.443050000
H	-5.698539000	-6.424055000	-0.826167000
H	-6.858067000	-7.423391000	0.054524000
H	-5.724560000	-6.397613000	0.938805000
H	-9.206842000	-6.078629000	-1.576587000
H	-9.349491000	-4.317871000	-1.590593000
H	-8.016005000	-5.099796000	-2.440450000
H	-5.707489000	6.419309000	-0.854230000
H	-6.862431000	7.422935000	0.027630000
H	-5.723037000	6.402918000	0.910988000
H	-9.389874000	4.277448000	1.536568000

H	-9.249442000	6.038154000	1.568064000
H	-8.078595000	5.039970000	2.437126000
H	-8.034046000	5.084618000	-2.447066000
H	-9.361738000	4.306546000	-1.584689000
H	-9.220319000	6.067453000	-1.581464000
H	5.454497000	6.225099000	-1.591096000
H	7.066975000	6.946136000	-1.596674000
H	6.769972000	5.432732000	-2.458995000
H	9.053369000	4.046011000	0.892249000
H	9.394544000	5.535608000	0.005967000
H	9.068989000	4.037707000	-0.872135000
H	5.426791000	6.239698000	1.526431000
H	6.726446000	5.455386000	2.424963000
H	7.039010000	6.960736000	1.554096000
H	9.044552000	-4.043229000	0.930021000
H	9.397787000	-5.526279000	0.037490000
H	9.082065000	-4.022393000	-0.833930000
H	5.478480000	-6.207047000	-1.613243000
H	7.091429000	-6.927035000	-1.603718000
H	6.804128000	-5.407809000	-2.459064000
H	7.024261000	-6.963714000	1.546298000
H	6.699572000	-5.464736000	2.423691000
H	5.411930000	-6.243721000	1.503469000

Table S8. Cartesian coordinates (XYZ) of DFT optimized geometry of **8** (def2-TZVPP/B3LYP).

C	-4.982508000	-1.204882000	0.210210000
C	-3.571805000	-1.224633000	0.172390000
C	-2.855156000	0.006644000	0.146132000
C	-3.565775000	1.241556000	0.120979000
C	-4.976648000	1.228888000	0.086023000
C	-5.648392000	0.013577000	0.148630000
C	-2.825296000	-2.439389000	0.116311000
C	-1.470279000	-2.438888000	0.098188000
C	-0.712944000	-1.231021000	0.137037000
C	-1.421896000	0.002963000	0.145258000
C	-0.706679000	1.233327000	0.152560000
C	-1.457909000	2.445004000	0.191679000
C	-2.812937000	2.452455000	0.175323000
C	0.706939000	-1.234635000	0.151110000
C	1.422149000	-0.004268000	0.143745000
C	0.713189000	1.229721000	0.136975000
C	1.458210000	-2.446334000	0.188744000
C	2.813219000	-2.453776000	0.170937000
C	3.565999000	-1.242846000	0.116471000
C	2.855409000	-0.007948000	0.143088000
C	3.572088000	1.223312000	0.169278000
C	2.825521000	2.438100000	0.114694000
C	1.470485000	2.437610000	0.098009000
C	4.976834000	-1.230160000	0.080002000
C	5.648647000	-0.014886000	0.142589000
C	4.982830000	1.203539000	0.205576000
C	-5.787692000	-2.444214000	0.309084000
C	-5.777748000	2.471251000	-0.009340000
C	5.788111000	2.442821000	0.304269000
C	5.777819000	-2.472471000	-0.016987000
C	-6.788038000	2.732430000	0.917025000
C	-7.565638000	3.879096000	0.813566000
C	-7.355510000	4.786868000	-0.215811000
C	-6.352828000	4.530128000	-1.141916000
C	-5.573831000	3.384596000	-1.044733000
C	5.586795000	3.352243000	1.344071000
C	6.370357000	4.493641000	1.447350000
C	7.372421000	4.752653000	0.520702000
C	7.581495000	3.847749000	-0.511030000
C	6.799914000	2.703938000	-0.619943000

C	-5.585314000	-3.354183000	1.348198000
C	-6.368778000	-4.495631000	1.451679000
C	-7.371795000	-4.754147000	0.525926000
C	-7.581934000	-3.848690000	-0.505106000
C	-6.800461000	-2.704825000	-0.614215000
C	6.789138000	-2.734220000	0.908100000
C	7.566597000	-3.880836000	0.803090000
C	7.355308000	-4.787992000	-0.226598000
C	6.351606000	-4.530679000	-1.151433000
C	5.572737000	-3.385190000	-1.052697000
C	-6.118728000	5.471481000	-2.294224000
F	-6.692922000	6.671459000	-2.099125000
F	-6.617332000	4.974263000	-3.447437000
F	-4.801664000	5.684079000	-2.501645000
C	-8.667451000	4.108496000	1.814926000
F	-8.287522000	3.767545000	3.063039000
F	-9.758014000	3.364270000	1.523513000
F	-9.064601000	5.392883000	1.851457000
C	-6.086001000	-5.476932000	2.558554000
F	-5.794138000	-4.854172000	3.717917000
F	-5.022319000	-6.258543000	2.259216000
F	-7.124589000	-6.298284000	2.791766000
C	-8.690359000	-4.075037000	-1.499818000
F	-9.090823000	-5.358508000	-1.534178000
F	-8.316781000	-3.734812000	-2.749955000
F	-9.777456000	-3.328340000	-1.201864000
C	8.669542000	-4.110870000	1.803058000
F	8.291199000	-3.770255000	3.051741000
F	9.759958000	-3.366837000	1.510598000
F	9.066395000	-5.395378000	1.838663000
C	6.116139000	-5.471345000	-2.304023000
F	6.691041000	-6.671248000	-2.110591000
F	6.612837000	-4.973165000	-3.457642000
F	4.798837000	-5.684270000	-2.509624000
C	8.688881000	4.074621000	-1.506780000
F	9.089498000	5.358054000	-1.540693000
F	8.313903000	3.735308000	-2.756746000
F	9.776186000	3.327555000	-1.210513000
C	6.088714000	5.474357000	2.555032000
F	5.797983000	4.850990000	3.714353000
F	5.024762000	6.256166000	2.257166000
F	7.127560000	6.295551000	2.787651000
H	-6.730314000	0.016195000	0.149758000
H	-3.353076000	-3.379398000	0.067274000
H	-0.961187000	-3.387340000	0.037865000
H	-0.943930000	3.390908000	0.250504000
H	-3.335776000	3.395206000	0.223928000
H	0.944293000	-3.392271000	0.247585000
H	3.336111000	-3.396553000	0.218451000
H	3.353249000	3.378139000	0.065635000
H	0.961331000	3.386096000	0.038764000
H	6.730570000	-0.017503000	0.142542000
H	-6.951841000	2.045173000	1.734961000
H	-7.954567000	5.680722000	-0.289869000
H	-4.807015000	3.193483000	-1.781711000
H	4.824912000	3.156145000	2.084607000
H	7.978755000	5.641035000	0.602556000
H	6.964564000	2.017608000	-1.438489000
H	-4.822665000	-3.158484000	2.088049000
H	-7.978048000	-5.642571000	0.607932000
H	-6.965951000	-2.018058000	-1.432224000
H	6.953862000	-2.047456000	1.726263000
H	7.954266000	-5.681812000	-0.301859000
H	4.805103000	-3.193619000	-1.788705000

Table S9. Cartesian coordinates (XYZ) of DFT optimized geometry of **9** (def2-TZVPP/B3LYP).

C	-5.075817000	-1.128242000	-0.001286000
C	-3.662261000	-1.147440000	-0.030449000
C	-2.945376000	0.082683000	-0.050028000
C	-3.655470000	1.317358000	-0.052116000
C	-5.069051000	1.310140000	-0.029100000
C	-5.743579000	0.093470000	0.021817000
C	-2.916541000	-2.362603000	-0.001208000
C	-1.560232000	-2.362731000	-0.002422000
C	-0.801727000	-1.155175000	-0.025776000
C	-1.511995000	0.078430000	-0.046810000
C	-0.798640000	1.309488000	-0.048300000
C	-1.549387000	2.521321000	-0.033952000
C	-2.904453000	2.528762000	-0.033124000
C	0.619614000	-1.156308000	-0.006917000
C	1.331896000	0.076069000	-0.013842000
C	0.620605000	1.308292000	-0.040527000
C	1.376714000	-2.364209000	0.040049000
C	2.733220000	-2.366477000	0.049831000
C	3.481894000	-1.153140000	0.012968000
C	2.764936000	0.077709000	0.010657000
C	3.474255000	1.312268000	0.036885000
C	2.727378000	2.524414000	-0.028938000
C	1.373045000	2.518938000	-0.065540000
C	4.895551000	-1.133756000	0.016540000
C	5.561365000	0.088614000	0.061663000
C	4.886029000	1.304605000	0.090463000
C	-5.853764000	-2.375899000	-0.015373000
C	-5.830128000	2.567447000	-0.077460000
C	5.645126000	2.562167000	0.156296000
C	5.707390000	-2.360340000	0.007926000
S	-6.972393000	-2.745486000	1.259295000
C	-7.428294000	-4.228355000	0.523595000
C	-6.754481000	-4.433993000	-0.646715000
C	-5.854276000	-3.379028000	-0.951623000
C	-5.786115000	3.559444000	-1.024902000
C	-6.652657000	4.647910000	-0.743710000
C	-7.348123000	4.478775000	0.419831000
S	-6.954128000	2.988879000	1.177040000
S	6.796320000	2.977669000	-1.075390000
C	7.168317000	4.474339000	-0.320932000
C	6.445319000	4.649998000	0.824739000
C	5.574880000	3.561207000	1.094001000
C	6.672385000	-2.722936000	0.912211000
C	7.328966000	-3.939559000	0.584132000
C	6.866087000	-4.487390000	-0.576831000
S	5.626327000	-3.526369000	-1.278872000
H	-6.824963000	0.096047000	0.026869000
H	-3.452775000	-3.297819000	0.039579000
H	-1.049925000	-3.312087000	0.028703000
H	-1.033925000	3.467493000	-0.001830000
H	-3.434931000	3.467072000	0.002750000
H	0.864918000	-3.312585000	0.077684000
H	3.263322000	-3.304843000	0.097800000
H	3.261723000	3.460760000	-0.062614000
H	0.860015000	3.464894000	-0.127675000
H	6.642404000	0.089187000	0.078846000
H	-8.162094000	-4.863267000	0.990666000
H	-6.902011000	-5.301934000	-1.272120000
H	-5.239700000	-3.344962000	-1.838965000
H	-5.157763000	3.496845000	-1.901067000
H	-6.760080000	5.513617000	-1.380233000
H	-8.066722000	5.143020000	0.869230000
H	7.896188000	5.136486000	-0.758320000
H	6.534694000	5.521191000	1.456399000
H	4.922581000	3.505060000	1.953023000

H	6.888739000	-2.137379000	1.793804000
H	8.101420000	-4.390917000	1.188791000
H	7.182055000	-5.399538000	-1.054917000

Table S10. Cartesian coordinates (XYZ) of DFT optimized geometry of **10** (def2-TZVPP/B3LYP).

C	8.633701000	3.222634000	7.108273000
H	9.103084000	3.619070000	7.998613000
C	8.090471000	1.946840000	7.156921000
C	7.476691000	1.415002000	6.005649000
C	6.928573000	0.098944000	5.978005000
H	7.011757000	-0.516868000	6.860991000
C	6.321082000	-0.391099000	4.869631000
H	5.935871000	-1.398249000	4.898749000
C	6.207930000	0.371428000	3.669122000
C	5.525657000	-0.119379000	2.523455000
C	4.786100000	-1.338854000	2.547313000
H	4.703663000	-1.893261000	3.469097000
C	4.147757000	-1.811862000	1.448466000
H	3.578332000	-2.726809000	1.511945000
C	4.191474000	-1.116715000	0.203700000
C	3.519891000	-1.590711000	-0.942058000
C	3.533273000	-0.827925000	-2.100613000
H	2.980504000	-1.186447000	-2.958124000
C	4.225891000	0.377690000	-2.205562000
C	4.945679000	0.849934000	-1.083740000
C	5.759489000	2.023766000	-1.124316000
H	5.910133000	2.522626000	-2.068789000
C	6.356307000	2.514780000	-0.010374000
H	6.967616000	3.399946000	-0.098146000
C	6.236451000	1.869340000	1.255680000
C	6.792258000	2.423187000	2.436971000
C	7.307034000	3.751997000	2.477899000
H	7.218829000	4.380273000	1.605082000
C	7.856937000	4.275075000	3.601274000
H	8.194336000	5.300170000	3.600456000
C	7.985600000	3.506340000	4.797548000
C	8.591797000	4.016458000	5.965362000
C	7.429599000	2.193799000	4.813716000
C	6.809107000	1.660711000	3.637874000
C	5.557563000	0.621329000	1.307854000
C	4.899300000	0.116638000	0.139220000
C	8.175009000	1.186908000	8.429069000
C	9.414321000	0.787348000	8.931612000
H	10.308487000	1.000751000	8.360719000
C	9.505212000	0.112079000	10.142942000
H	10.472728000	-0.194291000	10.518857000
C	8.355359000	-0.170462000	10.871006000
H	8.424096000	-0.691426000	11.816504000
C	7.115775000	0.220955000	10.378236000
H	6.216634000	0.006476000	10.940738000
C	7.025912000	0.893737000	9.166667000
H	6.062072000	1.208750000	8.789147000
C	2.786622000	-2.881347000	-0.938803000
C	3.471484000	-4.089200000	-0.781104000
H	4.545673000	-4.073523000	-0.652972000
C	2.789583000	-5.298683000	-0.796414000
H	3.337814000	-6.224281000	-0.680282000
C	1.408386000	-5.320436000	-0.959933000
H	0.875212000	-6.261633000	-0.966508000
C	0.717037000	-4.125179000	-1.120390000
H	-0.357154000	-4.133247000	-1.250074000
C	1.402319000	-2.915849000	-1.117521000
H	0.861583000	-1.986592000	-1.241408000
C	4.174925000	1.105040000	-3.494801000
C	4.360738000	0.410920000	-4.696031000
H	4.577244000	-0.648580000	-4.665062000

C	4.297138000	1.065015000	-5.918758000
H	4.452518000	0.506984000	-6.832595000
C	4.049748000	2.431947000	-5.969211000
H	4.007301000	2.945371000	-6.920376000
C	3.848574000	3.133170000	-4.785545000
H	3.634528000	4.193519000	-4.814263000
C	3.901752000	2.476264000	-3.563779000
H	3.710754000	3.026427000	-2.653187000
C	9.137145000	5.391565000	6.057911000
C	8.667128000	6.239141000	7.065904000
H	7.888989000	5.885156000	7.728433000
C	9.172481000	7.523797000	7.212482000
H	8.786497000	8.166961000	7.992013000
C	10.168470000	7.981483000	6.357151000
H	10.569607000	8.979395000	6.474568000
C	10.648291000	7.148000000	5.352045000
H	11.427639000	7.496322000	4.687394000
C	10.137260000	5.864295000	5.202337000
H	10.526562000	5.214001000	4.430764000

Table S11. Cartesian coordinates (XYZ) of DFT optimized geometry of **11** (def2-TZVPP/B3LYP).

C	0.846540000	2.009558000	8.424204000
H	-0.044787000	2.551075000	8.708773000
C	1.376150000	2.236234000	7.159595000
C	2.585625000	1.603017000	6.802139000
C	3.194060000	1.788992000	5.526264000
H	2.699247000	2.417602000	4.801644000
C	4.367824000	1.189421000	5.205610000
H	4.779444000	1.369129000	4.224799000
C	5.042803000	0.310713000	6.105228000
C	4.433579000	0.049585000	7.365873000
C	5.035724000	-0.875231000	8.266533000
C	4.375873000	-1.125386000	9.506381000
H	4.780354000	-1.857534000	10.186720000
C	3.237969000	-0.477189000	9.860442000
H	2.766358000	-0.708020000	10.803718000
C	2.626472000	0.486397000	9.004776000
C	1.449805000	1.181168000	9.364521000
C	3.213549000	0.715022000	7.725120000
C	0.633307000	3.167439000	6.275714000
C	1.195157000	4.344844000	5.774044000
H	2.232294000	4.571959000	5.982650000
C	0.424299000	5.251712000	5.061652000
H	0.893469000	6.163537000	4.717222000
C	-0.935023000	5.033196000	4.810912000
C	-1.481058000	3.843266000	5.289236000
H	-2.521251000	3.610616000	5.118065000
C	-0.714016000	2.932846000	6.005978000
H	-1.173019000	2.022576000	6.368868000
C	-1.773040000	6.096496000	4.095409000
C	-1.199724000	6.382814000	2.694141000
H	-1.213303000	5.479864000	2.082627000
H	-0.173529000	6.749320000	2.735783000
H	-1.800938000	7.141193000	2.189963000
C	-1.753364000	7.388847000	4.935165000
H	-0.742644000	7.777303000	5.056761000
H	-2.162676000	7.204291000	5.929487000
H	-2.358222000	8.160615000	4.454898000
C	-3.236601000	5.664853000	3.931481000
H	-3.792753000	6.455283000	3.426814000
H	-3.713743000	5.485531000	4.895582000
H	-3.332320000	4.760109000	3.329543000
C	0.852865000	1.142191000	10.718229000
C	-0.523654000	0.992846000	10.890053000
H	-1.144998000	0.789383000	10.027462000

C	-1.113788000	1.108318000	12.143716000
H	-2.184097000	0.986257000	12.219761000
C	-0.354731000	1.383142000	13.280253000
C	1.028597000	1.498866000	13.107862000
H	1.658678000	1.712631000	13.960697000
C	1.621524000	1.385169000	11.861874000
H	2.688632000	1.531848000	11.764788000
C	-0.969548000	1.598183000	14.665157000
C	-0.384065000	0.580692000	15.660815000
H	-0.607849000	-0.440026000	15.346739000
H	-0.812435000	0.733189000	16.653334000
H	0.698247000	0.676553000	15.743373000
C	-0.649059000	3.030963000	15.135311000
H	0.423563000	3.194920000	15.237199000
H	-1.113409000	3.222455000	16.104750000
H	-1.032167000	3.760392000	14.420845000
C	-2.493350000	1.444232000	14.655665000
H	-2.963210000	2.160554000	13.981385000
H	-2.885878000	1.633105000	15.655781000
H	-2.798785000	0.440358000	14.356842000
C	10.449923000	-3.219506000	5.603380000
H	11.338540000	-3.764098000	5.316835000
C	9.874813000	-3.505028000	6.834401000
C	8.676924000	-2.856754000	7.196705000
C	8.043617000	-3.085466000	8.452813000
H	8.508474000	-3.765703000	9.150860000
C	6.888433000	-2.458906000	8.787906000
H	6.459263000	-2.667538000	9.755097000
C	6.246939000	-1.530734000	7.913538000
C	6.866433000	-1.253925000	6.660928000
C	6.273155000	-0.316543000	5.767693000
C	6.959487000	-0.027177000	4.550621000
H	6.567957000	0.728182000	3.887792000
C	8.112739000	-0.654522000	4.206773000
H	8.609298000	-0.387519000	3.285996000
C	8.704430000	-1.647595000	5.043110000
C	9.888693000	-2.332763000	4.689180000
C	8.085548000	-1.918990000	6.299920000
C	10.538756000	-4.544461000	7.658643000
C	9.909486000	-5.757508000	7.952429000
H	8.879625000	-5.905885000	7.654860000
C	10.603660000	-6.789741000	8.563861000
H	10.085041000	-7.722482000	8.740382000
C	11.950875000	-6.661747000	8.923104000
C	12.560637000	-5.435568000	8.661434000
H	13.593612000	-5.273147000	8.930240000
C	11.871615000	-4.400297000	8.039549000
H	12.382085000	-3.467678000	7.837115000
C	12.698479000	-7.842096000	9.549703000
C	12.029779000	-8.238340000	10.879292000
H	12.052153000	-7.403813000	11.581156000
H	10.990971000	-8.538585000	10.740350000
H	12.560152000	-9.076835000	11.334279000
C	12.664148000	-9.034828000	8.575525000
H	11.645465000	-9.352838000	8.355154000
H	13.143010000	-8.768665000	7.631840000
H	13.199785000	-9.885267000	9.002204000
C	14.169006000	-7.513018000	9.835127000
H	14.653474000	-8.380796000	10.284463000
H	14.709240000	-7.264658000	8.920770000
H	14.275585000	-6.680908000	10.532031000
C	10.542512000	-2.238789000	3.364182000
C	11.929273000	-2.113435000	3.269548000
H	12.508036000	-1.952227000	4.169760000
C	12.583509000	-2.210286000	2.047622000
H	13.659082000	-2.116882000	2.033207000
C	11.881619000	-2.436658000	0.864508000

C	10.487601000	-2.514062000	0.957453000
H	9.899160000	-2.686465000	0.066105000
C	9.830325000	-2.423162000	2.173775000
H	8.756708000	-2.549166000	2.209579000
C	12.573147000	-2.644671000	-0.485186000
C	12.109653000	-1.567066000	-1.481853000
H	12.349893000	-0.569609000	-1.110553000
H	12.608378000	-1.701685000	-2.443645000
H	11.033811000	-1.611727000	-1.651222000
C	12.211472000	-4.043230000	-1.023319000
H	11.140991000	-4.148806000	-1.198387000
H	12.725835000	-4.227777000	-1.968346000
H	12.512546000	-4.813728000	-0.312536000
C	14.099342000	-2.576263000	-0.371493000
H	14.482998000	-3.326953000	0.319669000
H	14.547494000	-2.772375000	-1.346320000
H	14.437899000	-1.595452000	-0.034564000

Table S12. Cartesian coordinates (XYZ) of DFT optimized geometry of **13** (def2-TZVPP/B3LYP).

C	-5.055419000	-0.402654000	-0.635663000
C	-3.665924000	-0.609994000	-0.620236000
C	-2.821209000	0.396573000	-0.083302000
C	-3.388192000	1.600827000	0.410000000
C	-4.779588000	1.782715000	0.339927000
C	-5.622297000	0.788567000	-0.169378000
C	-3.076321000	-1.805339000	-1.121445000
C	-1.733233000	-1.992260000	-1.082612000
C	-0.844800000	-1.012369000	-0.541552000
C	-1.404506000	0.198148000	-0.039517000
C	-0.565557000	1.212777000	0.505762000
C	-1.182004000	2.403609000	0.999902000
C	-2.524605000	2.592852000	0.955722000
C	0.561009000	-1.209310000	-0.497958000
C	1.399959000	-0.194685000	0.047329000
C	0.840252000	1.015840000	0.549346000
C	1.177460000	-2.400113000	-0.992161000
C	2.520063000	-2.589351000	-0.948000000
C	3.383644000	-1.597372000	-0.402180000
C	2.816663000	-0.393108000	0.091109000
C	3.661383000	0.613493000	0.627992000
C	3.071774000	1.808832000	1.129205000
C	1.728685000	1.995745000	1.090377000
C	4.775054000	-1.779211000	-0.332143000
C	5.617735000	-0.785092000	0.177195000
C	5.050883000	0.406157000	0.643432000
C	-7.090455000	0.993146000	-0.213279000
C	7.085824000	-0.990044000	0.221594000
C	7.766978000	-0.996230000	1.439649000
C	9.140631000	-1.194567000	1.482837000
C	9.857205000	-1.376335000	0.305041000
C	9.189053000	-1.365140000	-0.914451000
C	7.813422000	-1.180288000	-0.954031000
C	-7.817834000	1.182290000	0.962661000
C	-9.193582000	1.366362000	0.923577000
C	-9.862080000	1.377925000	-0.295721000
C	-9.145734000	1.197242000	-1.473824000
C	-7.771966000	0.999592000	-1.431133000
O	-5.860895000	-1.384075000	-1.123442000
Si	-6.680661000	-2.563213000	-0.250997000
C	-6.076811000	-4.216910000	-0.882027000
C	-8.506878000	-2.384514000	-0.583538000
C	-6.280454000	-2.350594000	1.561002000
O	-5.312679000	2.951448000	0.786864000
Si	-5.723649000	4.303043000	-0.122891000
C	-7.546198000	4.632741000	0.094029000

C	-5.285693000	3.975674000	-1.908942000
C	-4.730918000	5.733012000	0.560550000
O	5.856342000	1.387458000	1.131480000
Si	6.676240000	2.566758000	0.259388000
C	6.072504000	4.220329000	0.890853000
C	6.276095000	2.354654000	-1.552686000
C	8.502433000	2.387828000	0.591950000
O	5.308370000	-2.947755000	-0.779280000
Si	5.720960000	-4.298884000	0.130406000
C	7.543745000	-4.626728000	-0.087357000
C	4.729324000	-5.729915000	-0.552407000
C	5.283535000	-3.971719000	1.916624000
H	-3.718708000	-2.558004000	-1.551722000
H	-1.332265000	-2.910783000	-1.480615000
H	-0.569140000	3.180336000	1.428797000
H	-2.960520000	3.496728000	1.352349000
H	0.564600000	-3.176815000	-1.421107000
H	2.955976000	-3.493177000	-1.344715000
H	3.714159000	2.561511000	1.559461000
H	1.327716000	2.914284000	1.488344000
H	7.212833000	-0.837059000	2.354245000
H	9.652914000	-1.200219000	2.435692000
H	10.928053000	-1.525575000	0.337293000
H	9.738711000	-1.507467000	-1.835331000
H	7.291811000	-1.189444000	-1.900990000
H	-7.295974000	1.191118000	1.909484000
H	-9.743062000	1.507828000	1.844696000
H	-10.933020000	1.526591000	-0.327587000
H	-9.658289000	1.203146000	-2.426530000
H	-7.218006000	0.841167000	-2.345969000
H	-5.028530000	-4.383172000	-0.629743000
H	-6.658360000	-5.032336000	-0.446375000
H	-6.177652000	-4.279791000	-1.967343000
H	-8.900252000	-1.456594000	-0.169912000
H	-9.056760000	-3.217346000	-0.139082000
H	-8.709531000	-2.385396000	-1.655951000
H	-6.737889000	-3.150380000	2.147169000
H	-5.203268000	-2.382035000	1.733114000
H	-6.655150000	-1.400180000	1.941711000
H	-7.807557000	4.697355000	1.151683000
H	-7.818811000	5.580562000	-0.375905000
H	-8.151191000	3.844259000	-0.352232000
H	-4.231838000	3.712931000	-2.015240000
H	-5.472979000	4.864774000	-2.514705000
H	-5.879790000	3.159240000	-2.320200000
H	-4.878002000	5.829073000	1.638126000
H	-3.663576000	5.603955000	0.375272000
H	-5.038739000	6.673288000	0.097911000
H	6.173320000	4.282883000	1.976190000
H	6.654148000	5.035823000	0.455455000
H	5.024248000	4.386767000	0.638589000
H	5.198921000	2.386220000	-1.724846000
H	6.650737000	1.404306000	-1.933611000
H	6.733623000	3.154547000	-2.138633000
H	9.052396000	3.220727000	0.147719000
H	8.705059000	2.388424000	1.664367000
H	8.895746000	1.459987000	0.178090000
H	8.148131000	-3.837412000	0.358290000
H	7.817636000	-5.574081000	0.382775000
H	7.804604000	-4.691443000	-1.145129000
H	3.661950000	-5.601945000	-0.366589000
H	5.038353000	-6.669833000	-0.089844000
H	4.875954000	-5.825920000	-1.630051000
H	5.877023000	-3.154641000	2.327489000
H	4.229473000	-3.709999000	2.023387000
H	5.471984000	-4.860552000	2.522417000

Table S13. Cartesian coordinates (XYZ) of DFT optimized geometry of **14** (def2-TZVPP/B3LYP).

C	-4.967377000	-0.682564000	0.389442000
C	-3.583534000	-0.861200000	0.373942000
C	-2.760124000	0.247780000	0.043714000
C	-3.359207000	1.494677000	-0.273355000
C	-4.753428000	1.616898000	-0.190770000
C	-5.581691000	0.540228000	0.128459000
C	-2.970178000	-2.114129000	0.664619000
C	-1.621265000	-2.246827000	0.649045000
C	-0.752501000	-1.155883000	0.335726000
C	-1.338601000	0.102697000	0.018697000
C	-0.522116000	1.218368000	-0.323171000
C	-1.168802000	2.445161000	-0.665802000
C	-2.517986000	2.582996000	-0.649290000
C	0.660029000	-1.294262000	0.325625000
C	1.476513000	-0.178610000	-0.016305000
C	0.890410000	1.079960000	-0.333378000
C	1.306717000	-2.521003000	0.668443000
C	2.655902000	-2.658833000	0.651968000
C	3.497122000	-1.570573000	0.275860000
C	2.898037000	-0.323703000	-0.041332000
C	3.721442000	0.785246000	-0.371679000
C	3.108083000	2.038145000	-0.662480000
C	1.759172000	2.170860000	-0.646858000
C	4.891345000	-1.692805000	0.193271000
C	5.719596000	-0.616152000	-0.126053000
C	5.105284000	0.606608000	-0.387164000
C	-7.052624000	0.691225000	0.227037000
C	7.190539000	-0.767105000	-0.224542000
C	7.850014000	-0.438455000	-1.411443000
C	9.225608000	-0.590377000	-1.522555000
C	9.962349000	-1.067105000	-0.444303000
C	9.314501000	-1.390405000	0.742896000
C	7.938312000	-1.243386000	0.852520000
C	-7.712044000	0.362554000	1.413965000
C	-9.087612000	0.514610000	1.525195000
C	-9.824391000	0.991495000	0.447036000
C	-9.176602000	1.314818000	-0.740189000
C	-7.800434000	1.167674000	-0.849928000
O	-5.764837000	-1.772874000	0.716751000
C	-7.468805000	-3.566467000	1.702558000
C	-7.434446000	-3.433062000	0.178342000
C	-6.446693000	-2.378994000	-0.309310000
C	-8.819285000	-3.006666000	-0.344238000
C	-7.025104000	-4.770029000	-0.463621000
O	-6.268637000	-2.088717000	-1.457741000
O	-5.339201000	2.819169000	-0.518633000
C	-4.530509000	2.966477000	2.438799000
C	-5.292499000	4.066491000	1.686142000
C	-5.206352000	4.023378000	0.152757000
C	-4.758756000	5.431715000	2.141341000
C	-6.799410000	3.967646000	2.009981000
O	-5.189744000	5.009057000	-0.524285000
O	5.902754000	1.696864000	-0.714617000
C	7.163707000	4.693876000	0.464956000
C	7.572889000	3.356641000	-0.176563000
C	6.584875000	2.302918000	0.311307000
C	8.957591000	2.930158000	0.346292000
C	7.607444000	3.489593000	-1.700816000
O	6.406988000	2.012688000	1.459775000
O	5.477166000	-2.895006000	0.521301000
C	6.937448000	-4.044048000	-2.006725000
C	5.430493000	-4.142928000	-1.683108000
C	5.344057000	-4.099390000	-0.149754000
C	4.896941000	-5.508319000	-2.138021000
C	4.668532000	-3.043181000	-2.436191000

O	5.327241000	-5.084874000	0.527561000
H	-3.598753000	-2.957978000	0.905924000
H	-1.199541000	-3.212886000	0.875784000
H	-0.574749000	3.293014000	-0.967074000
H	-2.963229000	3.520516000	-0.945860000
H	0.712663000	-3.368794000	0.969886000
H	3.101145000	-3.596295000	0.948718000
H	3.736657000	2.881956000	-0.903919000
H	1.337451000	3.136890000	-0.873725000
H	7.277929000	-0.070545000	-2.251644000
H	9.720515000	-0.338746000	-2.451033000
H	11.034287000	-1.185175000	-0.528133000
H	9.881730000	-1.757907000	1.587315000
H	7.437641000	-1.494281000	1.776090000
H	-7.139933000	-0.005464000	2.254100000
H	-9.582472000	0.262963000	2.453693000
H	-10.896310000	1.109674000	0.530960000
H	-9.743857000	1.682450000	-1.584533000
H	-7.299810000	1.418612000	-1.773511000
H	-6.498221000	-3.854239000	2.104702000
H	-7.768607000	-2.633199000	2.176335000
H	-8.192096000	-4.335224000	1.978045000
H	-9.559376000	-3.756383000	-0.061431000
H	-9.120955000	-2.048808000	0.079517000
H	-8.809686000	-2.913981000	-1.428550000
H	-7.754631000	-5.537998000	-0.203975000
H	-6.047723000	-5.097117000	-0.104872000
H	-6.981409000	-4.680919000	-1.547623000
H	-4.945824000	1.976546000	2.277030000
H	-3.475228000	2.946231000	2.170773000
H	-4.598866000	3.181008000	3.506162000
H	-4.885469000	5.524849000	3.220398000
H	-3.697209000	5.532305000	1.912778000
H	-5.286449000	6.246649000	1.652812000
H	-6.935740000	4.035438000	3.090242000
H	-7.222935000	3.024738000	1.671372000
H	-7.349843000	4.783681000	1.542348000
H	6.186421000	5.021027000	0.106006000
H	7.119890000	4.605100000	1.548982000
H	7.893395000	5.461640000	0.205155000
H	9.697848000	3.679658000	0.063345000
H	8.947859000	2.837786000	1.430628000
H	9.259131000	1.972118000	-0.077153000
H	8.330918000	4.258127000	-1.976445000
H	6.636964000	3.777431000	-2.103159000
H	7.907131000	2.556127000	-2.174279000
H	7.360823000	-3.100979000	-1.668384000
H	7.487891000	-4.859859000	-1.538711000
H	7.073965000	-4.112220000	-3.086939000
H	5.023785000	-5.601715000	-3.217040000
H	5.424664000	-6.323078000	-1.649229000
H	3.835381000	-5.608963000	-1.909546000
H	4.737125000	-3.257967000	-3.503487000
H	5.083703000	-2.053165000	-2.274582000
H	3.613196000	-3.022990000	-2.168373000

NMR Spectra

*1,3,8,10-tetrakis((trimethylsilyl)oxydibenzo[cd,lm]-perylene (**2**)*

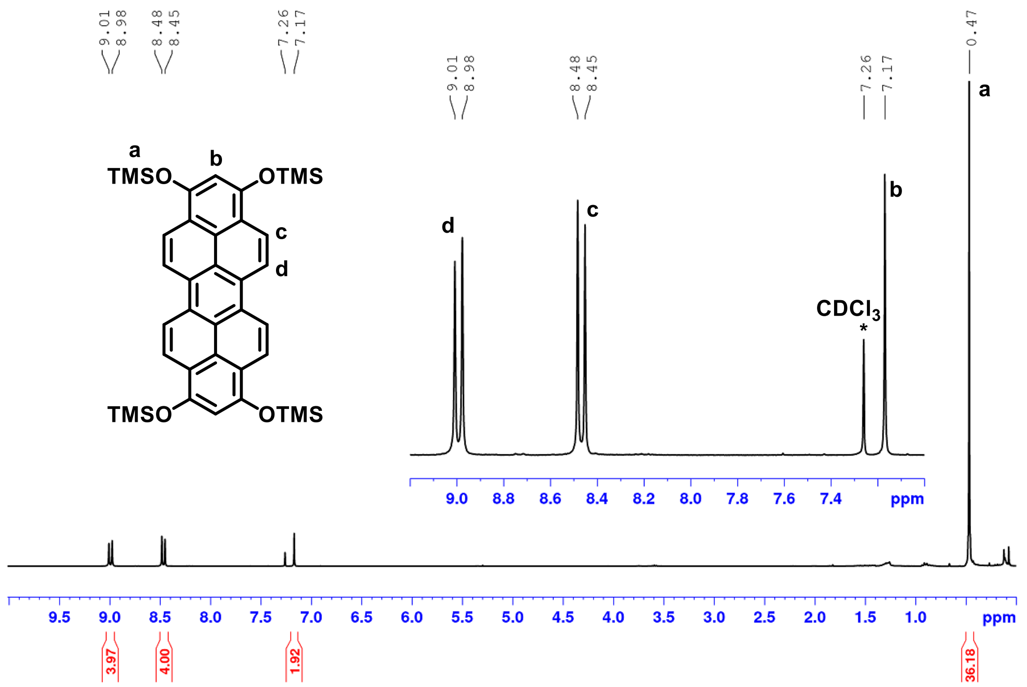

Figure S24. ^{1}H-NMR (300.1 MHz, 298K, $CDCl_3$*) of **2**.

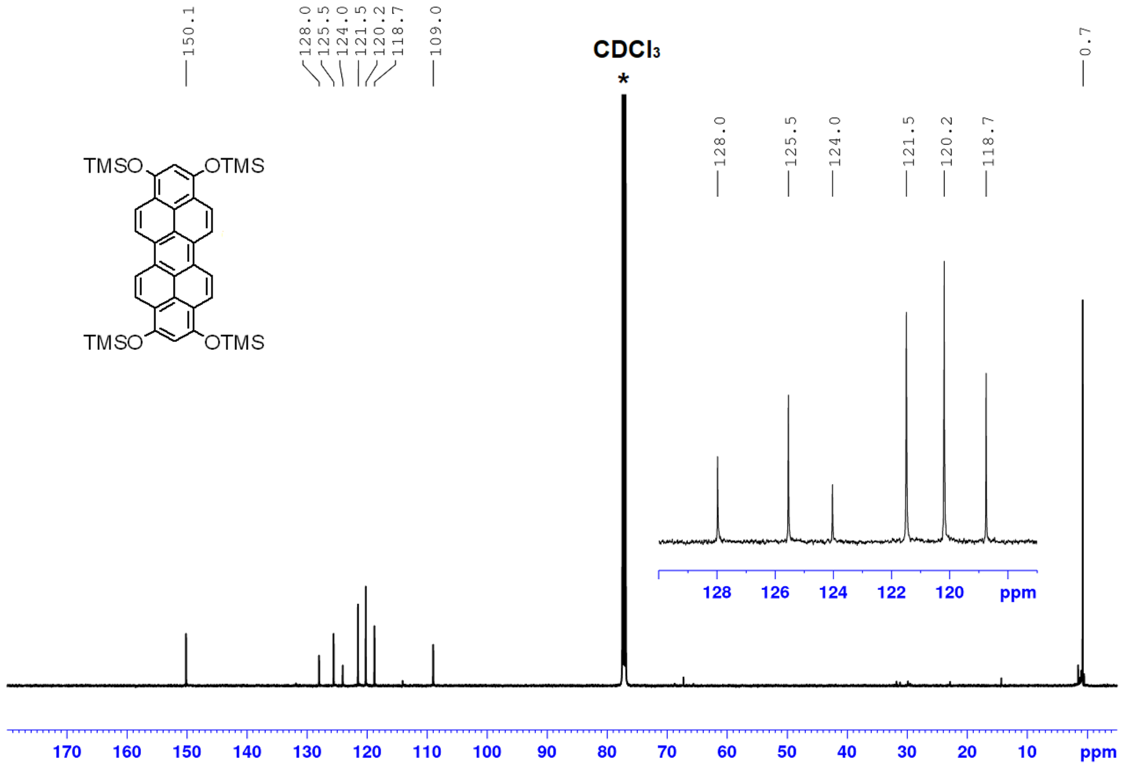

Figure S25. ^{13}C-NMR (125.8 MHz, 298K, $CDCl_3$*) of **2**.

*1,3,8,10-tetrakis((triisopropylsilyl)oxy)dibenzo[cd,lm]-perylene (**3**)*

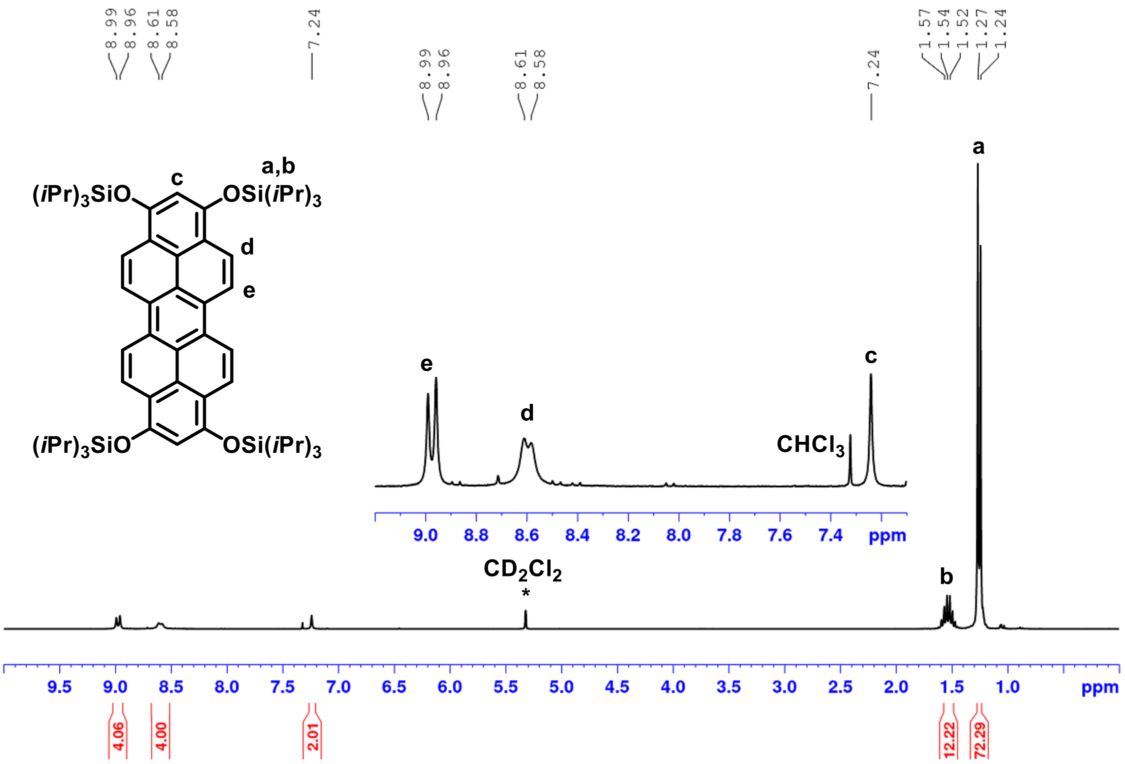

Figure S26. ^{1}H-NMR (300.1 MHz, 298K, CD_2Cl_2*) of **3**.

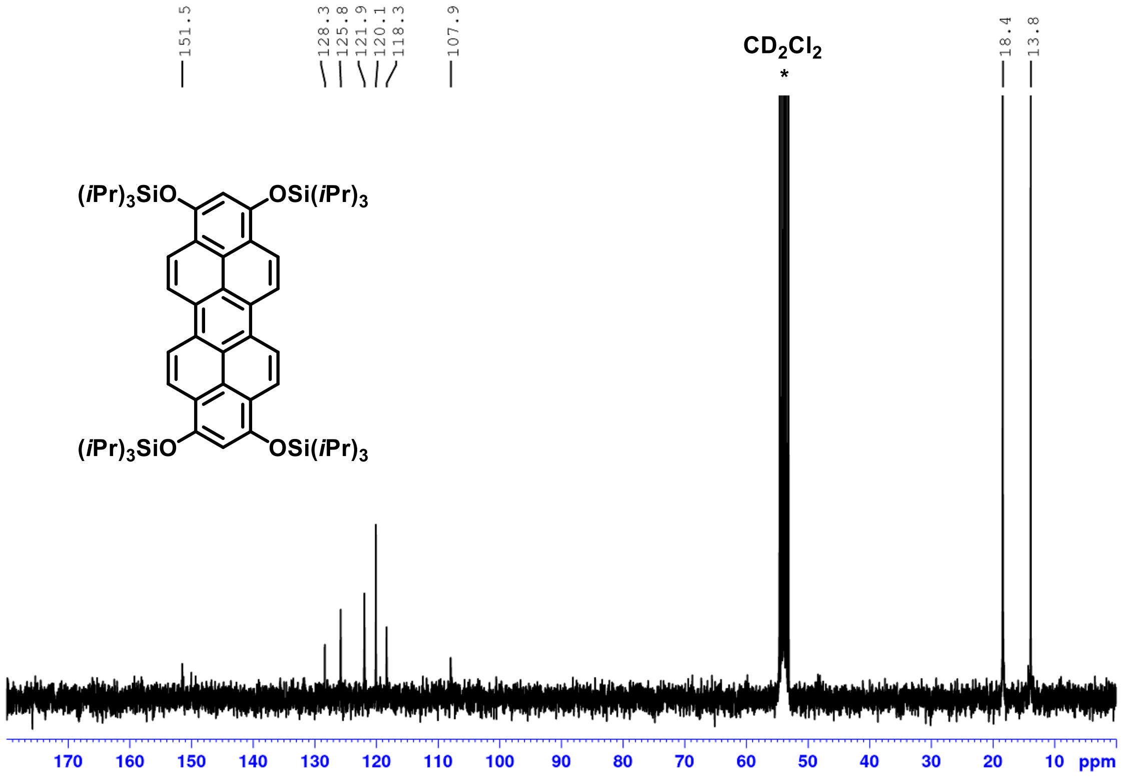

Figure S27. ^{13}C-NMR (75.5 MHz, 298K, $CDCl_3$*) of **4**.

*Dibenzo[cd,lm]perylene-1,3,8,10-tetrayltetrakis(2,2-dimethyl-propanoate) (**4**)*

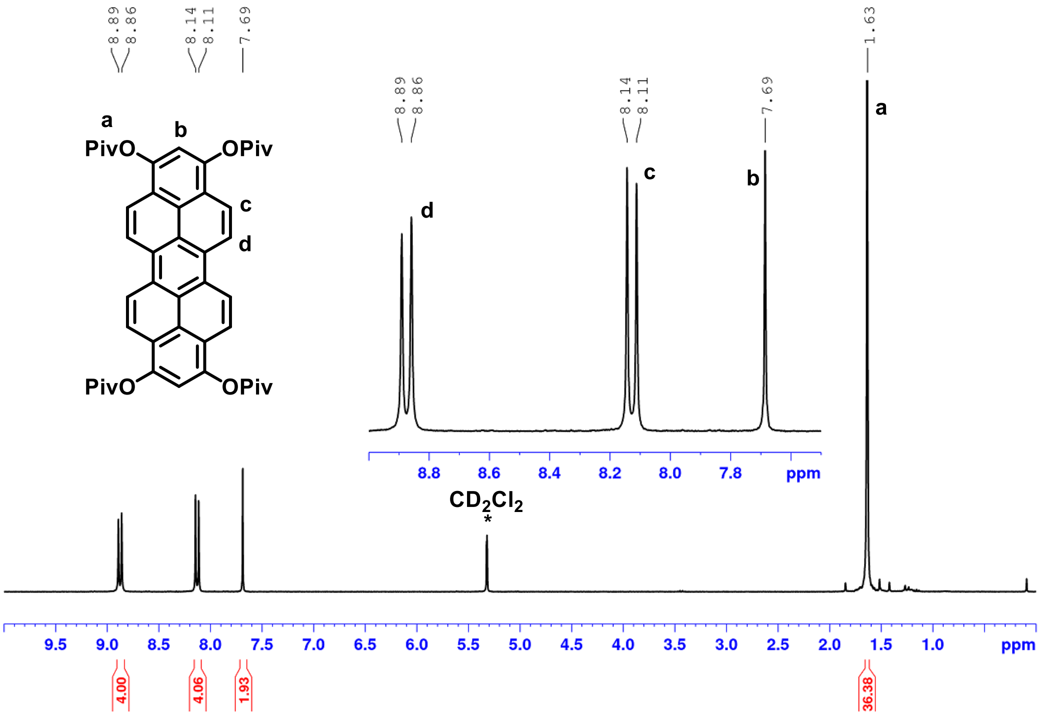

Figure S28. ^{1}H-NMR (300.1 MHz, 298K, CD_2Cl_2*) of **4**.

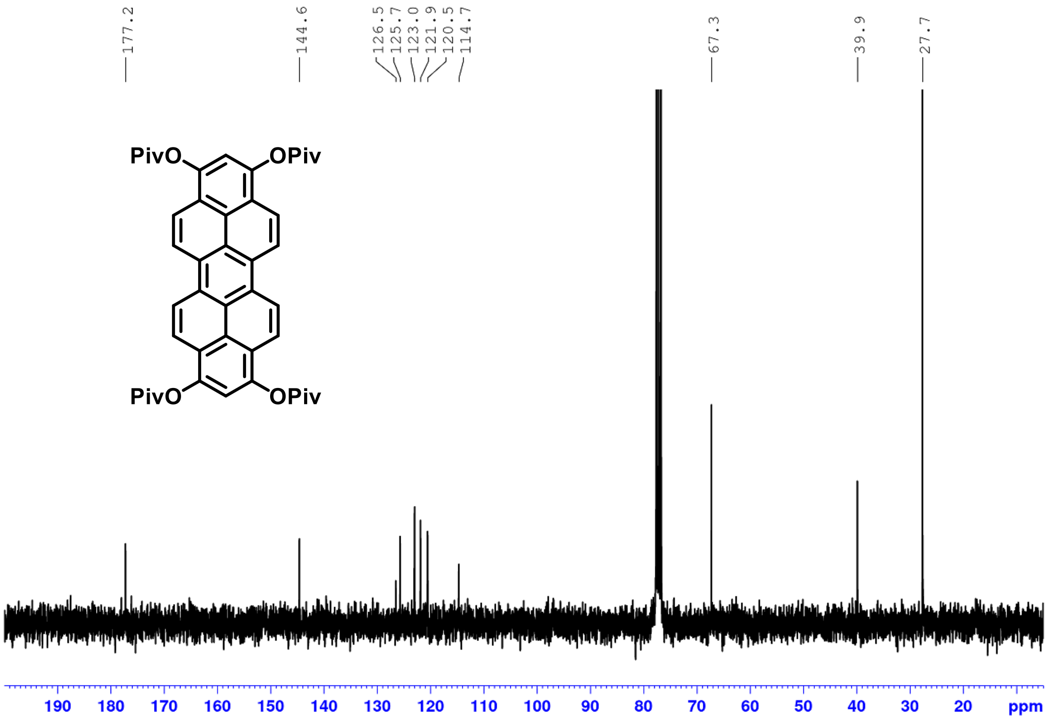

Figure S29. ^{13}C-NMR (75.5 MHz, 298K, $CDCl_3$*) of **4**.

*Dibenzo[cd,lm]perylene-1,3,8,10-tetrayl tetrakis-(trifluoromethanesulfonate) (**5**)*

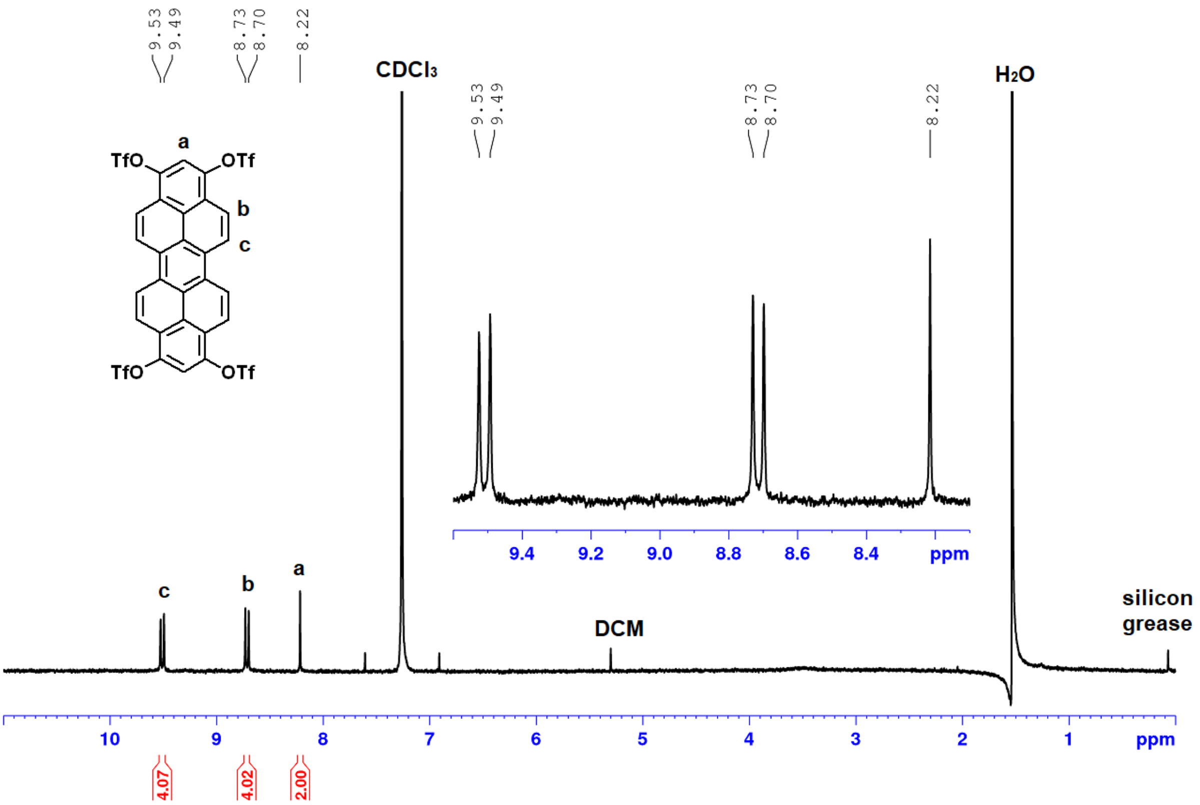

Figure S30. ^{1}H-NMR (300.1 MHz, 298K, $CDCl_3$*) of **5**.

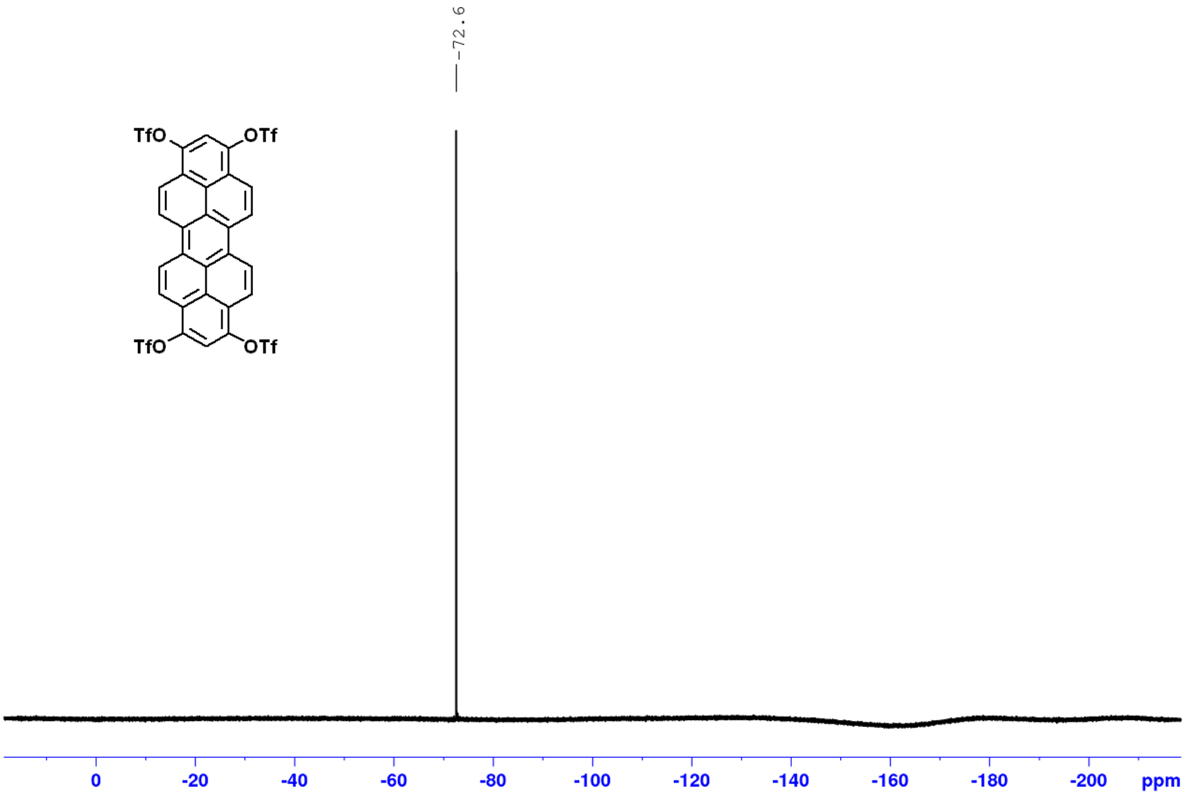

Figure S31. ^{19}F-NMR (235.3 MHz, 298K, $CDCl_3$*) of **5**.

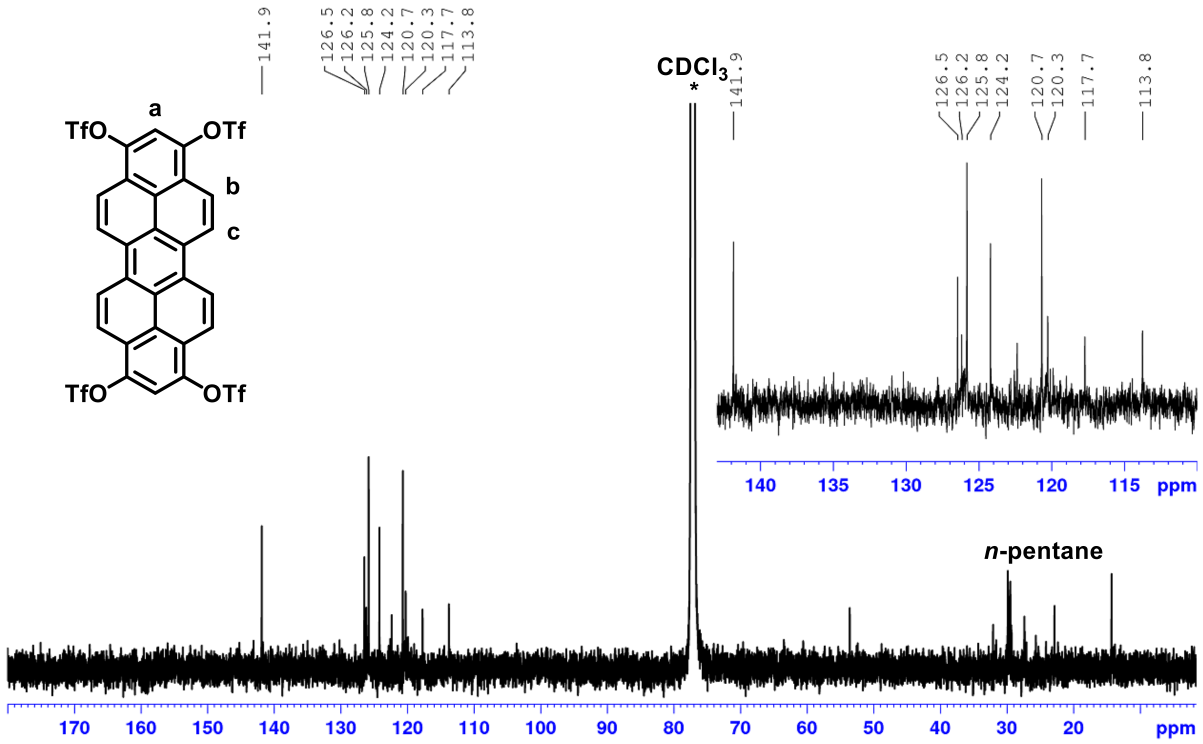

Figure S32. ^{13}C-NMR (125.8 MHz, 298K, $CDCl_3$*) of **5** with residual *n*-pentane.

*Dibenzo[cd,lm]perylene-1,3,8,10-tetrayl tetrakis-(trifluoromethanesulfonate) (**6**)*

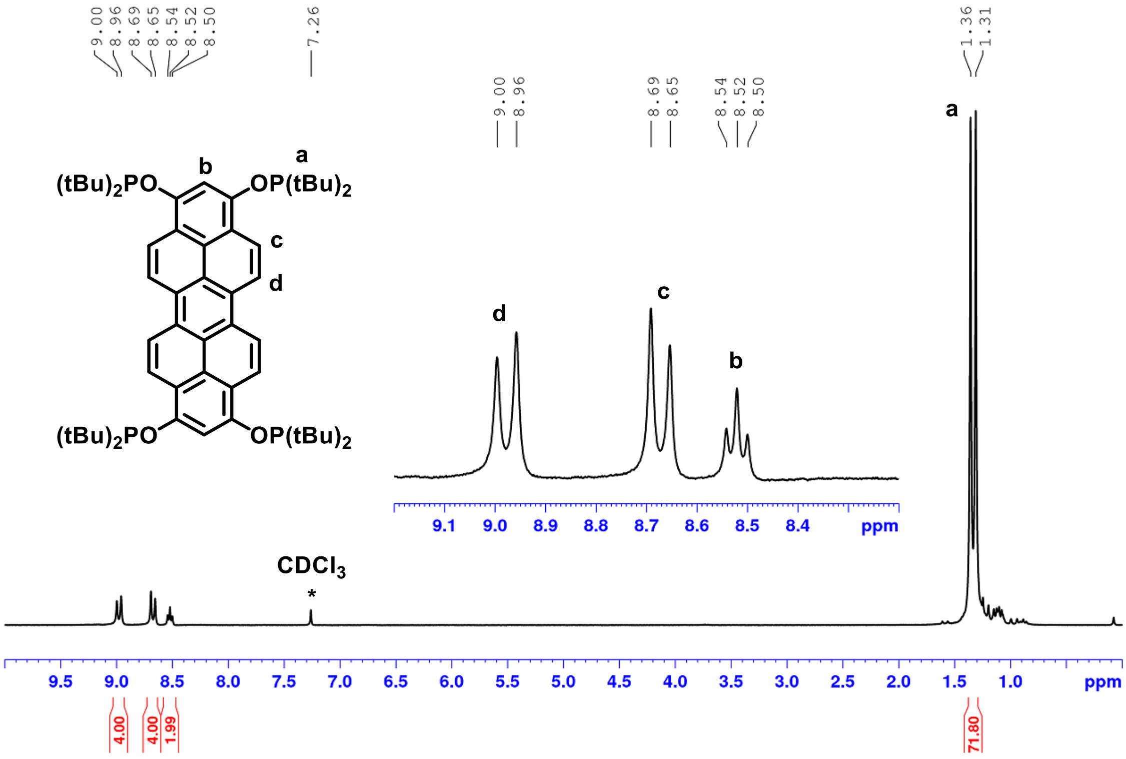

Figure S33. ^{1}H-NMR (300.1 MHz, 298K, $CDCl_3$*) of **6**.

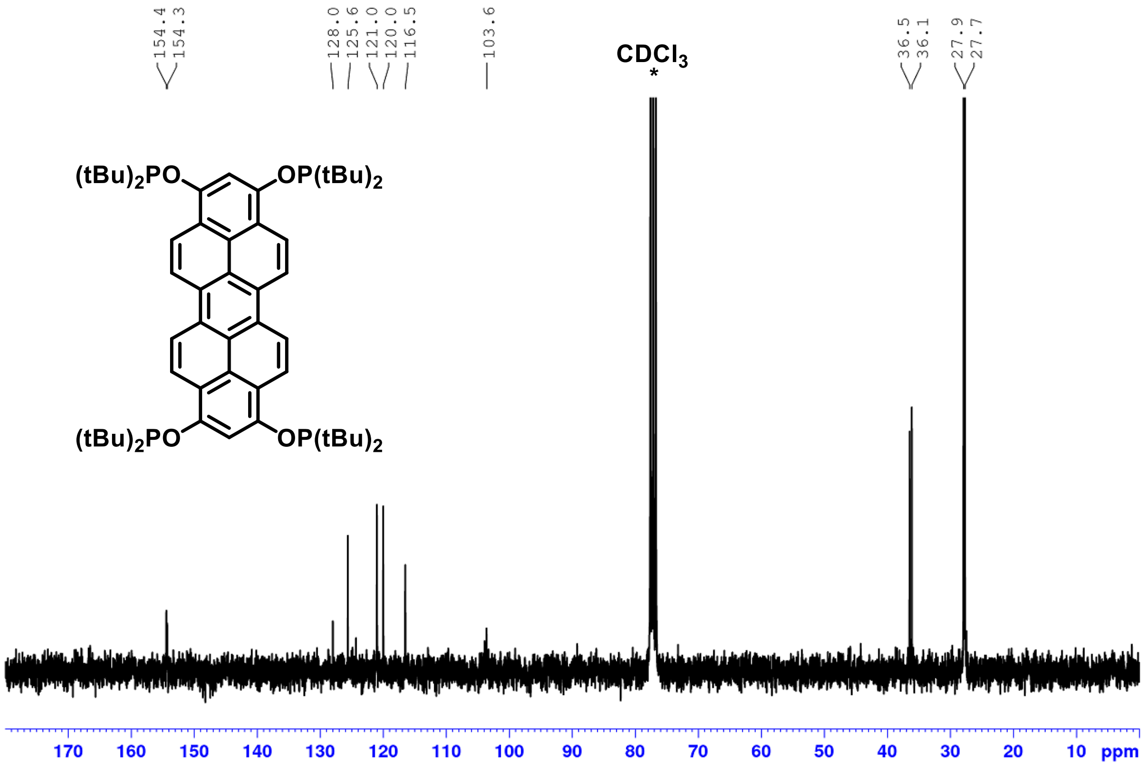

Figure S34. ^{13}C-NMR (75.5 MHz, 298K, $CDCl_3$*) of **6**.

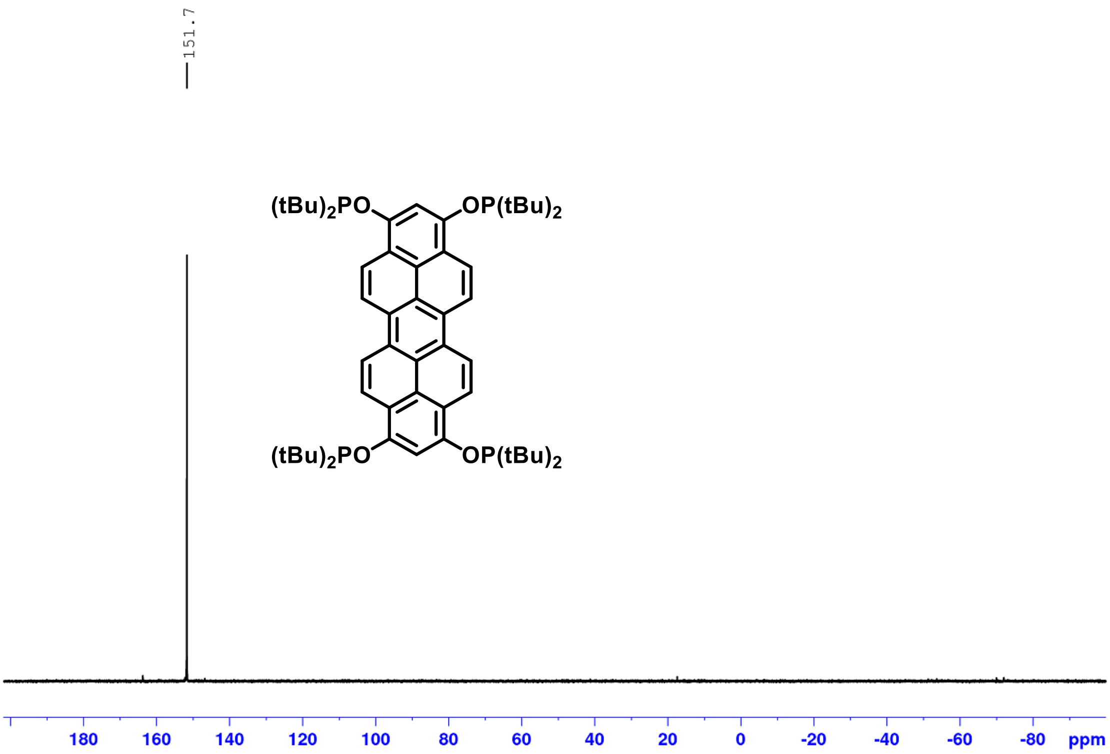

Figure S35. ^{31}P-NMR (101 MHz, 298K, $CDCl_3$*) of **6**.

*1,3,8,10-tetrakis((trimethylsilyl)ethynyl)dibenzo[cd,lm]-perylene (**7**)*

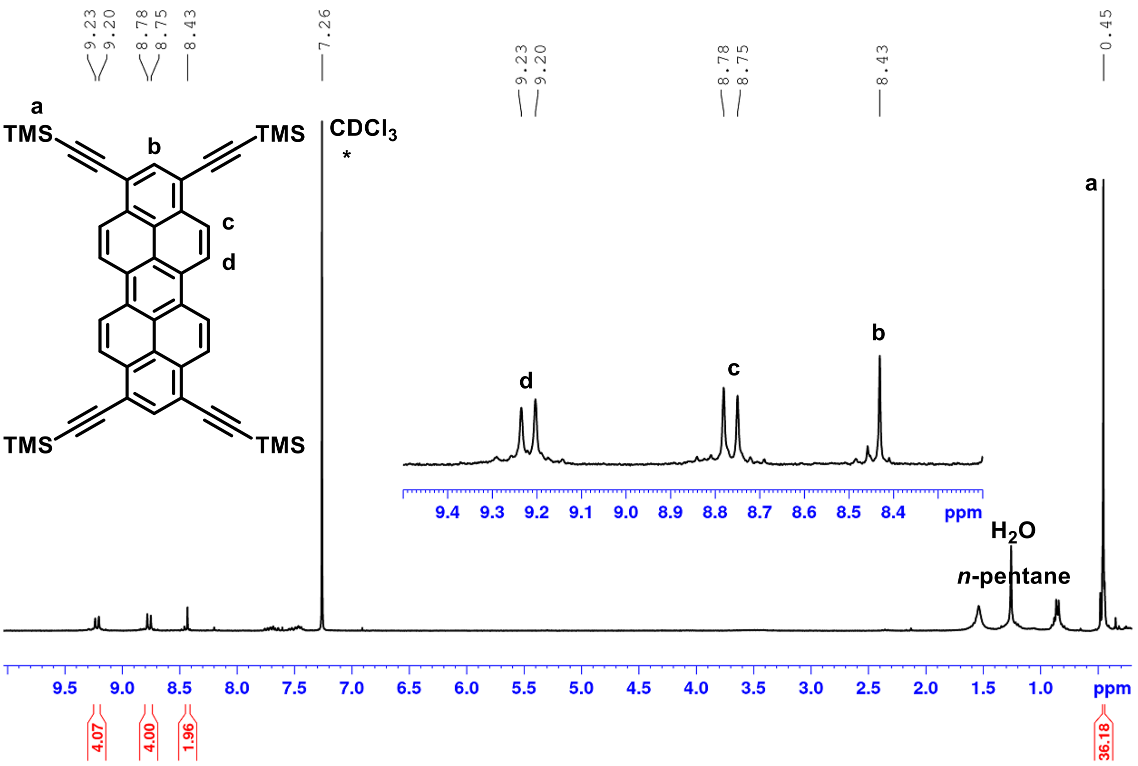

Figure S36. ^{1}H-NMR (300.1 MHz, 298K, $CDCl_3$*) of **7** with residual *n*-pentane.

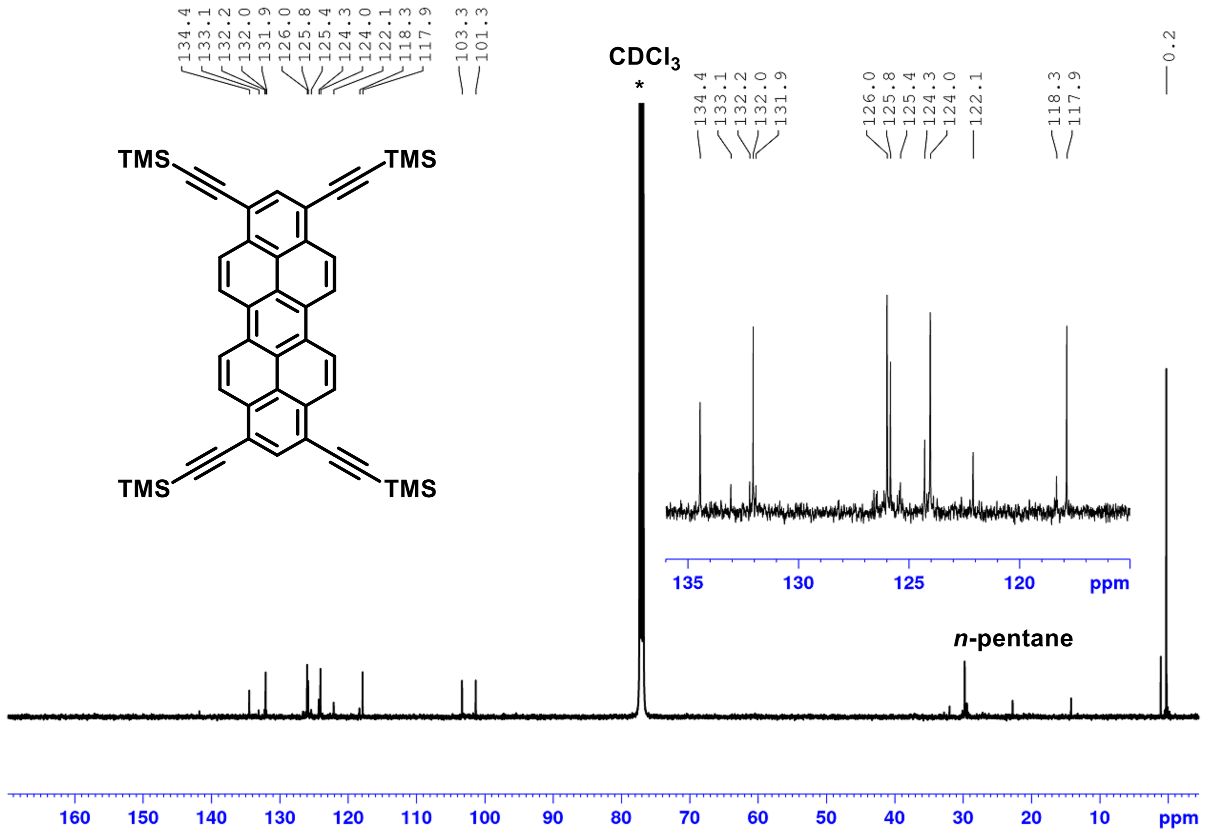

Figure S37. ^{13}C-NMR (125.8 MHz, 298K, $CDCl_3$*) of **7** with residual *n*-pentane.

*1,3,8,10-tetrakis(3,5-bis(trifluoromethyl)phenyl)dibenzo-[cd,lm]perylene (**8**)*

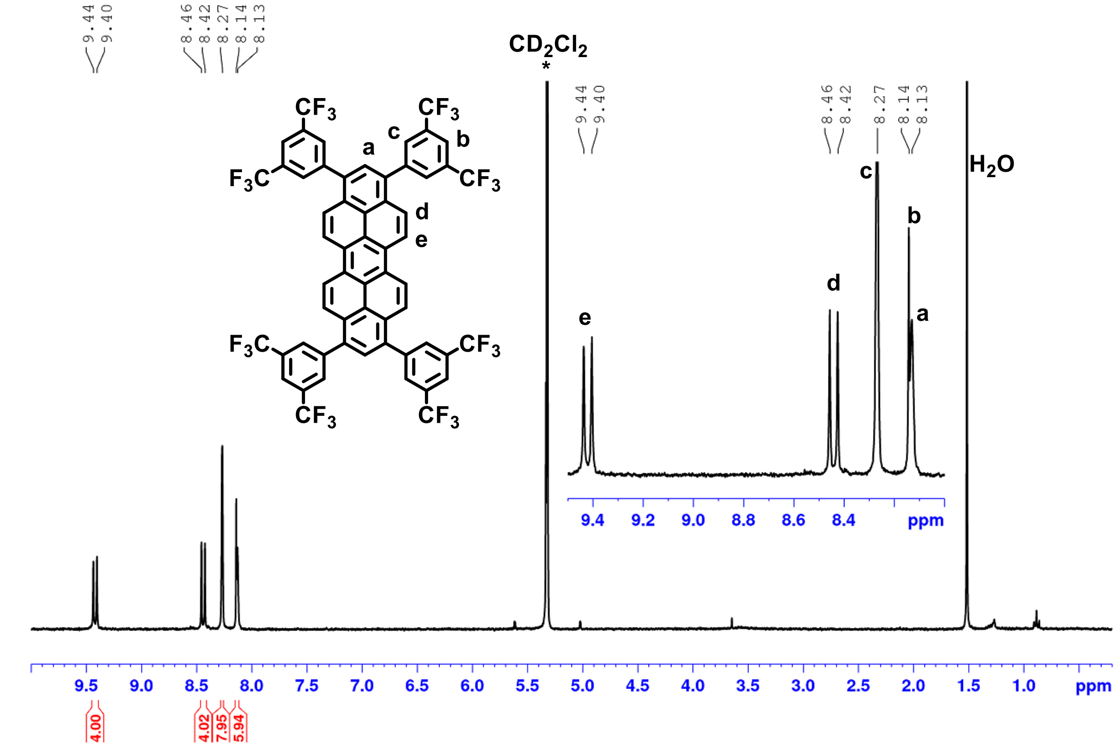

Figure S38. ^{1}H-NMR (300.1 MHz, 298K, CD_2Cl_2*) of **8**.

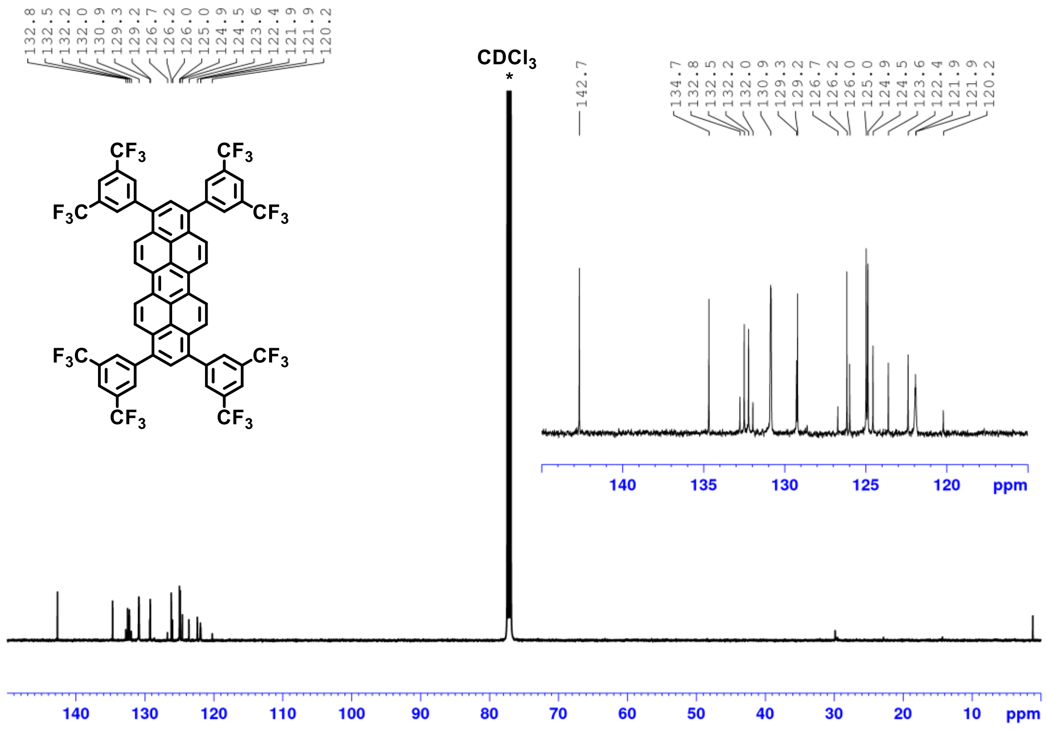

Figure S39. ^{13}C-NMR (125.8 MHz, 298K, $CDCl_3$*) of **8**.

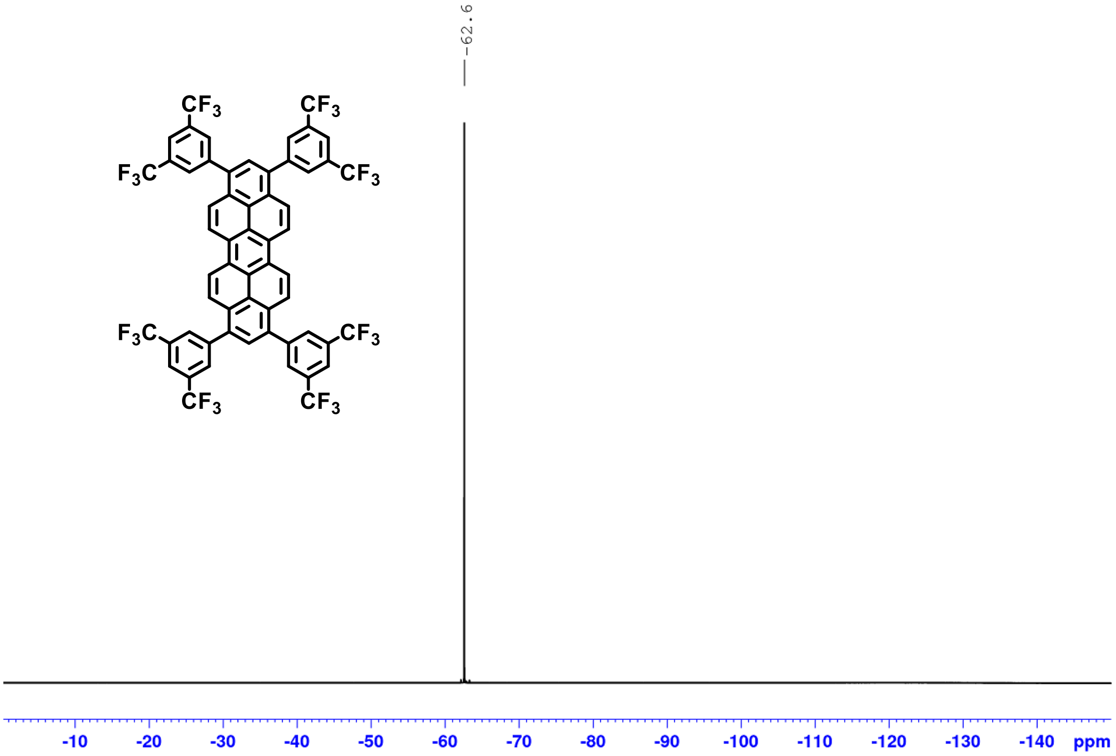

Figure S40. ^{19}F-NMR (235.3 MHz, 298K, $CDCl_3$*) of **8**.

*1,3,8,10-tetra(thiophen-2-yl)dibenzo[cd,lm]perylene (**9**)*

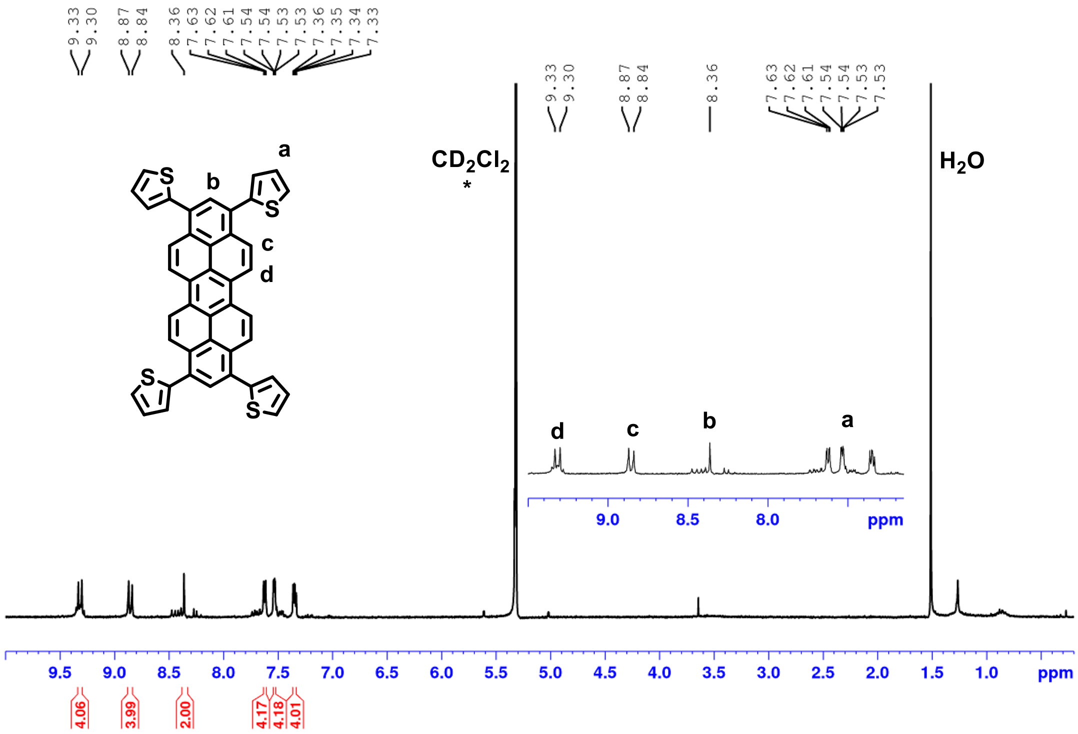

Figure S41. ^{1}H-NMR (300.1 MHz, 298K, CD_2Cl_2*) of **9**.

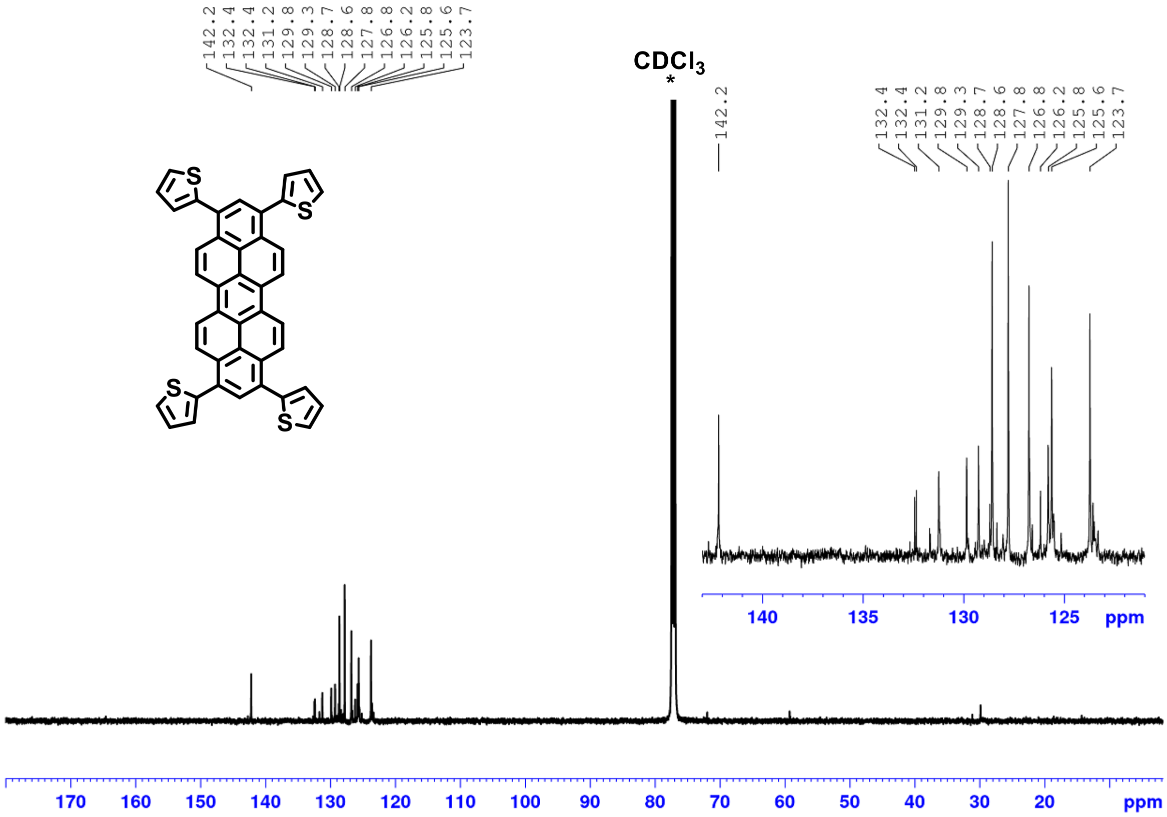

Figure S42. ^{13}C-NMR (125.8 MHz, 298K, $CDCl_3$*) of **9**.

*1,3,8,10-tetraphenyldibenzo[cd,lm]perylene (**10**)*

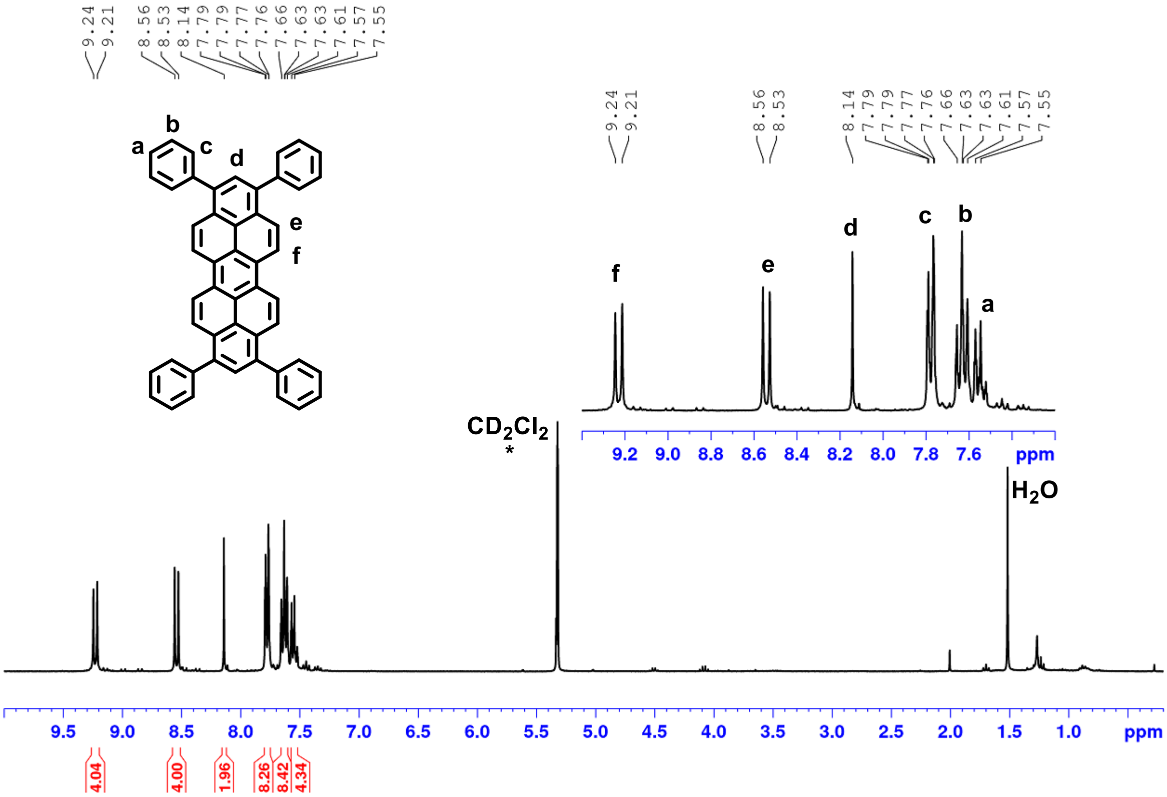

Figure S43. ^{1}H-NMR (300.1 MHz, 298K, CD_2Cl_2*) of **10**.

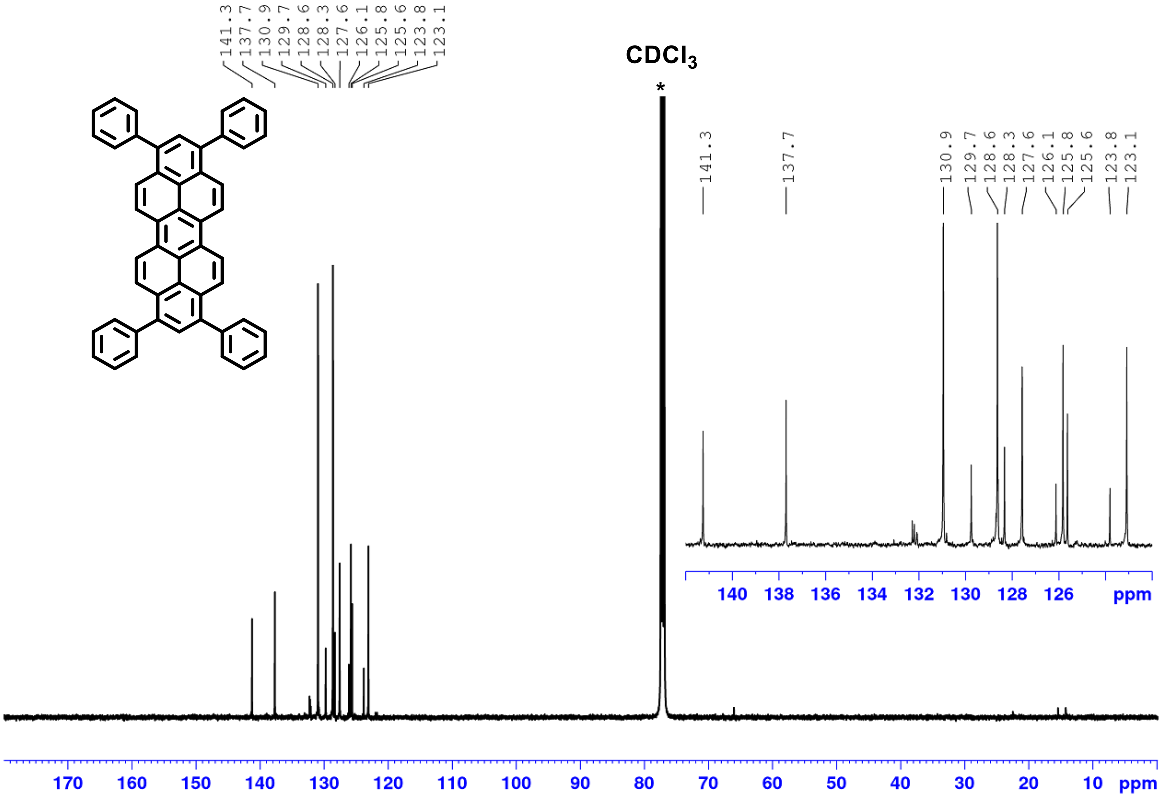

Figure S44. ^{13}C-NMR (125.8 MHz, 298K, $CDCl_3$*) of **10**.

*1,3,8,10-tetraphenyldibenzo[cd,lm]perylene (**11**)*

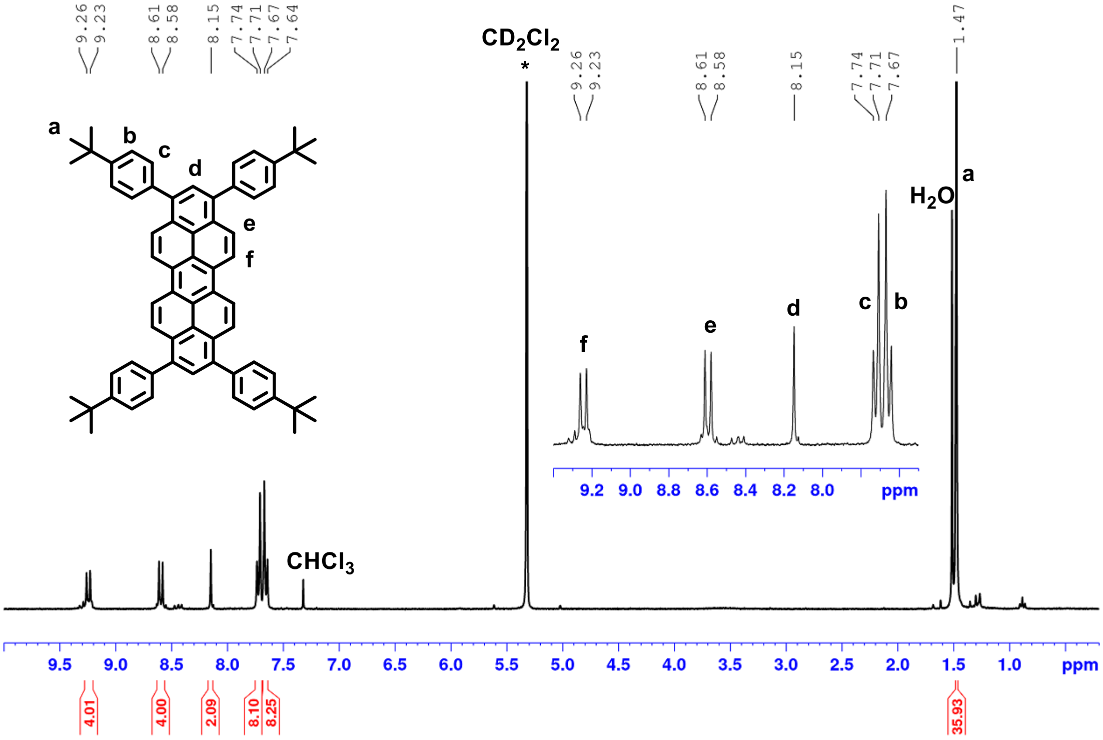

Figure S45. ^{1}H-NMR (300.1 MHz, 298K, CD_2Cl_2*) of **11**.

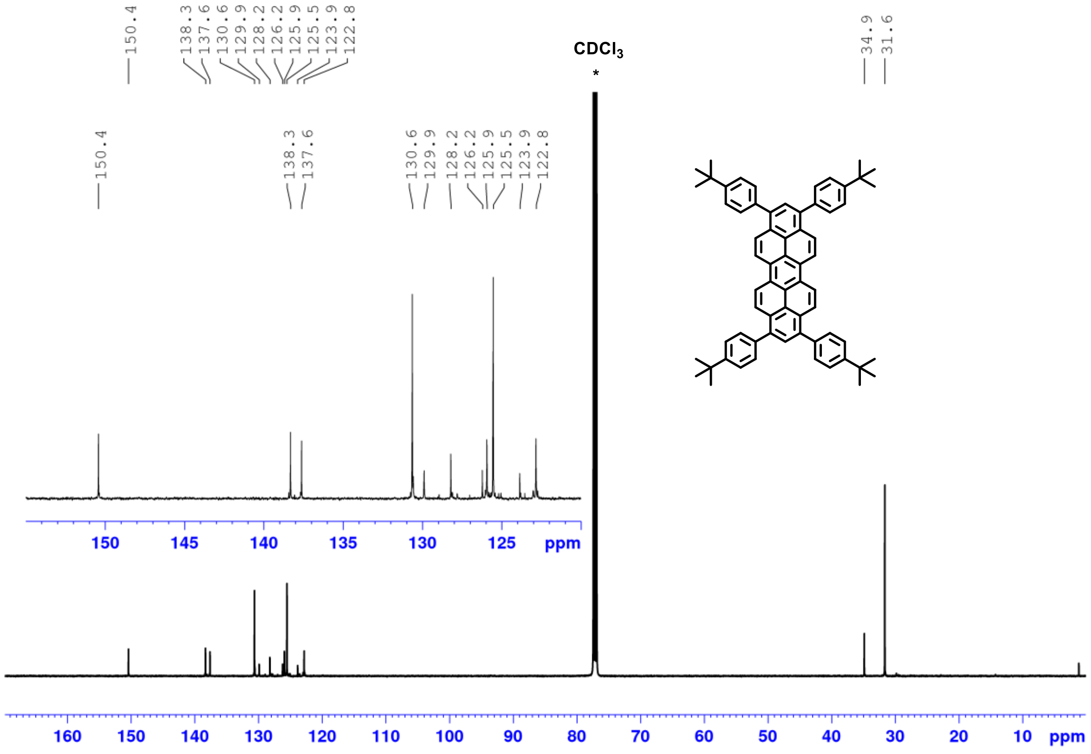

Figure S46. ^{13}C-NMR (125.8 MHz, 298K, $CDCl_3$*) of **11**.

((2,9-diphenyldibenzo[cd,lm]perylene-1,3,8,10-tetrayl)tetrakis(oxy))tetrakis(trimethylsilane) ***(13)***

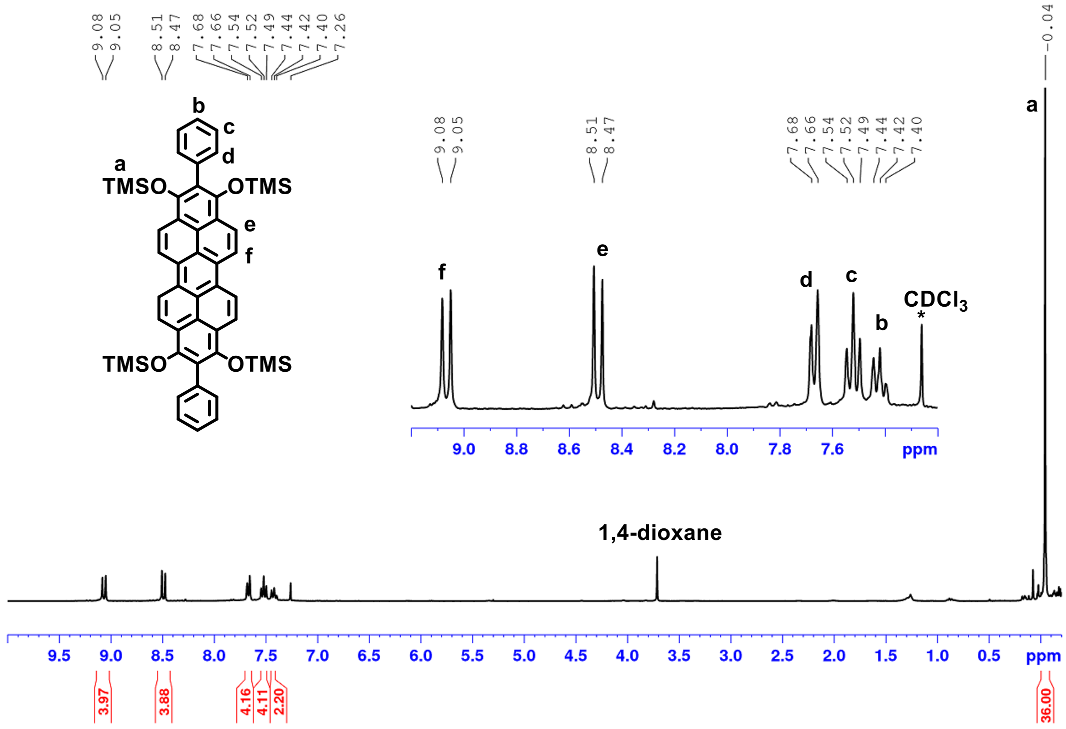

Figure S47. ^{1}H-NMR (300.1 MHz, 298K, $CDCl_3$*) of **13**.

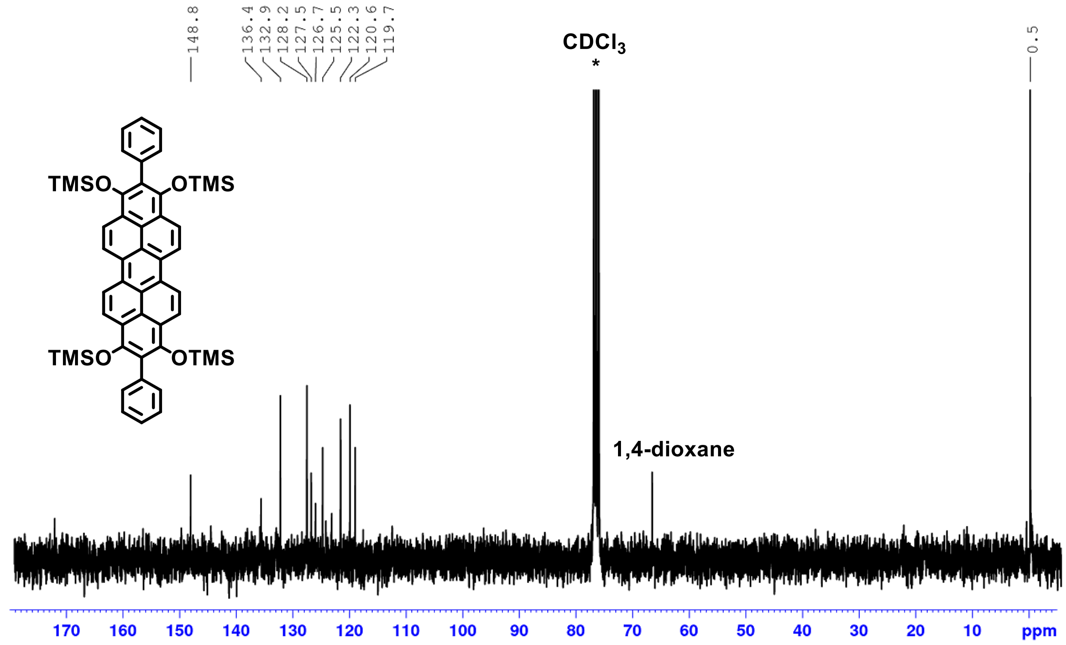

Figure S48. ^{13}C-NMR (75.5 MHz, 298K, $CDCl_3$*) of **13**.

2,9-diphenyldibenzo[cd,lm]perylene-1,3,8,10-tetrayl tetrakis(2,2-dimethylpropanoate) (**14**)

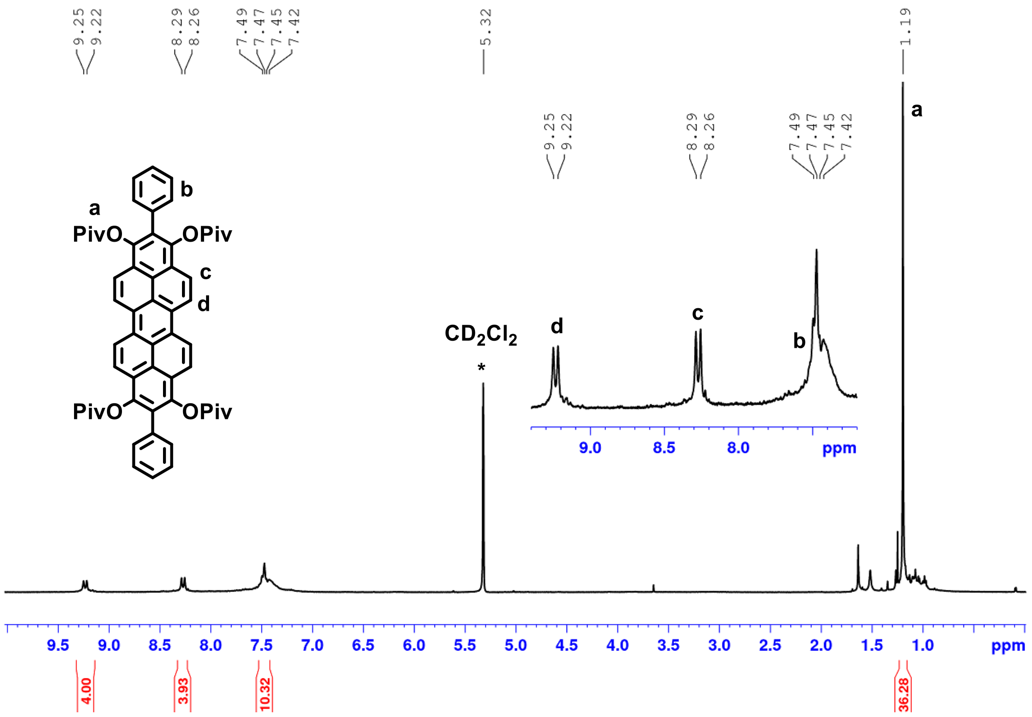

Figure S49. ^{1}H-NMR (300.1 MHz, 298K, CD_2Cl_2*) of **14**.

Single crystal X-ray structures

Crystal Data

	1,3,8,10-tetrakis((triisopropylsilyl)oxy)dibenzo-[*cd*,*lm*]-perylene (**3**)	dibenzo[*cd*,*lm*]perylene-1,3,8,10-tetrayl tetrakis-(trifluoromethanesulfonate) (**5**)	1,3,8,10-tetrakis((di-tert-butylphosphaneyl)oxy)dibenzo-[*cd*,*lm*]perylene (**6**)
CCDC code	2072185	2072186	2072187
Identification code	Swp372loesen	Swp270_0m_a	Swp271loesen
Empirical formula	$C_{62} H_{94} O_4 Si_4$	$C_{30} H_{10} F_{12} O_{12} S_4$	$C_{68} H_{106} O_4 P_4$
Formula weight	1015.73	918.62	1111.40
Temperature	100(2) K	129(2) K	100(2) K
Wavelength	1.54186 Å	0.71073 Å	1.54178 Å
Crystal system	Monoclinic	Triclinic	Triclinic
Space group	$P2_1/c$	*P*-1	*P*-1
Unit cell dimensions	a = 26.4745(6) Å	a = 5.349(7) Å	a = 8.5721(3) Å
	b = 14.4467(2) Å	b = 9.337(13) Å	b = 11.4344(4) Å
	c = 15.4218(4) Å	c = 16.64(2) Å	c = 18.3464(6) Å
	α = 90°.	α = 98.22(3)°.	α = 104.782(2)°.
	β = 92.959(2)°.	β = 90.84(3)°.	β = 93.771(3)°.
	γ = 90°.	γ = 102.55(3)°.	γ = 103.752(3)°.
Volume	5890.5(2) $Å^3$	802.0(18) $Å^3$	1673.53(10) $Å^3$
Z	4	1	1
Density (calculated)	1.145 Mg/m^3	1.902 Mg/m^3	1.103 Mg/m^3
Absorption coefficient	1.272 mm^{-1}	0.435 mm^{-1}	1.369 mm^{-1}
F(000)	2216	458	606
Crystal size	0.293 x 0.239 x 0.133 mm^3	0.362 x 0.226 x 0.090 mm^3	0.245 x 0.207 x 0.172 mm_3
Theta range for data collection	3.486 to 66.500°.	2.260 to 25.726°.	4.205 to 66.581°.
Index ranges	-31<=h<=31, -12<=k<=17, --18<=l<=18	-5<=h<=6, -11<=k<=11, -19<=l<=20	-9<=h<=10, -12<=k<=13, -21<=l<=21
Reflections collected	44168	5077	21553
Independent reflections	10127 [R(int) = 0.0264]	2563 [R(int) = 0.1055]	5753 [R(int) = 0.0249]
Completeness to theta = x	97.4 % (x= 66.500°)	83.9 % (x= 25.242°)	97.5 % (x= 66.581°)
Absorption correction	Semi-empirical from equivalents	Semi-empirical from equivalents	Semi-empirical from equivalents
Max. and min. transmission	0.8299 and 0.3870	0.7453 and 0.5561	0.6362 and 0.2615
Refinement method	Full-matrix least-squares on F^2	Full-matrix least-squares on F^2	Full-matrix least-squares on F^2
Data / restraints / parameters	10127 / 0 / 655	2563 / 0 / 190	5753 / 4 / 405
Goodness-of-fit on F^2	1.086	1.012	1.042
Final R indices [I>2sigma(I)]	R_1 = 0.0676, wR_2 = 0.1786	R_1 = 0.0893, wR_2 = 0.1756	R_1 = 0.0397, wR_2 = 0.1073
R indices (all data)	R_1 = 0.0754, wR_2 = 0.1866	R_1 = 0.1973, wR_2 = 0.2149	R_1 = 0.0454, wR_2 = 0.1164
Extinction coefficient	n/a	n/a	n/a
Largest diff. peak and hole	1.414 and -0.425 e.$Å^{-3}$	0.600 and -0.458 e.$Å^{-3}$	0.325 and -0.323 e.$Å^{-3}$

	1,3,8,10-tetra-phenyldibenzo[*cd,lm*]perylene (**10**)	1,3,8,10-tetrakis(4-(tert-butyl)phenyl)dibenzo[cd,lm]-perylene (**11**)
CCDC code	2072188	2072189
Identification code	Swp289loesen	SWP261loesen
Empirical formula	C_{101} H_{62} Cl_2	C_{66} H_{62}
Formula weight	1346.40	855.15
Temperature	100(2) K	100(2) K
Wavelength	1.54178 Å	1.54178 Å
Crystal system	Triclinic	Triclinic
Space group	*P* -1	*P* -1
Unit cell dimensions	a = 10.7692(2) Å	a = 7.2272(2) Å
	b = 11.8659(2) Å	b = 12.3322(3) Å
	c = 13.7403(2) Å	c = 14.3777(4) Å
	α = 84.6650(10)°	α = 94.579(2)°
	β = 73.7050(10)°	β = 102.773(2)°.
	γ = 80.5050(10)°	γ = 93.028°
Volume	1660.18(5) $Å^3$	1242.47(6) $Å^3$
Z	1	1
Density (calculated)	1.347 Mg/m^3	1.143 Mg/m^3
Absorption coefficient	1.301 mm^{-1}	0.481 mm^{-1}
F(000)	702	458
Crystal size	0.352 x 0.166 x 0.072 mm^3	0.207 x 0.097 x 0.040 mm^3
Theta range for data collection	3.781 to 66.497°.	4.571 to 66.476°
Index ranges	-12<=h<=8, -14<=k<=13, -16<=l<=16	-8<=h<=8, -14<=k<=7, -17<=l<=17
Reflections collected	34715	25218
Independent reflections	5795 [R(int) = 0.0192]	4354 [R(int) = 0.0423]
Completeness to theta = x	98.9 % (x= 66.497°)	99.2 % (x= 66.476°)
Absorption correction	Semi-empirical from equivalents	Semi-empirical from equivalents
Max. and min. transmission	0.4319 and 0.1563	0.8349 and 0.2260
Refinement method	Full-matrix least-squares on F^2	Full-matrix least-squares on F^2
Data / restraints / parameters	5795 / 0 / 479	4354 / 0 / 304
Goodness-of-fit on F^2	1.003	1.036
Final R indices [I>2sigma(I)]	R_1 = 0.0356, wR_2 = 0.1001	R_1 = 0.0532, wR_2 = 0.1450
R indices (all data)	R_1 = 0.0413, wR_2 = 0.1043	R_1 = 0.0711, wR_2 = 0.1595
Extinction coefficient	n/a	n/a
Largest diff. peak and hole	0.163 and -0.303 e.$Å^{-3}$	0.226 and -0.245 e.$Å^{-3}$

Molecular structures of the title compounds

1,3,8,10-tetrakis((triisopropylsilyl)oxy)dibenzo-[*cd*,*lm*]-perylene (3)

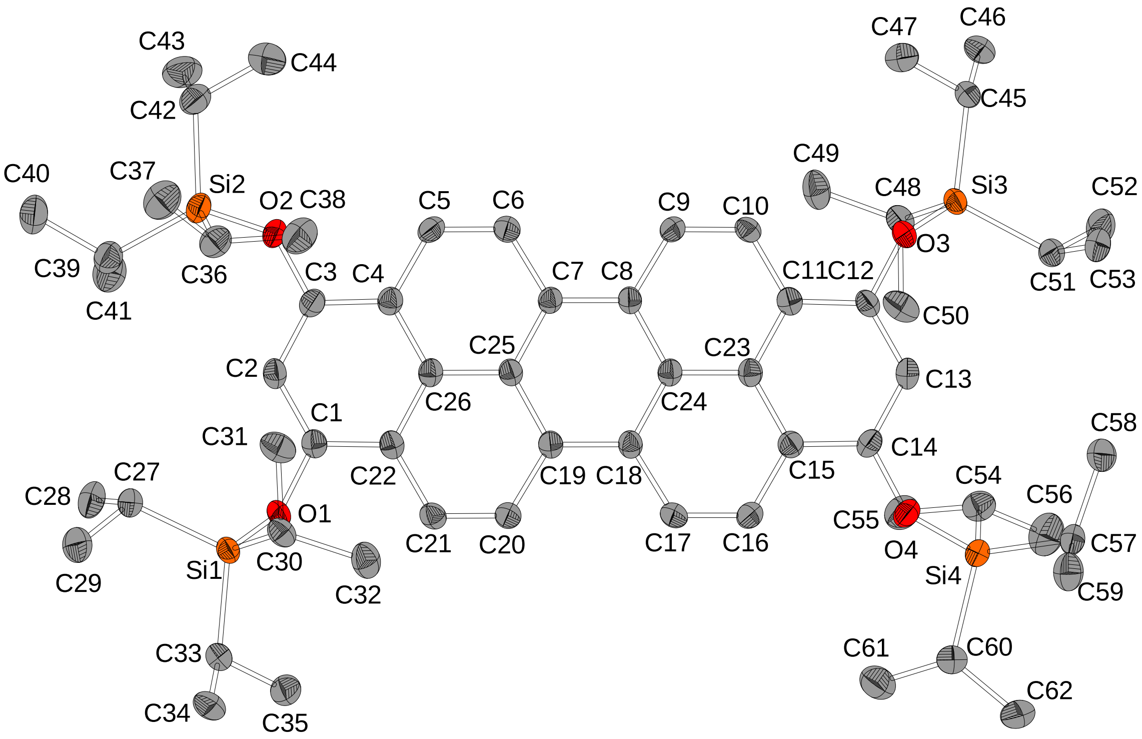

Figure S50. Reduced cell of the crystallographically determined molecular structure of **3**. Protons are not shown.

Table 14. Bond lengths [Å] and angles [°] **3**.

Si(1)-O(1)	1.6762(19)
Si(1)-C(30)	1.879(3)
Si(1)-C(27)	1.880(3)
Si(1)-C(33)	1.882(3)
Si(2)-O(2)	1.679(2)
Si(2)-C(42)	1.873(3)
Si(2)-C(36)	1.883(3)
Si(2)-C(39)	1.902(3)
Si(3)-O(3)	1.6796(19)
Si(3)-C(48)	1.875(3)
Si(3)-C(45)	1.881(3)
Si(3)-C(51)	1.887(3)
Si(4)-O(4)	1.6703(19)
Si(4)-C(54)	1.880(3)
Si(4)-C(60)	1.883(3)
Si(4)-C(57)	1.888(3)
O(1)-C(1)	1.365(3)
O(2)-C(3)	1.365(3)
O(3)-C(12)	1.365(3)
O(4)-C(14)	1.365(3)
C(1)-C(2)	1.393(4)
C(1)-C(22)	1.404(4)
C(2)-C(3)	1.392(4)
C(3)-C(4)	1.402(4)
C(4)-C(26)	1.424(4)

C(4)-C(5)	1.430(4)
C(5)-C(6)	1.353(4)
C(6)-C(7)	1.443(4)
C(7)-C(25)	1.427(4)
C(7)-C(8)	1.427(4)
C(8)-C(24)	1.427(4)
C(8)-C(9)	1.440(4)
C(9)-C(10)	1.352(4)
C(10)-C(11)	1.431(4)
C(11)-C(12)	1.401(4)
C(11)-C(23)	1.421(4)
C(12)-C(13)	1.391(4)
C(13)-C(14)	1.386(4)
C(14)-C(15)	1.404(4)
C(15)-C(23)	1.422(4)
C(15)-C(16)	1.425(4)
C(16)-C(17)	1.349(4)
C(17)-C(18)	1.436(4)
C(18)-C(19)	1.419(4)
C(18)-C(24)	1.426(4)
C(19)-C(25)	1.428(4)
C(19)-C(20)	1.436(4)
C(20)-C(21)	1.346(4)
C(21)-C(22)	1.427(4)
C(22)-C(26)	1.424(4)
C(23)-C(24)	1.434(4)
C(25)-C(26)	1.432(4)
C(27)-C(29)	1.533(4)
C(27)-C(28)	1.533(4)
C(30)-C(31)	1.536(4)
C(30)-C(32)	1.539(4)
C(33)-C(35)	1.535(4)
C(33)-C(34)	1.540(4)
C(36)-C(38)	1.531(4)
C(36)-C(37)	1.553(4)
C(39)-C(40)	1.535(4)
C(39)-C(41)	1.541(4)
C(42)-C(43)	1.522(4)
C(42)-C(44)	1.538(5)
C(45)-C(47)	1.534(4)
C(45)-C(46)	1.536(4)
C(48)-C(50)	1.539(4)
C(48)-C(49)	1.540(4)
C(51)-C(53)	1.531(4)
C(51)-C(52)	1.537(4)
C(54)-C(56)	1.540(4)
C(54)-C(55)	1.548(4)
C(57)-C(58)	1.533(4)
C(57)-C(59)	1.542(4)
C(60)-C(62)	1.533(4)
C(60)-C(61)	1.534(4)
O(1)-Si(1)-C(30)	109.55(11)
O(1)-Si(1)-C(27)	109.09(11)
C(30)-Si(1)-C(27)	111.05(13)
O(1)-Si(1)-C(33)	104.34(11)
C(30)-Si(1)-C(33)	112.18(12)
C(27)-Si(1)-C(33)	110.38(13)
O(2)-Si(2)-C(42)	103.69(12)
O(2)-Si(2)-C(36)	109.02(12)
C(42)-Si(2)-C(36)	113.02(14)
O(2)-Si(2)-C(39)	109.28(12)
C(42)-Si(2)-C(39)	112.83(14)
C(36)-Si(2)-C(39)	108.81(14)
O(3)-Si(3)-C(48)	109.39(12)
O(3)-Si(3)-C(45)	104.06(11)
C(48)-Si(3)-C(45)	112.46(13)
O(3)-Si(3)-C(51)	109.51(11)

C(48)-Si(3)-C(51)	110.05(13)
C(45)-Si(3)-C(51)	111.18(13)
O(4)-Si(4)-C(54)	108.89(12)
O(4)-Si(4)-C(60)	101.83(12)
C(54)-Si(4)-C(60)	117.05(14)
O(4)-Si(4)-C(57)	109.50(12)
C(54)-Si(4)-C(57)	111.17(14)
C(60)-Si(4)-C(57)	107.88(13)
C(1)-O(1)-Si(1)	132.85(17)
C(3)-O(2)-Si(2)	130.70(18)
C(12)-O(3)-Si(3)	132.13(17)
C(14)-O(4)-Si(4)	136.24(18)
O(1)-C(1)-C(2)	121.6(2)
O(1)-C(1)-C(22)	117.6(2)
C(2)-C(1)-C(22)	120.8(2)
C(3)-C(2)-C(1)	120.5(2)
O(2)-C(3)-C(2)	121.6(2)
O(2)-C(3)-C(4)	117.5(2)
C(2)-C(3)-C(4)	120.9(2)
C(3)-C(4)-C(26)	118.9(2)
C(3)-C(4)-C(5)	122.4(2)
C(26)-C(4)-C(5)	118.8(2)
C(6)-C(5)-C(4)	121.3(2)
C(5)-C(6)-C(7)	122.0(3)
C(25)-C(7)-C(8)	119.8(2)
C(25)-C(7)-C(6)	117.9(2)
C(8)-C(7)-C(6)	122.3(2)
C(24)-C(8)-C(7)	119.6(2)
C(24)-C(8)-C(9)	118.2(2)
C(7)-C(8)-C(9)	122.3(2)
C(10)-C(9)-C(8)	122.0(2)
C(9)-C(10)-C(11)	121.2(2)
C(12)-C(11)-C(23)	118.8(2)
C(12)-C(11)-C(10)	122.4(2)
C(23)-C(11)-C(10)	118.8(2)
O(3)-C(12)-C(13)	120.9(2)
O(3)-C(12)-C(11)	118.1(2)
C(13)-C(12)-C(11)	121.0(2)
C(14)-C(13)-C(12)	120.3(2)
O(4)-C(14)-C(13)	122.1(2)
O(4)-C(14)-C(15)	117.0(2)
C(13)-C(14)-C(15)	120.9(2)
C(14)-C(15)-C(23)	118.9(2)
C(14)-C(15)-C(16)	122.1(2)
C(23)-C(15)-C(16)	119.0(2)
C(17)-C(16)-C(15)	121.3(2)
C(16)-C(17)-C(18)	122.0(3)
C(19)-C(18)-C(24)	120.1(2)
C(19)-C(18)-C(17)	121.9(2)
C(24)-C(18)-C(17)	118.1(2)
C(18)-C(19)-C(25)	119.7(2)
C(18)-C(19)-C(20)	122.0(2)
C(25)-C(19)-C(20)	118.3(2)
C(21)-C(20)-C(19)	122.2(2)
C(20)-C(21)-C(22)	120.8(2)
C(1)-C(22)-C(26)	118.8(2)
C(1)-C(22)-C(21)	121.9(2)
C(26)-C(22)-C(21)	119.3(2)
C(11)-C(23)-C(15)	120.0(2)
C(11)-C(23)-C(24)	120.2(2)
C(15)-C(23)-C(24)	119.7(2)
C(18)-C(24)-C(8)	120.4(2)
C(18)-C(24)-C(23)	119.9(2)
C(8)-C(24)-C(23)	119.7(2)
C(7)-C(25)-C(19)	120.4(2)
C(7)-C(25)-C(26)	120.0(2)
C(19)-C(25)-C(26)	119.6(2)

C(4)-C(26)-C(22)	120.2(2)
C(4)-C(26)-C(25)	120.1(2)
C(22)-C(26)-C(25)	119.7(2)
C(29)-C(27)-C(28)	109.9(3)
C(29)-C(27)-Si(1)	110.4(2)
C(28)-C(27)-Si(1)	112.90(19)
C(31)-C(30)-C(32)	110.7(2)
C(31)-C(30)-Si(1)	113.1(2)
C(32)-C(30)-Si(1)	111.5(2)
C(35)-C(33)-C(34)	109.3(2)
C(35)-C(33)-Si(1)	114.3(2)
C(34)-C(33)-Si(1)	111.4(2)
C(38)-C(36)-C(37)	109.8(3)
C(38)-C(36)-Si(2)	114.2(2)
C(37)-C(36)-Si(2)	112.7(2)
C(40)-C(39)-C(41)	109.7(3)
C(40)-C(39)-Si(2)	113.0(2)
C(41)-C(39)-Si(2)	114.3(2)
C(43)-C(42)-C(44)	109.4(3)
C(43)-C(42)-Si(2)	113.1(2)
C(44)-C(42)-Si(2)	113.0(2)
C(47)-C(45)-C(46)	110.0(2)
C(47)-C(45)-Si(3)	114.62(19)
C(46)-C(45)-Si(3)	110.16(19)
C(50)-C(48)-C(49)	110.5(2)
C(50)-C(48)-Si(3)	112.6(2)
C(49)-C(48)-Si(3)	112.2(2)
C(53)-C(51)-C(52)	110.4(2)
C(53)-C(51)-Si(3)	113.29(19)
C(52)-C(51)-Si(3)	112.21(19)
C(56)-C(54)-C(55)	110.3(3)
C(56)-C(54)-Si(4)	112.9(2)
C(55)-C(54)-Si(4)	114.1(2)
C(58)-C(57)-C(59)	109.5(3)
C(58)-C(57)-Si(4)	114.38(19)
C(59)-C(57)-Si(4)	110.8(2)
C(62)-C(60)-C(61)	109.6(3)
C(62)-C(60)-Si(4)	114.1(2)
C(61)-C(60)-Si(4)	115.3(2)

Dibenzo[*cd*,*lm*]perylene-1,3,8,10-tetrayl tetrakis-(trifluoromethanesulfonate) (5)

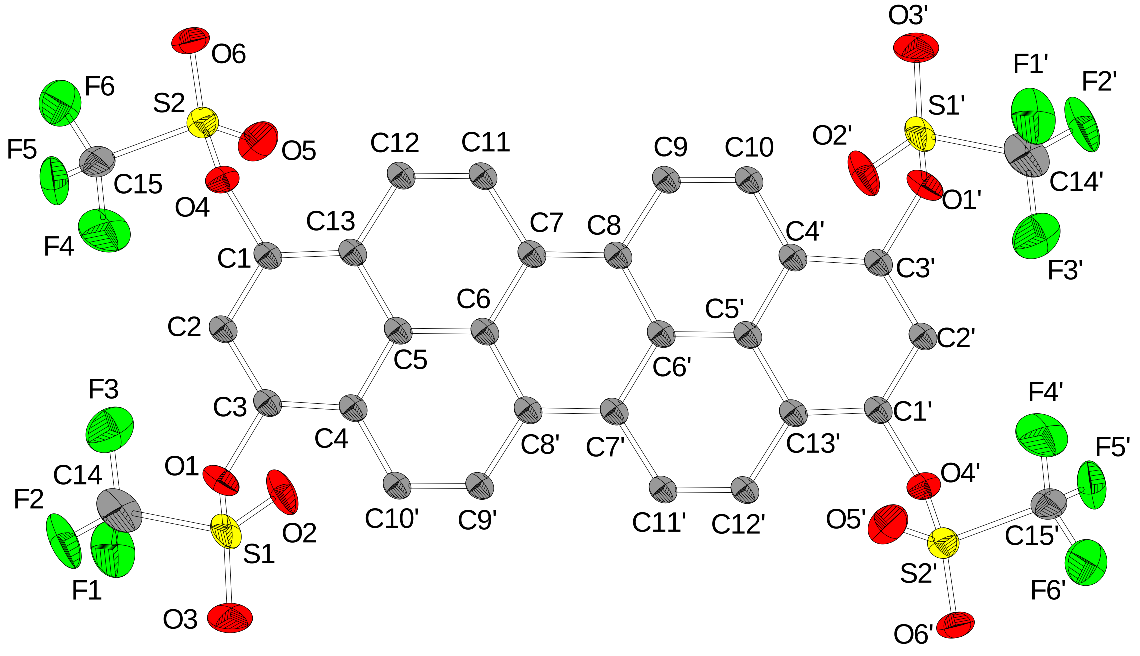

Figure S51. Reduced cell of the crystallographically determined molecular structure of **5**. Protons and solvent molecules are not shown. Symmetry transformations I: 2-x, 1-y, 1-z.

Table 15. Bond lengths [Å] and angles [°] for **5**.

S(1)-O(3)	1.414(6)
S(1)-O(2)	1.416(5)
S(1)-O(1)	1.580(5)
S(1)-C(14)	1.838(12)
C(1)-C(13)	1.364(11)
C(1)-C(2)	1.377(9)
C(1)-O(4)	1.411(9)
F(1)-C(14)	1.327(9)
O(1)-C(3)	1.440(8)
F(3)-C(14)	1.298(10)
C(3)-C(2)	1.369(10)
C(3)-C(4)	1.389(11)
S(2)-O(6)	1.406(5)
S(2)-O(5)	1.425(6)
S(2)-O(4)	1.578(5)
S(2)-C(15)	1.852(10)
F(2)-C(14)	1.306(10)
C(4)-C(10)#1	1.422(10)
C(4)-C(5)	1.429(9)
F(4)-C(15)	1.306(9)
F(6)-C(15)	1.285(10)
C(6)-C(5)	1.400(11)
C(6)-C(8)#1	1.436(10)
C(6)-C(7)	1.445(9)
F(5)-C(15)	1.315(9)
C(5)-C(13)	1.440(11)
C(7)-C(8)	1.393(11)
C(7)-C(11)	1.466(10)
C(9)-C(10)	1.343(11)
C(9)-C(8)	1.433(9)

C(11)-C(12)	1.317(11)
C(12)-C(13)	1.450(9)
O(3)-S(1)-O(2)	123.4(4)
O(3)-S(1)-O(1)	107.0(3)
O(2)-S(1)-O(1)	110.7(3)
O(3)-S(1)-C(14)	107.5(4)
O(2)-S(1)-C(14)	106.7(4)
O(1)-S(1)-C(14)	98.7(4)
C(13)-C(1)-C(2)	124.0(7)
C(13)-C(1)-O(4)	117.8(6)
C(2)-C(1)-O(4)	117.9(7)
C(3)-O(1)-S(1)	119.1(4)
C(2)-C(3)-C(4)	124.3(7)
C(2)-C(3)-O(1)	117.5(7)
C(4)-C(3)-O(1)	118.1(6)
O(6)-S(2)-O(5)	123.1(3)
O(6)-S(2)-O(4)	106.1(3)
O(5)-S(2)-O(4)	111.1(3)
O(6)-S(2)-C(15)	106.0(4)
O(5)-S(2)-C(15)	107.3(4)
O(4)-S(2)-C(15)	101.0(4)
C(3)-C(2)-C(1)	117.0(8)
C(1)-O(4)-S(2)	122.4(4)
C(3)-C(4)-C(10)#1	123.6(7)
C(3)-C(4)-C(5)	117.4(7)
C(10)#1-C(4)-C(5)	119.1(8)
C(5)-C(6)-C(8)#1	120.1(6)
C(5)-C(6)-C(7)	121.1(6)
C(8)#1-C(6)-C(7)	118.7(7)
C(6)-C(5)-C(4)	120.2(7)
C(6)-C(5)-C(13)	121.0(6)
C(4)-C(5)-C(13)	118.8(8)
C(8)-C(7)-C(6)	120.8(6)
C(8)-C(7)-C(11)	123.4(6)
C(6)-C(7)-C(11)	115.8(7)
C(10)-C(9)-C(8)	122.8(7)
C(7)-C(8)-C(9)	122.1(7)
C(7)-C(8)-C(6)#1	120.5(6)
C(9)-C(8)-C(6)#1	117.3(8)
C(9)-C(10)-C(4)#1	120.4(7)
C(12)-C(11)-C(7)	122.9(7)
C(11)-C(12)-C(13)	122.3(7)
C(1)-C(13)-C(5)	118.4(6)
C(1)-C(13)-C(12)	124.8(7)
C(5)-C(13)-C(12)	116.8(7)
F(3)-C(14)-F(2)	111.5(9)
F(3)-C(14)-F(1)	109.9(8)
F(2)-C(14)-F(1)	109.5(7)
F(3)-C(14)-S(1)	109.3(6)
F(2)-C(14)-S(1)	109.9(7)
F(1)-C(14)-S(1)	106.6(8)
F(6)-C(15)-F(4)	111.3(8)
F(6)-C(15)-F(5)	110.7(8)
F(4)-C(15)-F(5)	107.5(6)
F(6)-C(15)-S(2)	107.9(6)
F(4)-C(15)-S(2)	109.8(7)
F(5)-C(15)-S(2)	109.7(6)

Symmetry transformations used to generate equivalent atoms:
#1 -x+2,-y+1,-z+1

1,3,8,10-tetrakis((di-tert-butylphosphaneyl)oxy)dibenzo-[*cd*,*lm*]perylene (6)

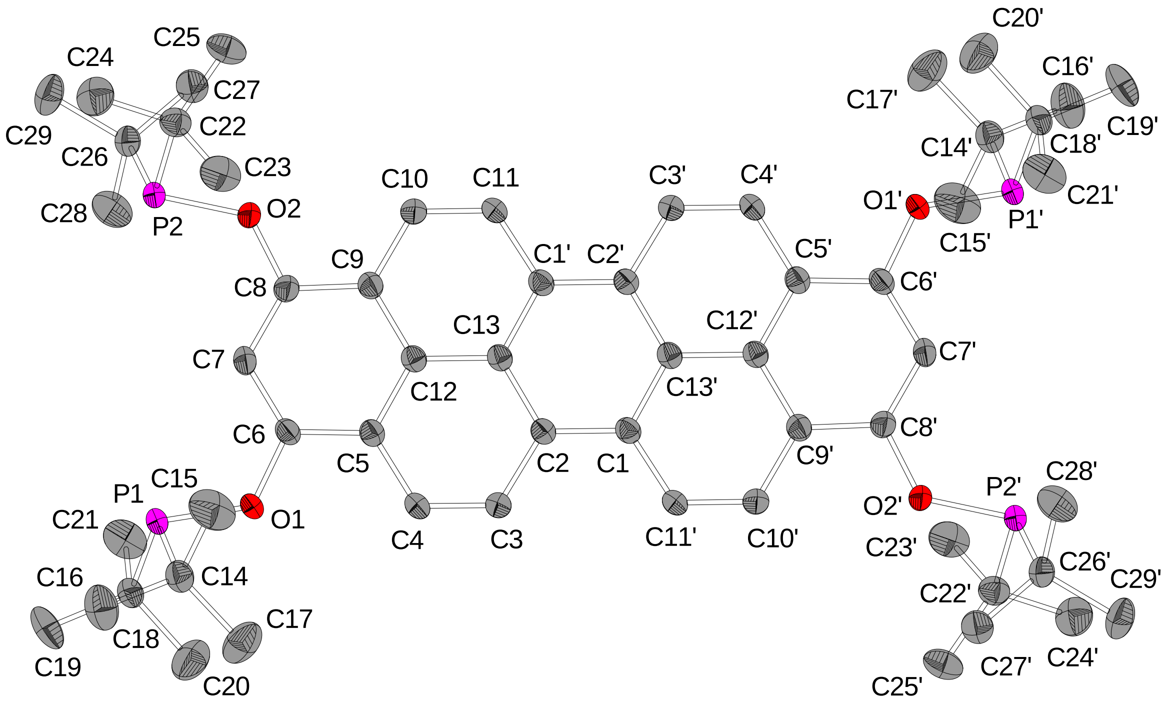

Figure S52. Reduced cell of the crystallographically determined molecular structure of **6**. Protons and solvent molecules are not shown. Symmetry operations I: 1-x, -y, -z.

Table 16. Bond lengths [Å] and angles [°] for **6**.

P(1)-O(1)	1.6780(11)
P(1)-C(18)	1.8662(17)
P(1)-C(14)	1.8761(17)
O(1)-C(6)	1.3787(18)
C(1)-C(2)	1.418(2)
C(1)-C(13)#1	1.427(2)
C(1)-C(11)#1	1.434(2)
P(2)-O(2)	1.6752(11)
P(2)-C(26)	1.8710(16)
P(2)-C(22)	1.8723(17)
O(2)-C(8)	1.3800(19)
C(2)-C(13)	1.425(2)
C(2)-C(3)	1.439(2)
C(3)-C(4)	1.351(2)
C(4)-C(5)	1.429(2)
C(5)-C(6)	1.405(2)
C(5)-C(12)	1.419(2)
C(6)-C(7)	1.385(2)
C(7)-C(8)	1.386(2)
C(9)-C(8)	1.403(2)
C(9)-C(12)	1.420(2)
C(9)-C(10)	1.426(2)
C(10)-C(11)	1.349(2)
C(12)-C(13)	1.435(2)
C(14)-C(17)	1.518(3)
C(14)-C(16)	1.524(2)
C(14)-C(15)	1.538(3)
C(18)-C(20)	1.525(3)
C(18)-C(19)	1.529(2)
C(18)-C(21)	1.532(2)

C(22)-C(25)	1.527(2)
C(22)-C(24)	1.533(2)
C(22)-C(23)	1.538(2)
C(26)-C(27)	1.529(2)
C(26)-C(29)	1.532(2)
C(26)-C(28)	1.535(2)
C(30)-C(31)	1.541(7)
C(31)-C(32)	1.509(6)
C(32)-C(33)	1.499(7)
C(33)-C(34)	1.528(7)
C(35)-C(36)	1.549(9)
C(36)-C(37)	1.499(13)
C(37)-C(38)	1.538(9)
C(39)-C(38)	1.489(19)
O(1)-P(1)-C(18)	95.86(7)
O(1)-P(1)-C(14)	97.65(7)
C(18)-P(1)-C(14)	111.04(8)
C(6)-O(1)-P(1)	123.70(10)
C(2)-C(1)-C(13)#1	119.57(14)
C(2)-C(1)-C(11)#1	122.34(14)
C(13)#1-C(1)-C(11)#1	118.09(14)
O(2)-P(2)-C(26)	95.92(7)
O(2)-P(2)-C(22)	98.00(7)
C(26)-P(2)-C(22)	111.15(8)
C(8)-O(2)-P(2)	125.00(10)
C(1)-C(2)-C(13)	119.93(14)
C(1)-C(2)-C(3)	122.04(14)
C(13)-C(2)-C(3)	118.03(14)
C(4)-C(3)-C(2)	121.95(15)
C(3)-C(4)-C(5)	121.33(14)
C(6)-C(5)-C(12)	118.58(14)
C(6)-C(5)-C(4)	122.73(14)
C(12)-C(5)-C(4)	118.68(14)
O(1)-C(6)-C(7)	123.10(13)
O(1)-C(6)-C(5)	114.89(14)
C(7)-C(6)-C(5)	121.98(14)
C(6)-C(7)-C(8)	118.85(14)
C(8)-C(9)-C(12)	118.55(14)
C(8)-C(9)-C(10)	122.44(15)
C(12)-C(9)-C(10)	119.00(14)
O(2)-C(8)-C(7)	123.11(14)
O(2)-C(8)-C(9)	114.75(13)
C(7)-C(8)-C(9)	122.09(15)
C(11)-C(10)-C(9)	121.11(15)
C(10)-C(11)-C(1)#1	122.17(15)
C(5)-C(12)-C(9)	119.93(14)
C(5)-C(12)-C(13)	120.13(14)
C(9)-C(12)-C(13)	119.94(14)
C(2)-C(13)-C(1)#1	120.50(14)
C(2)-C(13)-C(12)	119.83(14)
C(1)#1-C(13)-C(12)	119.67(14)
C(17)-C(14)-C(16)	110.87(16)
C(17)-C(14)-C(15)	107.79(17)
C(16)-C(14)-C(15)	108.05(16)
C(17)-C(14)-P(1)	117.20(13)
C(16)-C(14)-P(1)	108.98(12)
C(15)-C(14)-P(1)	103.35(12)
C(20)-C(18)-C(19)	110.69(17)
C(20)-C(18)-C(21)	108.69(17)
C(19)-C(18)-C(21)	107.95(15)
C(20)-C(18)-P(1)	116.20(12)
C(19)-C(18)-P(1)	109.20(12)
C(21)-C(18)-P(1)	103.62(12)
C(25)-C(22)-C(24)	110.50(15)
C(25)-C(22)-C(23)	108.93(14)
C(24)-C(22)-C(23)	107.98(15)
C(25)-C(22)-P(2)	117.12(12)

C(24)-C(22)-P(2)	108.33(12)
C(23)-C(22)-P(2)	103.46(12)
C(27)-C(26)-C(29)	110.50(15)
C(27)-C(26)-C(28)	108.11(14)
C(29)-C(26)-C(28)	108.38(15)
C(27)-C(26)-P(2)	116.44(11)
C(29)-C(26)-P(2)	109.27(12)
C(28)-C(26)-P(2)	103.68(12)
C(32)-C(31)-C(30)	112.8(5)
C(33)-C(32)-C(31)	114.0(4)
C(32)-C(33)-C(34)	113.0(6)
C(37)-C(36)-C(35)	111.9(7)
C(36)-C(37)-C(38)	111.9(7)
C(39)-C(38)-C(37)	112.4(8)

Symmetry transformations used to generate equivalent atoms:
#1 -x+1,-y,-z

1,3,8,10-tetraphenyldibenzo[*cd*,*lm*]perylene (10)

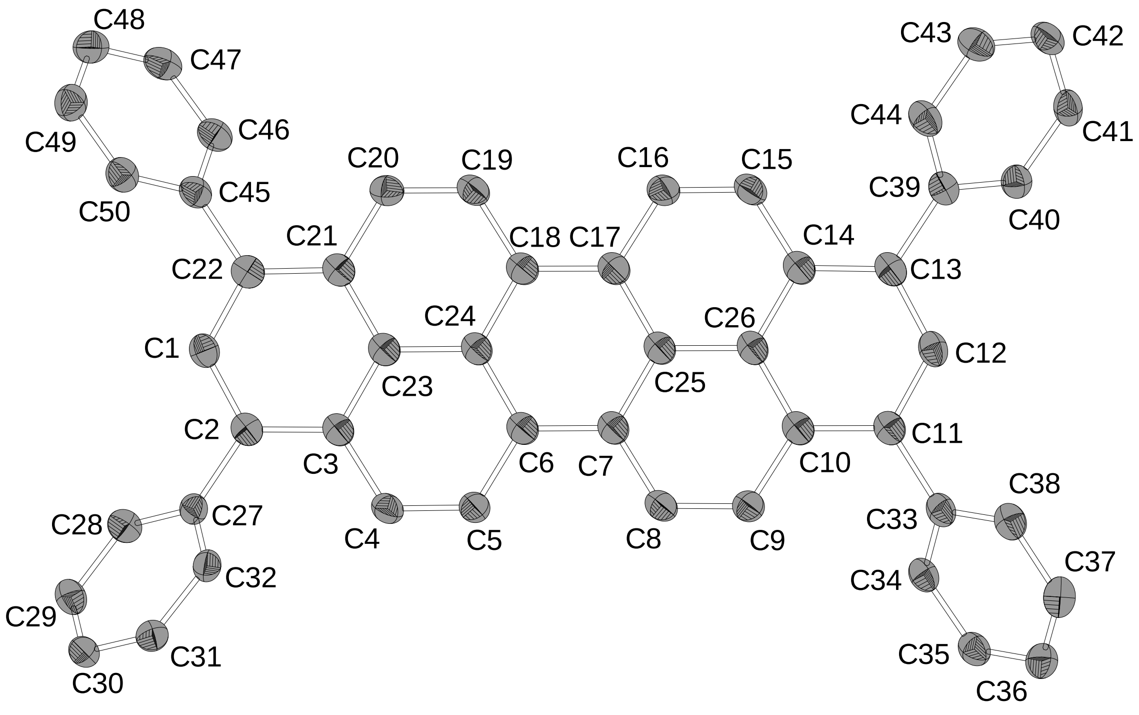

Figure S53. Reduced cell of the crystallographically determined molecular structure of **10**. Protons and solvent molecules are not shown.

Table 17. Bond lengths [Å] and angles [°] for **10**.

Cl(1)-C(52)	1.728(5)
Cl(2)-C(52)	1.771(5)
C(1)-C(22)	1.3925(17)
C(1)-C(2)	1.3945(17)
C(2)-C(3)	1.4144(17)
C(2)-C(27)	1.4894(16)
C(3)-C(23)	1.4266(17)
C(3)-C(4)	1.4317(17)
C(4)-C(5)	1.3515(18)
C(5)-C(6)	1.4273(17)
C(6)-C(7)	1.4181(18)

C(6)-C(24)	1.4259(17)
C(7)-C(25)	1.4233(17)
C(7)-C(8)	1.4321(17)
C(8)-C(9)	1.3502(18)
C(9)-C(10)	1.4335(17)
C(10)-C(11)	1.4124(18)
C(10)-C(26)	1.4261(17)
C(11)-C(12)	1.3909(17)
C(11)-C(33)	1.4915(17)
C(12)-C(13)	1.3936(18)
C(13)-C(14)	1.4148(18)
C(13)-C(39)	1.4914(16)
C(14)-C(26)	1.4288(17)
C(14)-C(15)	1.4320(18)
C(15)-C(16)	1.3510(19)
C(16)-C(17)	1.4279(17)
C(17)-C(18)	1.4225(18)
C(17)-C(25)	1.4245(17)
C(18)-C(24)	1.4215(17)
C(18)-C(19)	1.4331(17)
C(19)-C(20)	1.3504(18)
C(20)-C(21)	1.4334(17)
C(21)-C(22)	1.4142(18)
C(21)-C(23)	1.4261(17)
C(22)-C(45)	1.4892(17)
C(23)-C(24)	1.4392(17)
C(25)-C(26)	1.4399(18)
C(27)-C(28)	1.3921(18)
C(27)-C(32)	1.4004(17)
C(28)-C(29)	1.3871(17)
C(29)-C(30)	1.3830(18)
C(30)-C(31)	1.3833(18)
C(31)-C(32)	1.3882(17)
C(33)-C(38)	1.3898(18)
C(33)-C(34)	1.4044(17)
C(34)-C(35)	1.3826(18)
C(35)-C(36)	1.3885(19)
C(36)-C(37)	1.3834(19)
C(37)-C(38)	1.3882(19)
C(39)-C(44)	1.3967(18)
C(39)-C(40)	1.4030(17)
C(40)-C(41)	1.3878(17)
C(41)-C(42)	1.3875(19)
C(42)-C(43)	1.3841(18)
C(43)-C(44)	1.3866(17)
C(45)-C(46)	1.3979(17)
C(45)-C(50)	1.4005(18)
C(46)-C(47)	1.3870(18)
C(47)-C(48)	1.386(2)
C(48)-C(49)	1.3889(19)
C(49)-C(50)	1.3872(18)
Cl(1)-C(52)-Cl(2)	112.3(2)
C(22)-C(1)-C(2)	123.01(12)
C(1)-C(2)-C(3)	118.65(11)
C(1)-C(2)-C(27)	118.44(11)
C(3)-C(2)-C(27)	122.90(11)
C(2)-C(3)-C(23)	119.55(11)
C(2)-C(3)-C(4)	122.41(11)
C(23)-C(3)-C(4)	118.02(11)
C(5)-C(4)-C(3)	121.48(11)
C(4)-C(5)-C(6)	122.35(11)
C(7)-C(6)-C(24)	120.16(11)
C(7)-C(6)-C(5)	122.00(11)
C(24)-C(6)-C(5)	117.81(11)
C(6)-C(7)-C(25)	120.12(11)
C(6)-C(7)-C(8)	122.16(11)
C(25)-C(7)-C(8)	117.72(11)

C(9)-C(8)-C(7)	122.04(11)
C(8)-C(9)-C(10)	121.64(11)
C(11)-C(10)-C(26)	119.41(11)
C(11)-C(10)-C(9)	122.56(11)
C(26)-C(10)-C(9)	118.00(11)
C(12)-C(11)-C(10)	119.03(11)
C(12)-C(11)-C(33)	118.87(11)
C(10)-C(11)-C(33)	122.10(11)
C(11)-C(12)-C(13)	122.98(12)
C(12)-C(13)-C(14)	118.99(11)
C(12)-C(13)-C(39)	118.54(11)
C(14)-C(13)-C(39)	122.45(11)
C(13)-C(14)-C(26)	119.29(11)
C(13)-C(14)-C(15)	123.00(11)
C(26)-C(14)-C(15)	117.59(11)
C(16)-C(15)-C(14)	121.84(11)
C(15)-C(16)-C(17)	122.58(12)
C(18)-C(17)-C(25)	120.29(11)
C(18)-C(17)-C(16)	122.12(11)
C(25)-C(17)-C(16)	117.58(12)
C(24)-C(18)-C(17)	119.88(11)
C(24)-C(18)-C(19)	117.83(11)
C(17)-C(18)-C(19)	122.29(11)
C(20)-C(19)-C(18)	122.20(11)
C(19)-C(20)-C(21)	121.67(11)
C(22)-C(21)-C(23)	119.19(11)
C(22)-C(21)-C(20)	122.62(11)
C(23)-C(21)-C(20)	118.01(11)
C(1)-C(22)-C(21)	119.15(11)
C(1)-C(22)-C(45)	118.06(11)
C(21)-C(22)-C(45)	122.77(11)
C(21)-C(23)-C(3)	120.16(11)
C(21)-C(23)-C(24)	119.96(11)
C(3)-C(23)-C(24)	119.87(11)
C(18)-C(24)-C(6)	119.82(11)
C(18)-C(24)-C(23)	120.20(11)
C(6)-C(24)-C(23)	119.98(11)
C(7)-C(25)-C(17)	119.61(11)
C(7)-C(25)-C(26)	120.22(11)
C(17)-C(25)-C(26)	120.16(11)
C(10)-C(26)-C(14)	120.09(11)
C(10)-C(26)-C(25)	119.64(11)
C(14)-C(26)-C(25)	120.24(11)
C(28)-C(27)-C(32)	117.95(11)
C(28)-C(27)-C(2)	119.91(11)
C(32)-C(27)-C(2)	122.03(11)
C(29)-C(28)-C(27)	121.15(12)
C(30)-C(29)-C(28)	120.40(12)
C(29)-C(30)-C(31)	119.19(12)
C(30)-C(31)-C(32)	120.69(12)
C(31)-C(32)-C(27)	120.60(12)
C(38)-C(33)-C(34)	118.34(12)
C(38)-C(33)-C(11)	120.10(11)
C(34)-C(33)-C(11)	121.48(12)
C(35)-C(34)-C(33)	120.50(12)
C(34)-C(35)-C(36)	120.47(12)
C(37)-C(36)-C(35)	119.46(12)
C(36)-C(37)-C(38)	120.28(13)
C(37)-C(38)-C(33)	120.90(12)
C(44)-C(39)-C(40)	117.73(11)
C(44)-C(39)-C(13)	122.12(11)
C(40)-C(39)-C(13)	120.12(11)
C(41)-C(40)-C(39)	120.94(12)
C(42)-C(41)-C(40)	120.41(11)
C(43)-C(42)-C(41)	119.23(11)
C(42)-C(43)-C(44)	120.56(12)
C(43)-C(44)-C(39)	121.09(12)

C(46)-C(45)-C(50)	117.98(11)
C(46)-C(45)-C(22)	121.61(11)
C(50)-C(45)-C(22)	120.33(11)
C(47)-C(46)-C(45)	121.19(12)
C(48)-C(47)-C(46)	120.08(12)
C(47)-C(48)-C(49)	119.61(12)
C(50)-C(49)-C(48)	120.32(12)
C(49)-C(50)-C(45)	120.81(12)

1,3,8,10-tetrakis(4-(tert-butyl)phenyl)dibenzo[*cd*,*lm*]-perylene (11)

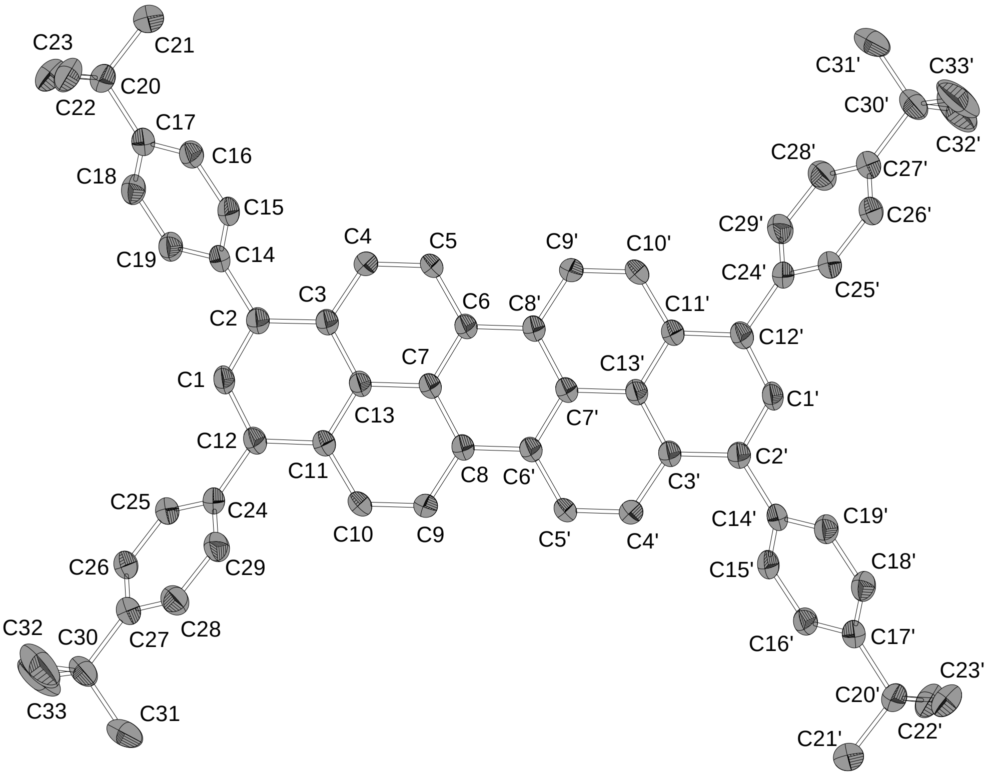

Figure S54. Reduced cell of the crystallographically determined molecular structure of **11**. Protons and solvent molecules are not shown. Symmetry operations I: 2-x, -y, 1-z.

Table 18. Bond lengths [Å] and angles [°] for **11**.

C(1)-C(12)	1.387(2)
C(1)-C(2)	1.391(2)
C(2)-C(3)	1.417(2)
C(2)-C(14)	1.484(2)
C(3)-C(13)	1.426(2)
C(3)-C(4)	1.427(2)
C(4)-C(5)	1.353(2)
C(5)-C(6)	1.431(2)
C(6)-C(8)#1	1.420(2)
C(6)-C(7)	1.423(2)
C(7)-C(8)	1.421(2)
C(7)-C(13)	1.441(2)
C(8)-C(9)	1.433(2)
C(9)-C(10)	1.352(2)
C(10)-C(11)	1.433(2)
C(11)-C(12)	1.414(2)
C(11)-C(13)	1.421(2)
C(12)-C(24)	1.486(2)
C(14)-C(19)	1.390(3)

C(14)-C(15)	1.402(3)
C(15)-C(16)	1.384(3)
C(16)-C(17)	1.399(3)
C(17)-C(18)	1.389(3)
C(17)-C(20)	1.535(2)
C(18)-C(19)	1.389(3)
C(20)-C(23)	1.531(3)
C(20)-C(21)	1.534(3)
C(20)-C(22)	1.534(3)
C(24)-C(25)	1.387(3)
C(24)-C(29)	1.396(3)
C(25)-C(26)	1.384(3)
C(26)-C(27)	1.389(3)
C(27)-C(28)	1.398(3)
C(27)-C(30)	1.536(3)
C(28)-C(29)	1.382(3)
C(30)-C(33)	1.519(3)
C(30)-C(31)	1.524(3)
C(30)-C(32)	1.533(4)
C(12)-C(1)-C(2)	123.10(16)
C(1)-C(2)-C(3)	118.69(16)
C(1)-C(2)-C(14)	118.68(15)
C(3)-C(2)-C(14)	122.59(16)
C(2)-C(3)-C(13)	119.54(16)
C(2)-C(3)-C(4)	122.45(16)
C(13)-C(3)-C(4)	117.99(15)
C(5)-C(4)-C(3)	121.80(16)
C(4)-C(5)-C(6)	121.89(16)
C(8)#1-C(6)-C(7)	119.98(16)
C(8)#1-C(6)-C(5)	122.07(16)
C(7)-C(6)-C(5)	117.96(15)
C(8)-C(7)-C(6)	120.09(16)
C(8)-C(7)-C(13)	120.01(16)
C(6)-C(7)-C(13)	119.89(16)
C(6)#1-C(8)-C(7)	119.84(16)
C(6)#1-C(8)-C(9)	122.19(16)
C(7)-C(8)-C(9)	117.96(15)
C(10)-C(9)-C(8)	121.80(17)
C(9)-C(10)-C(11)	121.75(16)
C(12)-C(11)-C(13)	119.41(16)
C(12)-C(11)-C(10)	122.55(16)
C(13)-C(11)-C(10)	118.04(15)
C(1)-C(12)-C(11)	119.12(16)
C(1)-C(12)-C(24)	118.58(15)
C(11)-C(12)-C(24)	122.19(16)
C(11)-C(13)-C(3)	120.04(15)
C(11)-C(13)-C(7)	119.99(16)
C(3)-C(13)-C(7)	119.96(16)
C(19)-C(14)-C(15)	117.11(17)
C(19)-C(14)-C(2)	120.19(17)
C(15)-C(14)-C(2)	122.64(16)
C(16)-C(15)-C(14)	121.01(17)
C(15)-C(16)-C(17)	121.78(18)
C(18)-C(17)-C(16)	116.98(17)
C(18)-C(17)-C(20)	122.85(16)
C(16)-C(17)-C(20)	120.16(17)
C(19)-C(18)-C(17)	121.43(17)
C(18)-C(19)-C(14)	121.66(18)
C(23)-C(20)-C(21)	108.78(18)
C(23)-C(20)-C(22)	108.04(16)
C(21)-C(20)-C(22)	109.53(19)
C(23)-C(20)-C(17)	112.21(16)
C(21)-C(20)-C(17)	109.38(15)
C(22)-C(20)-C(17)	108.86(16)
C(25)-C(24)-C(29)	117.22(17)
C(25)-C(24)-C(12)	120.58(17)
C(29)-C(24)-C(12)	122.04(17)

C(26)-C(25)-C(24)	121.74(18)
C(25)-C(26)-C(27)	121.39(18)
C(26)-C(27)-C(28)	116.88(17)
C(26)-C(27)-C(30)	123.01(18)
C(28)-C(27)-C(30)	120.11(18)
C(29)-C(28)-C(27)	121.74(19)
C(28)-C(29)-C(24)	121.01(18)
C(33)-C(30)-C(31)	108.2(2)
C(33)-C(30)-C(32)	108.3(2)
C(31)-C(30)-C(32)	109.5(2)
C(33)-C(30)-C(27)	112.83(18)
C(31)-C(30)-C(27)	109.47(17)
C(32)-C(30)-C(27)	108.56(19)

Symmetry transformations used to generate equivalent atoms:
#1 -x+2,-y,-z+1

References

[1] W. L. F. Armarego, D. D. Perrin, *Purification of laboratory chemicals*, Butterworth-Heinemann, Oxford, **2002**.
[2] B. Eistert, W. Eifler, O. Ganster, *Chem. Ber.* **1969**, *102*, 1988–2002.
[3] N. Buffet, E. Grelet, H. Bock, *Chem. Eur. J.* **2010**, *16*, 5549–5553.
[4] D. T. W. van Stadlbauer, *Molecules* **1997**, *1*, 201–206.
[5] G. M. Sheldrick, *Acta Cryst. C Section C* **2015**, *71*, 3–8.
[6] C. B. Hübschle, G. M. Sheldrick, B. Dittrich, *J. Appl. Cryst.* **2011**, *44*, 1281–1284.
[7] A. L. Spek, *Acta Cryst. D* **2009**, *65*, 148–155.
[8] H. P. K. Brandenburg, *Diamond*, Crystal Impact GbR, Bonn, **2012**.
[9] A. D. Becke, *J. Chem. Phys.* **1993**, *98*, 5648–5652.
[10] Lee, Yang, Parr, *Phys. Rev. B* **1988**, *37*, 785–789.
[11] P. J. Stephens, F. J. Devlin, C. S. Ashvar, C. F. Chabalowski, M. J. Frisch, *Faraday Disc.* **1994**, *99*, 103.
[12] A. Schäfer, C. Huber, R. Ahlrichs, *J. Chem. Phys.* **1994**, *100*, 5829–5835.
[13] F. Weigend, *Phys. Chem. Chem. Phys.* **2006**, *8*, 1057–1065.
[14] F. Weigend, R. Ahlrichs, *Phys. Chem. Chem. Phys.* **2005**, *7*, 3297–3305.
[15] F. Weigend, M. Häser, H. Patzelt, R. Ahlrichs, *Chem. Phys. Lett.* **1998**, *294*, 143–152.
[16] K. Eichkorn, O. Treutler, H. Öhm, M. Häser, R. Ahlrichs, *Chem. Phys. Lett.* **1995**, *240*, 283–290.
[17] M. von Arnim, R. Ahlrichs, *J. Comput. Chem.* **1998**, *19*, 1746–1757.
[18] F. Weigend, *Phys. Chem. Chem. Phys.* **2002**, *4*, 4285–4291.
[19] S. Grimme, J. Antony, S. Ehrlich, H. Krieg, *J. Chem. Phys.* **2010**, *132*, 154104.
[20] A. D. Becke, E. R. Johnson, *J. Chem. Phys.* **2005**, *123*, 154101.
[21] E. R. Johnson, A. D. Becke, *J. Chem. Phys.* **2005**, *123*, 24101.
[22] E. R. Johnson, A. D. Becke, *J. Chem. Phys.* **2006**, *124*, 174104.
[23] S. Grimme, S. Ehrlich, L. Goerigk, *J. Comput. Chem.* **2011**, *32*, 1456–1465.
[24] F. Neese, *WIREs Comput Mol Sci* **2011**, *2*, 73–78.
[25] Perdew, Burke, Ernzerhof, *Phys. Rev. Lett.* **1996**, *77*, 3865–3868.
[26] J. P. Perdew, K. Burke, M. Ernzerhof, *Phys. Rev. Lett.* **1997**, *78*, 1396.
[27] J. R. Lakowicz, *Principles of fluorescence spectroscopy*, Springer, New York, **2010**.
[28] S. Werner, T. Vollgraff, J. Sundermeyer, *Angew. Chem.* **2021**. DOI: 10.1002/ange.202100686; *Angew. Chem. Int. Ed.* **2021**, DOI: 10.1002/anie.202100686.
